# Recombinant anti-HIV MAP30, a ribosome inactivating protein: against plant virus and bacteriophage

**DOI:** 10.1038/s41598-023-29365-7

**Published:** 2023-02-06

**Authors:** Nafiseh Amirzadeh, Ali Moghadam, Ali Niazi, Alireza Afsharifar

**Affiliations:** 1 Institute of Biotechnology, College of Agriculture, Shiraz University, Shiraz, Iran; 2 Center of Plant Virology Research, College of Agriculture, Shiraz University, Shiraz, Iran

**Keywords:** Biotechnology, Plant sciences

## Abstract

The ribosome inactivating proteins (RIPs) efficiently decrease the microbial infections in plants. *Momordica* *charantia* MAP30 is a type I RIP that has not been investigated against plant viruses or bacteriophages. To evaluate of these activities, the recombinant MAP30 (rMAP30) was produced in the hairy roots of *Nicotiana* *tabacum*. Inoculation of 3 μg of transgenic total protein or 0.6 μg of rMAP30 against 0.1 μg of TMV reduced the leaf necrotic spots to 78.23% and 82.72%, respectively. The treatment of 0.1 μg of CMV with rMAP30 (0.6 μg) showed the reduction in the leaf necrotic spots to 85.8%. While the infection was increased after rMAP30 dilution. In the time interval assays, the leaves were first inoculated with 1 μg of rMAP30 or 0.1 μg of purified TMV or CMV agent for 6 h, then virus or protein was applied in order. This led the spot reduction to 35.22% and 67% for TMV, and 38.61% and 55.31% for CMV, respectively. In both the pre- and co-treatments of 1:10 or 1:20 diluted bacteriophage with 15 μg of transgenic total protein, the number and diameter of the plaques were reduced. The results showed that the highest inhibitory effect was observed in the pre-treatment assay of bacteriophage with transgenic total protein for 24 h. The decrease in the growth of bacteriophage caused more growth pattern of *Escherichia* *coli.* The results confirm that rMAP30 shows antibacterial activity against *Streptococcus* *aureus* and *E.* *coli*, antifungal activity against *Candida* *albicans*, and antiviral activity against CMV and TMV. Moreover, rMAP30 exhibits anti-phage activity for the first time. According to our findings, rMAP30 might be a valuable preservative agent in foods and beverages in the food industry as well as an antiviral and antimicrobial mixture in agriculture.

## Introduction

Many viruses have circulated eukaryotic and prokaryotic hosts. Due to the fact that there are the infection risks resulted from the plant viruses and bacteriophages, it would be necessary to achieve a more accurate management through applying integrated and multidisciplinary approaches. According to the estimations, at least 10% of the global food production is lost due to plant diseases^1^. Plants are targeted by many pathogens, including viruses, bacteria, and fungi^2,3^. Tobacco mosaic virus (TMV) and cucumber mosaic virus (CMV), and 3 others, are the top five disruptive plant viruses^4^.

The CMV alters the chemistry of the many hosts, affecting insect vectors involved in virus transmission^5^. TMV is the first discovered virus that infects more than 150 different species^6^. TMV is transmitted by mechanical inoculation, grafting, and by many species of aphids in a no persistent manner^7^, and induces mosaic-like mottling and discoloration on the leaves^8^.

Although, the plant viruses are one of the most disruptive pathogens in agriculture, bacteriophages are the other viruses considered as "enemy" in various industries for decades^9,10^. Phages have showed the harmful effects on the food and pharmaceutical industries. However, the biggest problem caused by the presence of phages is explained in the dairy industry^10^. Some strategies of phage prevention are adopted in dairy industry to minimizing the risk of fermentation failures^11^. For instance, starter culture rotation^12^, and membrane filtration or UV treatment in combination with thermal treatment^11^ are commonly employed to control the phage invasion.

Our opinion in this research is whether a plant protein can show anti-phage activity or not. Therefore, we considered a famous plant protein named MAP30 (Momordica Antiviral Protein 30 kDa) for further study. The anti-HIV and anti-HSV, anti-dengue fever virus^13,14,30^, as well as the anti-cancer activities of MAP30 against human cancer cell lines such as brain glioblastoma, melanoma, prostate carcinoma, breast carcinoma, liver hepatoma, colorectal carcinoma^15^, and even liver tumor in both In vivo and In vitro have been confirmed^14^, but the antiviral activity of this protein against plant viruses and phages is not more studied.

The MAP30, a ribosome inactivating protein (RIP) isolated from the fruit and seeds of *Momordica* *charantia*^16,17^. The In vitro and In vivo studies have confirmed that the extracts and MAP30 analogs, isolated from *M.* *charantia*, show broad-spectrum anti-microbial activities^18,19^. It is noteworthy that MAP30 possesses various biological activities such as interaction with viral-infected ribosomes to inhibit the protein biosynthesis in the infected cells^20,21^. Furthermore, MAP30 shows dual ability to cleave both DNA and RNA substrates^22^. The RIPs present in many plants, both monocotyledonous and dicotyledonous^15,23–26^.

These proteins are mainly divided into 2 groups. Type 1 like MAP30, consisting of a polypeptide chain with N-glycosylase activity, and type 2 RIPs, formed by two polypeptide chains, an A (active) chain with enzymatic activity, and a B (binding) chain with lectin. These inhibitors cleave the glycosidic linkage between adenine and ribose in a highly conserved SR (α-sarcin/ricin) loop within 28S rRNA. This cleavage prevents the binding of elongation factors during the translation process and consequently inhibits the protein synthesis^27^. Based on their diverse activities, RIPs, alone or as part of a conjugate, can be a desirable candidates for developing selective antiviral and anticancer agents^15^. Based on our opinion, activity of recombinant MAP30 (rMAP30) expressed in *Nicotiana* *tabacum* hairy roots against viral, bacterial, and fungal microorganisms as well as against *E.* *coli* phase was investigated.

## Materials and methods

### Growth conditions

Seeds of Turkish *N.* *tabacum* *L.* cv. were achieved from the Center of Plant Virology Research, College of Agriculture, Shiraz University. The permissions were obtained to use these seeds. The seeds were sterilized^19,28^ and grown on a solid Murashige and Skoog (MS) medium^29^ for 2 weeks at the temperature of 25–23 °C with a 16/8 h light/dark photoperiod. Then, the seedlings were transferred into the 10 × 7 cm glass culture plates that contained MS medium in order to achieve more growth. All the experiments were performed in accordance with relevant guidelines and regulations.

### PBI121–MAP30–KDEL expression vector

**Figure 1:**
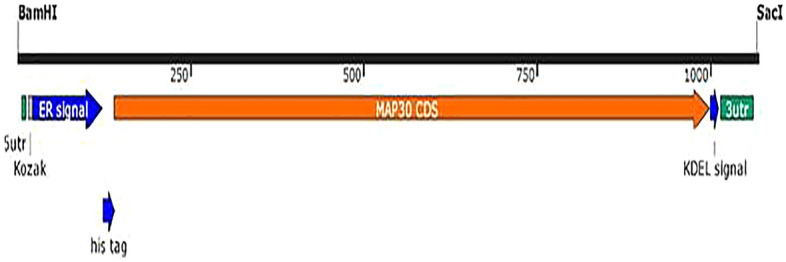
Expression construct of the recombinant MAP30 called *pBI121–MAP30–KDEL*. The *nptII* (kanamycin-resistance gene) under the control of the *nopaline* *synthase* (*NOS*) promoter and the codon-optimized *MAP30* CDS under the control of the *CaMV* *35S* promoter and *NOS* terminator were illustrated. The 6 × His tag and ER-retention signal KDEL were fused at N- and C-terminus in-frame with the *MAP30* CDS, respectively.

In order to produce MAP30 in hairy roots of *N.* *tabacum*, the coding region (CDS) containing 861 bp was designed and optimized based on the codon-usage bias of the host^19^ (Biomatik, Canada). After inserting *MAP30* CDS into the pBI121 expression vector through *Bam*HI and *Sac*I sites, a recombinant *pBI121–MAP30–KDEL* expression vector was designed. In this vector, which contained ampicillin and kanamycin selectable marker genes, *MAP30* expression was controlled by CaMV 35S promoter and nopaline synthase (*NOS*) terminator. In addition, a 6 × His tag and ER-retention signal KDEL were fused at the N- and C-terminus, respectively, in-frame with the *MAP30* CDS (Fig. 1). The ER retention signal is used to target the rMAP30 into the ER lumen^19^. The yield of protein expression, and the accumulation level will be two to tenfold more greater than that of proteins secreted into the culture medium^30,31^. In addition, Moghadam et al., 2016 confirmed, rMAP30-KDEL preserved its native biological properties, which were virus topological-inactivation and antimicrobial activities^19^.

### Transformation of *E. coli*

Two microliter of a 10 times diluted synthetic pBI121-MAP30-KDEL expression vector (30 ng/μL) was used to transform *E.* *coli* strain DH5α through the electroporation method. To do the electroporation, we used a 2 mm cuvette with resistance 200 Ω and capacitance 25 µF at 2500 V. Then, the transfected bacteria were dispersed on the 50 mg/L kanamycin-supplemented Luria–Bertani (LB) agar medium and became incubated at the temperature of 37 °C overnight. Single colonies were selected and cultured in a 50 mg/L kanamycin-supplemented liquid LB medium with agitation at 37 °C overnight. The process of transformed colonies confirmation was carried out through PCR and digestion of the extracted plasmid.

### Transformation of *Agrobacterium rhizogenes*

The expression plasmid was extracted from transformed *E.* *coli* using a plasmid extraction kit (ViVANTIS, Selangor Darul Ehsan, Malaysia). The integrity and quantity of plasmid were evaluated by the visual observation of plasmid bands on a 1% agarose gel. Then, 1 μg of the pBI121-MAP30-KDEL plasmid was digested as a result of the reaction of *Sac*I with a 20 μL final volume for 2 h at 37 °C. In order to transfer the expression vector, competent cells of the *A.* *rhizogenes* strain ATCC AR15834 were first prepared using 0.1 M calcium chloride. Then, the confirmed pBI121-MAP30-KDEL plasmid was used to transform 100 μL of *A.* *rhizogenes* using the freeze–thaw method. Moreover, 1 mL of liquid LB was added and the cells became incubated at 28 °C in a dark medium for 2 h. The transformed bacteria were dispersed on the kanamycin- and rifampicin-contained (100 mg/L) LB agar medium and then, became incubated at 28 °C in the darkness for 48 h. Transformed colonies were confirmed as a result of the extracted plasmid digestion and PCR.

### Production of transgenic hairy roots

**Figure 2:**
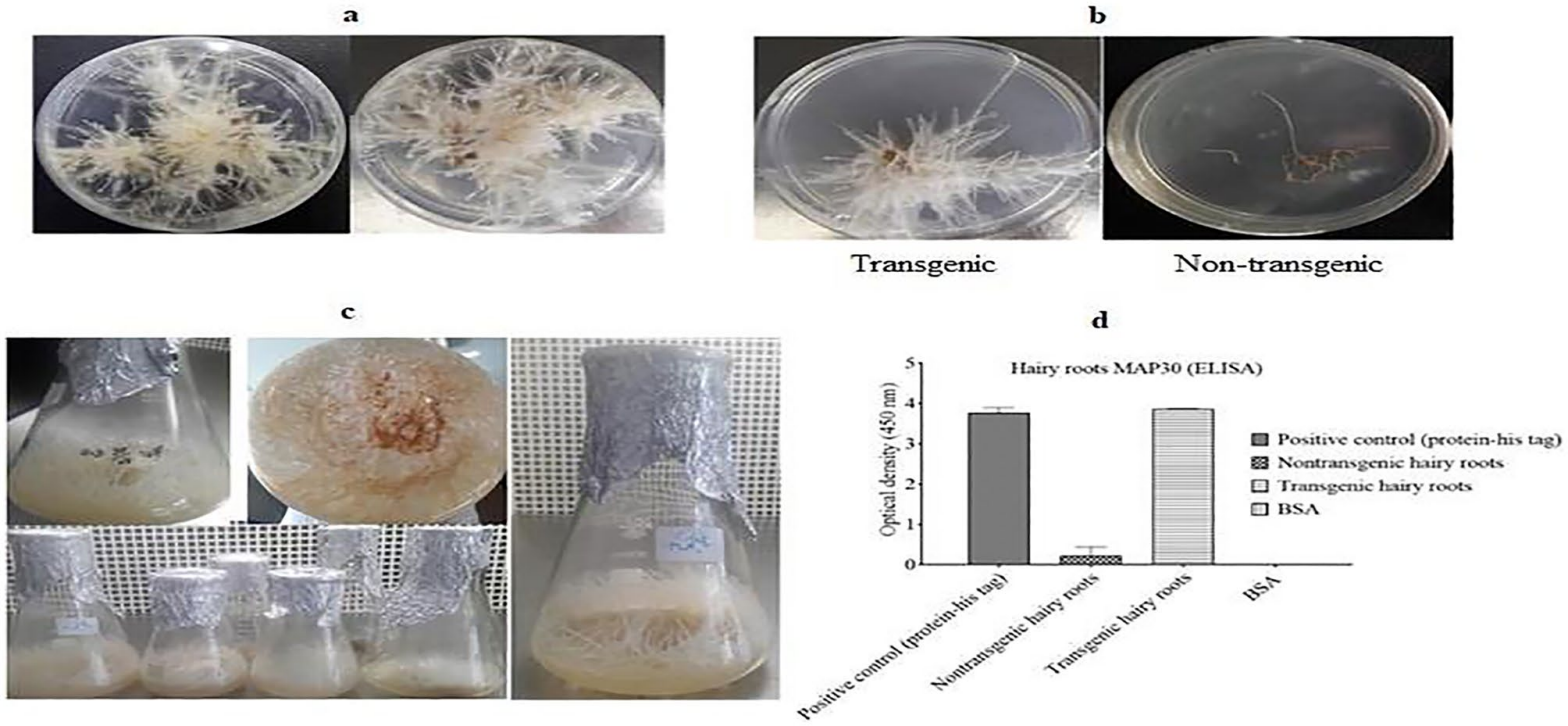
Formation and elongation of *N.* *tobacco* hairy roots at different periods after the *A.* *rhizogenes* infection. (**a**) The emergence of transgenic hairy roots from bacterial inoculated leaf explants grown on a solid MS medium with 30 mg/L of meropenem and 100 mg/L of kanamycin, which had been maintained at 25 °C under a 16/8 h light/dark photoperiod for 2 weeks after the infection; (**b**) the growth pattern of non-transgenic hairy root samples on kanamycin, and non-kanamycin culture media. The inability to grow on a medium that contains kanamycin and meropenem, and the growth process on an environment that only contains meropenem indicates that they are not transgenic; therefore, they have not received the expression construct (Mer indicates meropenem antibiotic, and Kan indicates kanamycin antibiotic). (**c**) The growth of hairy roots cultivated in a 250-mL Erlenmeyer flask that contains MS liquid medium, which is refreshed weekly and only contains meropenem antibiotic at 28 °C in the darkness with gentle shaking for 1 or 2 months. (**d**) Confirmation of the production of rMAP30 in the hairy roots of tobacco plant tissues using ELISA, BSA indicates bovine serum albumin.

To produce the hairy roots, 1 cm pieces of tobacco leaves were inoculated in the transformed *A.* *rhizogenes* liquid medium for five minutes. The leaves were kept on the MS medium for three days in the darkness at 25 °C and then, the explants became transferred to a fresh MS medium that was supplemented with 30 mg/L of meropenem and 100 mg/L of kanamycin, and were maintained at 25 °C under a 16–8-h light/dark photoperiod for 2 weeks in order to initiate the hairy roots (Fig. 2a,b).

### Maintenance of hairy roots in media culture

In order to be sure that the transformed hairy roots were free from agrobacterium contamination, five subcultures were performed, followed by transfer into a 250 mL Erlenmeyer flask with liquid MS medium. The samples became incubated at 28 °C in the darkness and then, they were gently shacked inside the shaker incubator for two months. The medium was refreshed weekly (Fig. 2c).

### Extraction of DNA and RNA and synthesis of cDNA

Genomic DNA was extracted using the modified CTAB method^32^. Total RNA was extracted using a Column RNA isolation kit (DENAzist, Mashhad, Iran). Then, the quantity and concentration of the RNA and DNA were measured using a Nanodrop device (Thermo Fisher Scientific, USA). Also, RNA integrity and quantity were evaluated through the visual observation of 28 S and 18 S rRNA bands on a 1% agarose gel. Then, cDNAs were synthesized using a firststrand cDNA synthesis kit (Thermo Fisher Scientific, Germany) according to the instructions provided by the manufacturer. Moreover, DNA-free total RNA (1 μg) was reverse transcribed using oligo-dT primers (Thermo Fisher Scientific, Germany), and cDNA samples were stored at −20 °C until they were applied.

### Confirmation of transgenic hairy roots

**Figure 3:**
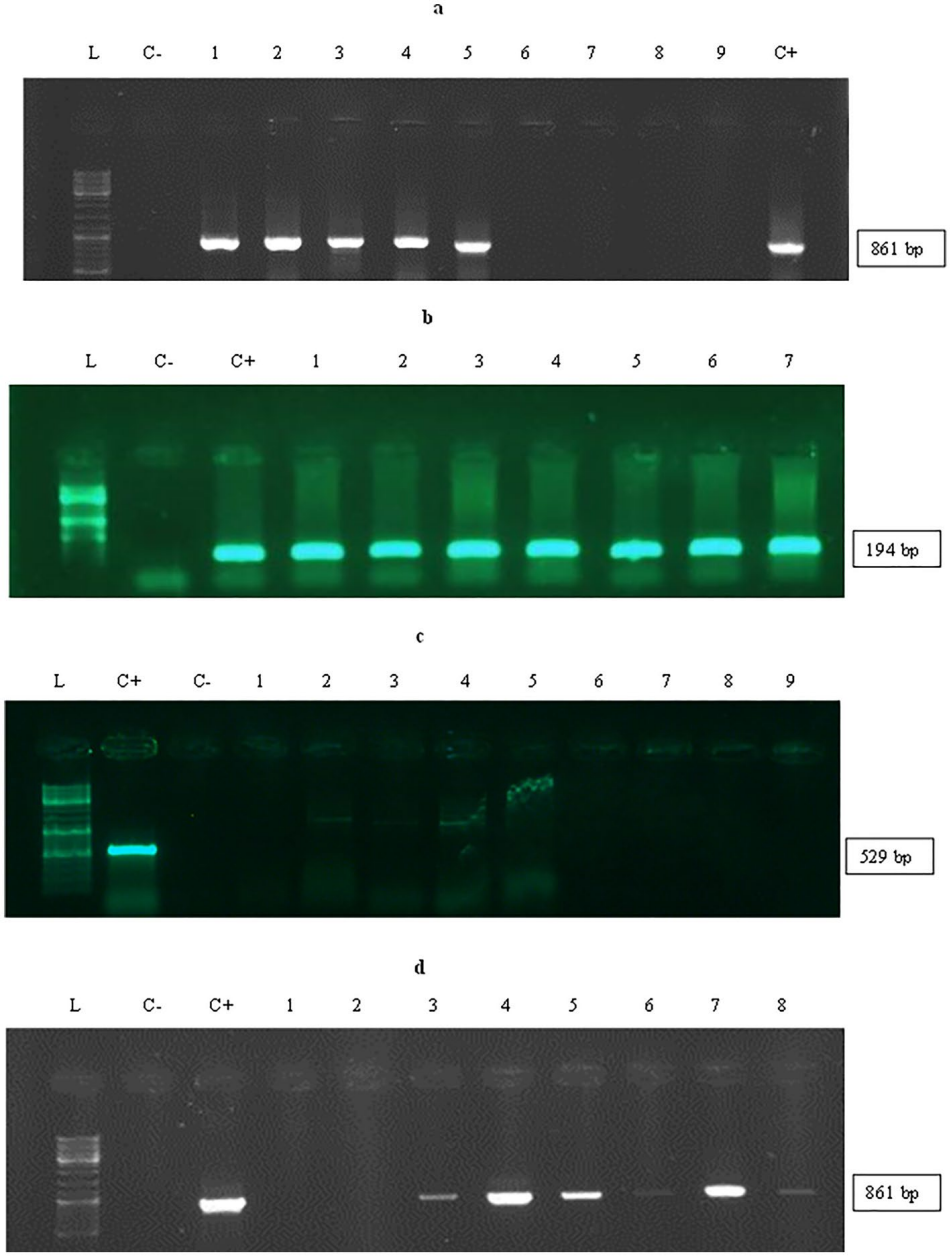
Confirmation of the transgenic lines. PCR amplification of *MAP30* (**a**) and *rolB* (**b**) using DNA template, and *MAP30* (**c**) using cDNA, template derived from *N.* *tobacco* hairy root lines. Lane L, Ruler 1-kb DNA ladder Mix (Thermo Fisher Scientific, Germany). (**a**) Lane 1: negative control (water), lanes 2–6: transgenic hairy roots samples, lane 7–10: non-transgenic hairy root samples, lane 11: positive control (transformed *A.* *rhizogenesis* DNA); (**b**) lane 1: negative control (water), lane 2: positive control (transformed *A.* *rhizogenesis* DNA), lanes 3–6: transgenic hairy roots samples, lane 7–9: non-transgenic hairy root samples; (**c**) lane 1: negative control (water), lane 2: positive control (transformed *A.* *rhizogenesis* DNA), lanes 3 and 4: non-transgenic hairy root samples, lane 5–10: transgenic hairy root samples.

Primers specific for recominant gene of *MAP30* and bacterial genes of *rolB* and *virG* were designed using Allele ID 7 (PREMIER Biosoft, USA) and Vector NTI 11 software (Thermo Fisher Scientific, USA) (Table 1) (Fig. 3)^19^. The *virG* amplification-specific primers were applied to confirm the elimination of *A.* *rhizogenes* infection (Fig. 3c). Then, *rolB* amplification-specific primers within the putative transgenic hairy roots were applied (Fig. 3b). Finally, the specific primers were used to amplify *MAP30* cDNA and DNA templates extracted from putative transgenic hairy root samples (Fig. 3a,d).

**Table 1:** Sequences of the primers applied for PCR-based characterization of transgenic hairy roots. Moghadam et al., 2016 designed primers using Vector NTI 11 and Allele ID 7 software19.

Primer name	Sequence	Ta (°C)	Product length (bp)
MAP30-F	ATGGCACCACAAAAGGAGAAC	58	861
MAP30-R	AACCTGAAACCTTTCTCCTGTAG		
RolB-F	AAGTGCTGAAGGAACAATC	54	194
RolB-R	CAAGTGAATGAACAAGGAAC		
VirG-F	CCTTGGGCGTCGTCATAC	55	529
VirG-F	TCGTCCTCGGTCGTTTCC		

*Ta* temperature annealing, *F* forward, *R* reverse.

### Protein extraction and purification

The process of protein extraction from confirmed transgenic hairy roots was carried out using 50 mM phosphate buffer (pH 7). At the first, 5 g of fresh and white hairy roots tissue were grounded under the liquid nitrogen, and the powder was suspended in the 1:1 phosphate buffer w/v and vortexed. Then, the supernatant was prepared through performing the centrifugation procedure at 13,000 rpm for 20 min at the temperature of 4 °C. Furthermore, the concentration of total protein was determined using Bradford method^33^. The proteins were stored at −20 °C, while protein purification was conducted under native conditions through applying a Ni–NTA spin column (cat. No. 31014, Qiagen). Briefly, the matrix column was washed with distilled water for five times. Then, 3 mL of the protein extraction buffer was added while the outlet was closed, and the column was held for half an hour at the room temperature. Buffer was removed, 2 mL of the protein was poured from the filtered filter paper over the pillow, and became stored for one night in the refrigerator. After the protein was withdrawn, columns were washed for 3–5 times with 2 mL of the washing buffer (Imidazole 20 mM, NaCl 300 mM, NaH_2_PO_4_ 50 mM with pH 8). Column output fractions were collected individually; also, 2 mL of the detergent buffer prepared with 300 mM NaCl, 50 mM NaH_2_PO_4_, 500 mM Imidazole, pH 8 was added to the column. This procedure was repeated 4–5 times.

### Confirmation of recombinant MAP30 by ELISA

To confirm the rMAP30, immunoblot technique was implemented with anti-his-tag antibody. Initially, 30 μg of the rMAP30 was transferred into each well of ELISA plate. The non-transgenic total protein and his tag protein, were respectively applied as the negative and positive controls. Furthermore, the absorbance at 450 nm wavelength was measured by ELISA reader (Fig. 2d).

### Anti-microbial activity assay

To confirm anti-microbial activity of rMAP30, three groups of microorganisms including gram-positive bacterial strain of *Streptococcus* *aureus* PTCC 1112 (ATCC 6538), gram-negative bacterial strain of *E.* *coli* PTCC 1330 (ATCC 8739), and fungal strain of *Candida* *albicans* PTCC 5027 (ATCC 10231) were examined^19^. For anti- microbial assay two protein samples were taken, T1 and T2 that indicate the transgenic total protein and rMAP30 respectively); moreover, T0 (Non-transgenic total protein) was considered as the control.

### Minimum inhibitory concentration assay

The process of determination of the Minimum Inhibitory Concentration (MIC) was carried out through applying a sequential dilutions method in 96 well plates. Furthermore, 180 μL of LB culture medium was transferred into all of the wells and then, 150 μL of protein was added to the first well and became mixed. At the next step, 180 μL of this solution was transferred into the second well. This procedure was carried out for the last well and finally, 180 μL was discarded. Then, 10 μL of the microbial suspension was added to each well and wells were observed after the incubation completion, the last well, which did not have the turbidity of the microbial growth, was introduced as the MIC. The MIC calculation was conducted as the following (600 nm absorbance for bacteria)^34^.

$${\text{Inhibition rate }}\left( \% \right) \, = \, \left( {{\text{OD}}_{{\text{positive control}}} - {\text{OD}}_{{{\text{sample}}}} } \right) \, \div \, \left( {{\text{OD}}_{{\text{positive control}}} - {\text{OD}}_{{\text{negative control}}} } \right) \, \times {1}00$$

### Concentration of extracted protein

The extracted protein from hairy roots were placed in an Eppendorf freeze dryer for 16 h or one day, then was dissolved in a 1 ml, 50 mM, pH 7 phosphate buffer. The samples were stored at the temperature of −20 °C.

### Extraction of bacteriophage

To extract the bacteriophage effective in the *E.* *coli* infection, 50 mL of agglomerated and degreased urban sewage derived from Marvdasht was maintained in the refrigerator at 4 °C for one day to settle the existing sediment. The sewage was centrifuged after a nocturnal period for 20 min at 6000 rpm. The supernatant was respectively filtered through 0.22-micron filters. Furthermore, 27 mL of the filtered sewage with 2 mL of 24-h *E.* *coli* culture and 5 mL of the liquid LB medium within the Erlenmeyer flask was incubated in order to achieve a better aerating and then, it was transferred into a shaker incubator at 37 °C for 72 h. Then, 3 mL chloroform was added to each shaker incubator. Samples were shacked at 160 rpm in a shaker for 15 min at the room temperature and then, became centrifuged at 4 °C for 30 min at 3500 rpm. The last steps were repeated three times. After the last centrifugation stage, the supernatant was filtered through a 0.22-micron filter and stored at 4 °C in the darkness. Optical absorbance was measured at a wavelength of 600 nm^35^. The bacteriophage concentrations applied in the current study were as the following: 171.4 ng/µL, diluted 1:10 equal to 23.0 ng/µL, and diluted 1:20 equal to 14.7 ng/µL, respectively.

### Bacteriophage host

**Figure 4:**
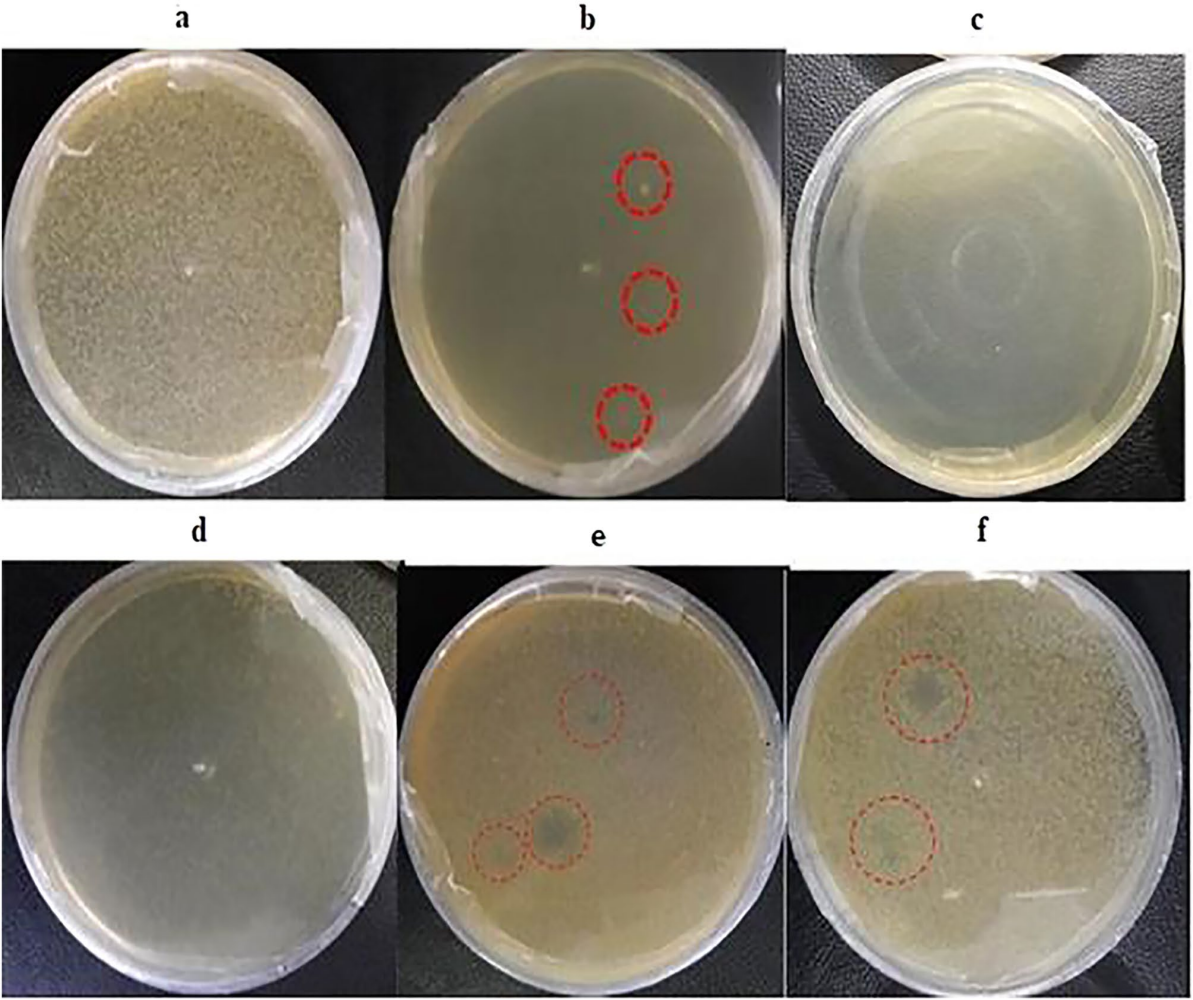
The effect of bacteriophage amount on the growth inhibition of *E.* *coli*. (**a**) The absence of bacteriophage in the culture medium. (**b**) The *E.* *coli* plus 200 μL of bacteriophage in the culture media and the growth of only 2–3 bacterial colonies in the entire medium. (**c**) Use of a maximum of 2000 μL of bacteriophage in the culture medium without the bacterial growth. (**d**) The bacterial culture with 5 μL of bacteriophage in the medium and bactericidal inhibition growth. (**e**) The presence of bacteriophage in the medium as a result of reduction of the number of centrifuges into two turns, and plaque formation in the culture medium. (**f**) The use of concentrated bacteriophage and double centrifuge, and plaque appearance.

The following assays were performed with the purpose of confirming the bacteriophage presence in the solution achieved during the extraction process against *E.* *coli* as the host, and confirmation of its effect on the growth of bacteria. To achieve this purpose, a fresh culture of *E.* *coli* was first prepared with an optical absorbance of 0.5 (OD_600nm_ = 0.5). Then, a 50 mL solid 40–45 °C LB medium, which was still liquid, became mixed with 30 μL of the bacterial culture. In addition, various amounts of undiluted phage solution (5, 10, 20, 30, 50, 100, 200, 300, 500, 1000 and 2000 μL) and, 100 μL 1:10 and 1:20 diluted bacteriophage respectively were added to the mixture (Fig. 4). The achieved mixture was then transferred into petri dishes. The test controls were as the following: 30 μL of bacteria added to 50 mL semi solid LB medium as the positive control to emphasize the bacterial growth potential, and 50 mL of semi solid LB medium plus 1000 μL phage-contained solution as the negative control to emphasize the phage growth inability of LB medium. Samples were kept in an incubator for 24 h at 37 °C, and the bacterial growth was studied after this period.

### Anti-bacteriophage assay

Various experiments were designed based on MIC experiment to investigate the inhibitory effect of transgenic total protein. In the first assay, which named the pre-treatment of 15 μg μL transgenic total protein and 10 μL of bacteriophage (1:20 and 1:10 diluted bacteriophage), as the following: bacteriophage and transgenic total protein were first treated at 37 °C for two different periods of time 3 and 24 h and then, 30 μL of fresh *E.* *coli* medium (OD_600nm_ = 0.5) was added to the 50 ml semi-solid LB medium for each mixture. Controls were as the following: (A) bacteriophage plus protein extraction buffer only; (B) non-transgenic total protein only; (C) non-transgenic total protein plus bacteriophage agent; (D) transgenic total protein (Table 2).

**Table 2:** Anti-bacteriophage activity of MAP30.

Assay type	Transgenic total protein (μg)	Bacteriophage (μL)	*Escherichia* *coli* (μL)	Liquid LB medium (μL)	Incubation time (h)
Pre-treatment	15	10 (1:10 and 1:20 diluted)	–	–	3
Pre-treatment	15	10 (1:10 and 1:20 diluted)	–	–	24
Simultaneous	15	10 (1:20 diluted)	30	200	3
Simultaneous	15	10 (1:20 diluted)	30	200	24

The pre-treatment and simultaneous assays was conducted in two periods of the time using transgenic total protein and bacteriophage in different dilutions (1:10 and 1:20).

The second assay, was carried through the simultaneous assay15 μg of transgenic total protein, 200 μL of the liquid LB medium, 10 μL of 1:20 diluted bacteriophage, 30 μL of fresh *E.* *coli* medium (OD_600nm_ = 0.5) were mixed and pipetted perfectly. The micro tubes were incubated at 37 °C for two different periods of time, 3 and 24 h. The mixtures were added to 50 mL of 40 °C semi-solid LB medium, then mixtures divided into two petri dishes and incubated at 37 °C for 24 h. Controls were as the following: (A) bacteriophage plus *E.* *coli* only; (B) non-transgenic total protein plus *E.* *coli* only; (C) non-transgenic total protein plus *E.* *coli* plus bacteriophage agent; (D) transgenic total protein plus *E.* *coli* only (Table 2).

The third assay was conducted in order to investigate the effects of various parameters on the bacterial growth, which can be based on the bacteriophage preparation procedure. As a result, the volume of wastewater and bacteria were considered to be equal and the treatment period decreased from 72 to 24 h; also, the number of final centrifuges of the suspension reduced from 3 times to 2 and 1 times, repeatedly. The controls were similar to those mentioned above. Four replications for each test were considered.

### Growth conditions of host plants

Seeds of *Chenopodium* *quinoa* and *Nicotiana* *glutinosa* were achieved from the Center of Plant Virology Research, College of agriculture, Shiraz University. The seeds of *N.* *glutinosa* were cultivated in the greenhouse conditions. After 2–3 months, each plant was transferred into the separated rich soil-contained pots, and did not get inoculated until 3–4 leaf stage. Seeds of *C.* *quinoa* were also cultivated in the greenhouse conditions. After almost 1.5 to 2 months, each seeding was transferred into the rich soil-contained pots. Furthermore, the inoculation process did not occur until the 6–7 leaf stage .

### Purification of TMV and CMV

Both TMV and CMV were achieved from the Center of Plant Virology Research, College of Agriculture, Shiraz University. We confirmed these viruses using the specific primers of coat proteins in PCR. To purify the TMV, a modified protocol was applied^36^. The process of virus concentration measurement was carried out through applying a Nano-drop device (Thermo Fisher Scientific, USA) at a 260 nm wavelength. Moreover, CMV purification was conducted according to the modified protocol^37^. It is noteworthy that the virus concentration measurement was carried out at a 260 nm wavelength after calculating the related approximate weight using C = OD/E formula. Additionally, a CMV-containing plant extract was prepared by adding 12.5 to 14 times the leaves weight to 50 mM phosphate buffer and mixing it sequentially. Then, the achieved mixture was centrifuged at 12,000 rpm for 10 min. The supernatant, or viral extract, is taken and kept on ice until it is incubated.

### Anti-TMV and anti-CMV assays

There is an interaction between the transgenic protein and viruses, which would appear as a local lesion^38^. The achieved results were examined by a statistical analysis of T-test and (1 − T/C) × 100 formula (T indicates the treatment, C indicates the control) and then, became processed in the statistical charts using Minitab and Graph Pad Prism software. The anti-CMV and anti-TMV activities of rMAP30 were performed using two symmetrical leaves of *C.* *quinoa* and a single leaf of *N.* *glutinosa*, respectively. The assays include: 1. serially diluted transgenic total protein, and rMAP30 (including: undiluted, 1/2 diluted, and 1/4 diluted protein by a 10 mM protein extraction buffer), the purified or extraction-infected CMV and TMV, 2. The non-transgenic total protein or rMAP30 and purified or extraction-infected CMV or TMV, as well as the extraction buffer and purified or extraction-infected CMV or TMV. Based on the each assay condition two basic controls (first: non-transgenic total protein plus viruses, second: viruses plus extraction buffer) were considered. The suspensions were poured on the surface of the leaves, and spread by moving the index finger unilaterally and maintaining the same pressure for 7 times. Samples were stored in the greenhouse conditions for 5–7 days. To improve permeability of recombinant proteins or viruses, we used 0.1% DMSO in the extraction buffer. The amount and type of the extracted protein and virus used in each assay are provided in the tables (Tables 3, 4). Furthermore, details of designed assays are showed as the following.

**Table 3:** Anti-TMV activity of MAP30.

Simultaneous assay			
Protein	Volume (μg)	TMV	Volume (μg)
Undiluted rMAP30	0.6	Purified	0.1
1/2 diluted rMAP30	0.3	Purified	0.1
1/4 diluted rMAP30	0.15	Purified	0.1
Undiluted transgenic total protein	3	Purified	0.1
1/2 diluted transgenic total protein	1.5	Purified	0.1
1/4 diluted transgenic total protein	0.75	Purified	0.1

Time interval assay			
Protein	Volume (μg)	TMV	Volume (μg)
rMAP30	1	Purified	0.1

The different concentrations of purified recombinant MAP30 and the total protein extracted from transgenic lines are shown. Simultaneous assay indicates the treatments using MAP30 and TMV at the same time. Time interval assay indicates the treatments using MAP30 and TMV separately and in a period of the time.

**Table 4:** Anti-CMV activity of MAP30.

Simultaneous assay			
Protein	Volume (μg)	CMV	Volume (μg)
Undiluted rMAP30	0.6	Purified	0.1 (μg)
Undiluted rMAP30	0.6	Extraction-infected virus^a^	15 (μL)
1/2 diluted rMAP30	0.3	Extraction-infected virus	15 (μL)
1/4 diluted rMAP30	0.15	Extraction-infected virus	15 (μL)

Time interval assay			
Protein	Volume (μg)	CMV	Volume (μg)
rMAP30	1	Purified	0.1

The different concentrations of purified recombinant MAP30 and the total protein extracted from transgenic lines are shown. Simultaneous assay indicates the treatments using MAP30 and CMV at the same time. Time interval assay indicates the treatments using MAP30 and CMV separately and in a period of the time.

^a^This means the total extraction was obtained from virus infected leaves of the host.

### Simultaneous assay to antivirus activity

The experiments were performed as the following: (1) inoculation of leaves with the purified TMV plus three dilutions of transgenic total protein (Table 3); (2) inoculation of leaves with the purified TMV plus three dilutions of rMAP30 (Table 3); (3) inoculation of leaves with extraction-infected CMV plus three dilutions of rMAP30; (4) inoculation of leaves with the purified CMV plus three dilutions of rMAP30 (Table 4). The same conditions considered for controls. It should be noted that 16 replicates were considered for each experiment.

### Time interval assay to antivirus activity

The experiments were carried out as the following: (1) inoculation of leaves with purified TMV for 6 h then application of rMAP30, (2) inoculation of leaves with rMAP30 for 6 h then application of purified TMV (Table 4), (3) inoculation of leaves with purified CMV for 6 h then application of rMAP30, (4) inoculation of leaves with rMAP30 for 6 h then application of purified CMV (Table 4). The same conditions considered for controls. There were eight replicates for each test.

### Statistical analysis

Analysis of variance followed by Duncan's multiple range test was performed using MINITAB (Minitab, Inc., Pennsylvania, USA). In all cases, differences were regarded to be statistically significant at *P-value* ≤ 0.05 level. All experiments were performed in triplicate, illustrated using the GraphPad Prism software (GraphPad, USA).

## Results and discussion

### Confirmation of anti-bacterial activity of recombinant MAP30

**Figure 5:**
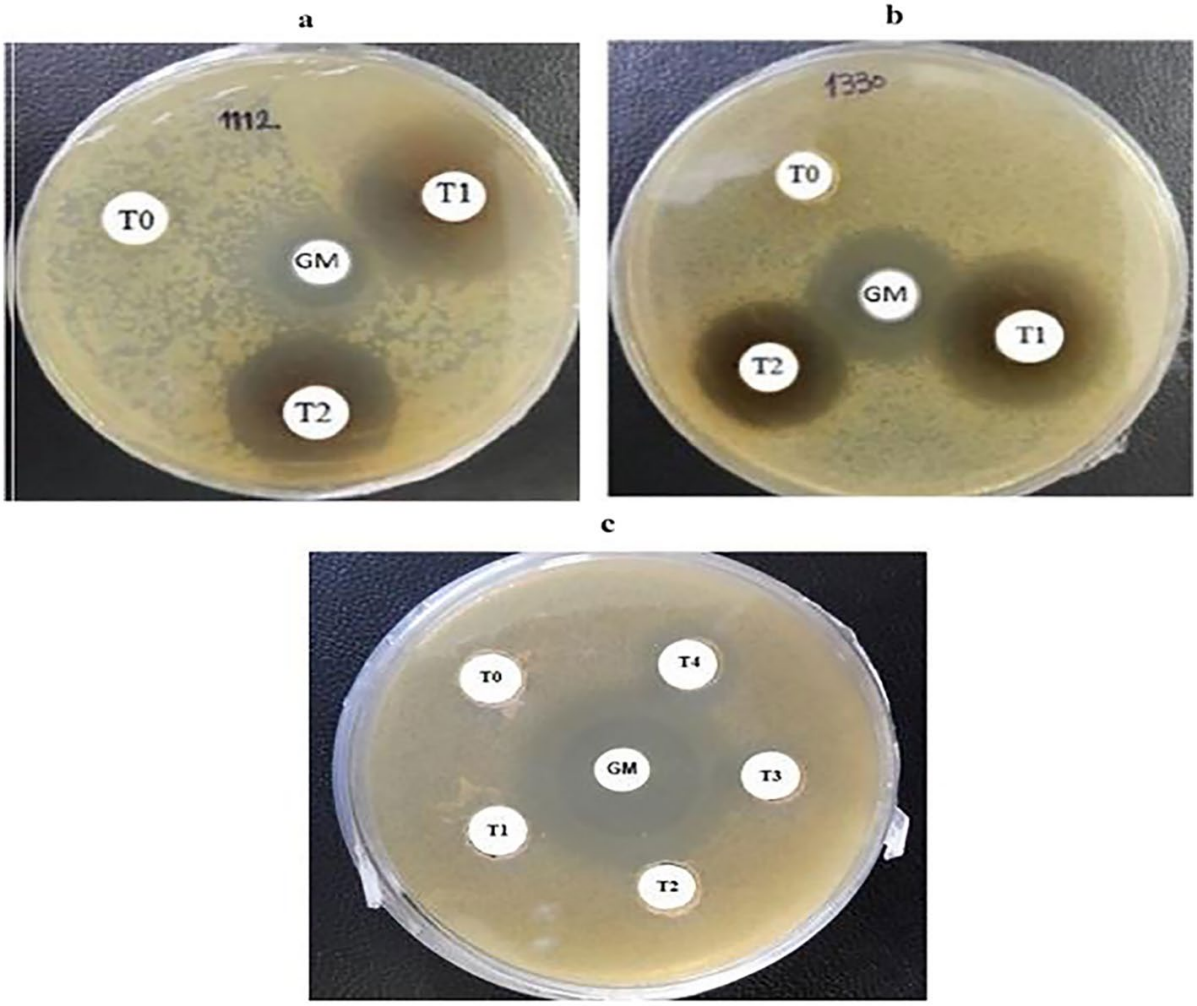
The anti-microbial and anti-fungal activities of *rMAP30-KDEL*. The anti-microbial activities of MAP30 extracted from the transgenic hairy root lines were assessed by *S.* *aureus* 1112 (**a**) and *E.* *coli* 1330 (**b**) through disc diffusion assay for 16 h (Table 5). GM, T0, T1 and T2 respectively indicate gentamycin (10 μg/disc), 75 μg of transgenic total protein (control), the transgenic total protein (55 μg), and the rMAP30 (10 μg). *C.* *albicans* 5027 (**c**); GM, T0, T1, T2, T3, and T4 indicate gentamycin (10 μg/disc), non-transgenic total protein (control, 75 μg/disc), and transgenic total protein (25, 40, 55, and 75 μg/disc respectively).

The confirmation of rMAP30 activity against microorganisms was carried out through experiments using total protein and non-concentrated rMAP30 using applying a sequential dilutions disk diffusion assay (Fig. 5). The necessity of this experiment was MIC determination. Table 5 represents the amount and type of extracted proteins for the anti-microbial assays. The derived proteins from the transgenic hairy roots—led to the inhibition zone formation (Fig. 5). Moreover, the diameter of the inhibitory zone was enhanced by the purified rMAP30 (Fig. 5a,b). The achieved results were complementary to the previous studies^19,21,39^. Generally, MAP30 is considered as a valuable alternative to anti-viral, anti-tumor, and anti-microbial drugs^21^.

**Table 5:** The anti-microbial properties of recombinant MAP30 against three microorganisms using disk diffusion method. The number of positive signs indicates the intensity of the activity.

MAP30 (μg)	*Escherichia* *coli*	*Streptococcus* *aureus*	*Candida* *albicans*
10	** + **	** + **	** + **
25	** ++ **	** ++ **	** ++ **
40	** +++ **	** +++ **	** +++ **
55	** ++++ **	** ++++ **	** ++++ **
75	** +++++ **	** +++++ **	** +++++ **

### Anti-bacteriophage activity of recombinant MAP30

**Figure 6:**
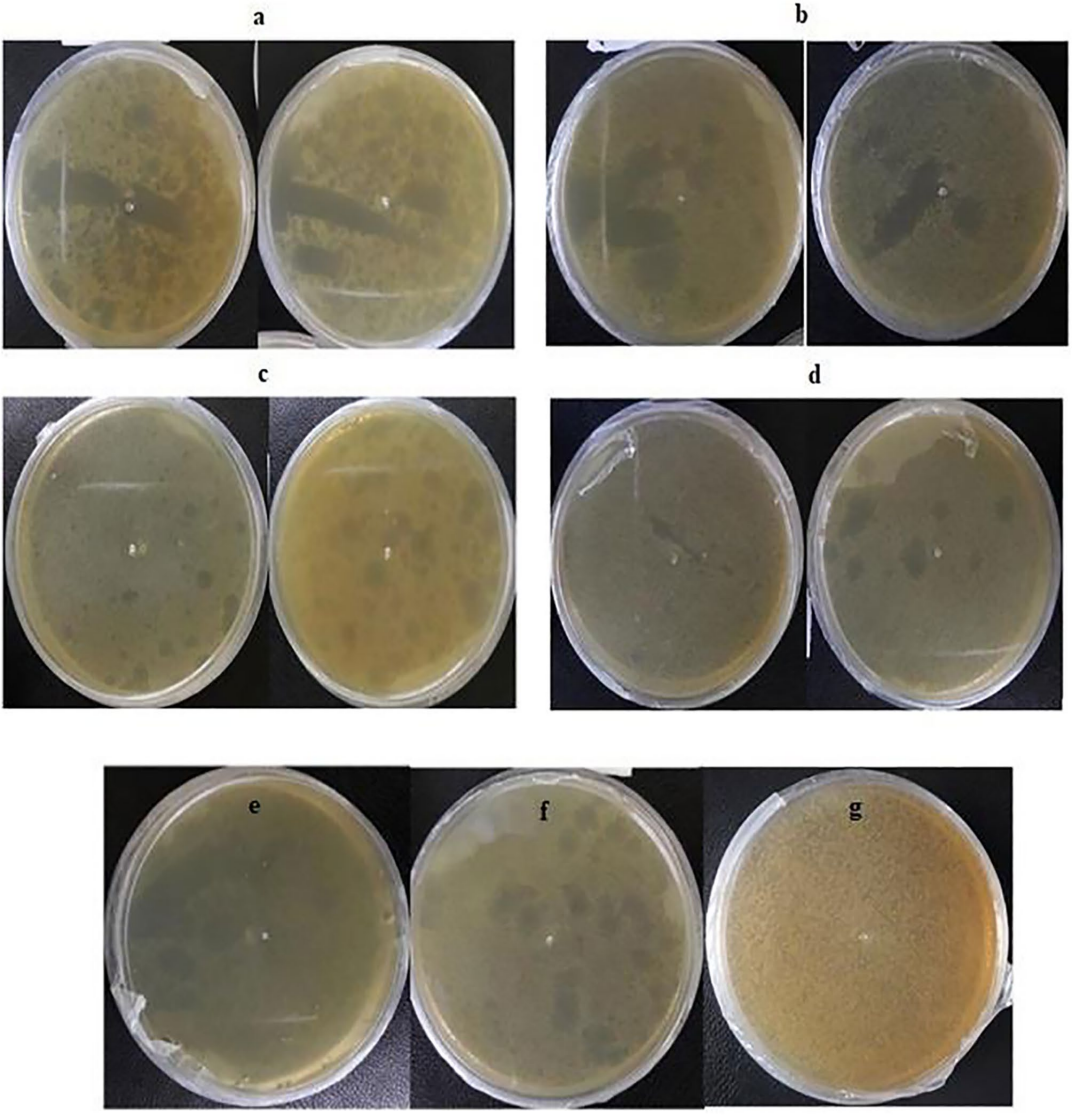
The inhibition of transgenic total protein on bacteriophage, during its pre-treatment with 1:10 and 1:20 dilution of bacteriophage. 2 out of 4 replications are shown for each test (Table 2). (**a**) Pre-treatment of 1:10 diluted bacteriophage with transgenic total protein for 3 h at 37 °C, and consequently adding 30 μL of *E.* *coli*. then the symptom observation after 24 h. (**b**) Pre-treatment of 1:10 diluted bacteriophage with transgenic total protein for 24 h at 37 °C, and consequently adding of 30 μL of *E.* *coli* , then the symptom observation after 24 h. (**c**) Pre-treatment of 1:20 diluted bacteriophage with transgenic total protein for 3 h at 37 °C, and consequently adding 30 μL of *E.* *coli* ,then the symptom observation after 24 h. (**d**) Pre-treatment of 1:20 diluted bacteriophage with transgenic total protein for 24 h at 37 °C, and consequently adding 30 μL of *E.* *coli* ,then the symptom observation after 24 h. (**e**) 1:20 diluted bacteriophage, without protein, added to the bacterial environment (negative control). (**f**) Pre-treatment of 1:20 diluted bacteriophage with non-transgenic total protein for 24 h at 37 °C, and consequently adding 30 μL of *E.* *coli*, then the symptom observation after 24 h (the 3-h pre-treatment was conducted with the same results as (negative control). (**g**) *E.* *coli* growth’s pattern (negative control).

The 1:10 and 1:20 diluted bacteriophages normally inhibited the growth of *E.* *coli*. However, when the transgenic total protein of MAP30 was added to the culture medium the plaques were observed, which indicate the inhibitory effect of MAP30 on phage infection. In order to investigate more, experiments were designed. The anti-phage activity of transgenic total protein in the simultaneous and pre-treatment assays with the bacteriophage dilutions of 1:10 and 1:20 was confirmed in two time interval (3 and 24 h) (Fig. 6) (Table 2). In the pre-treatment assay, after incubation 10 μL of phage and 15 μg of total transgenic protein for 3 h, the bacterial growth rate increased and the plaque numbers decreased. (Fig. 6a,c) (Table 2). After bacteriophages were treated for a longer period of time (24 h), the growth pattern of the bacteria increased, and plaque size and number were limited (Fig. 6b,d) (Table 2). It is noteworthy to mention that more scatter and larger plaques observed when the 1:10 dilution is used compared to the 1:120 dilution of phage (Fig. 6).

Apparently, the transgenic total protein inactivates the phage by altering its topology, therefore it will not be able to attack bacteria effectively. The two main controls include: (1) using only phage and buffer, and (2) non-transgenic total protein plus phage, confirmed the accuracy of the results (Fig. 6e,f). In the all controls, the normal growth pattern of *E.* *coli* was observed (Fig. 6g). In the simultaneous assay, the co-treatment of 10 μL of 1:20 diluted phage, 30 μL bacteria and the 15 μg transgenic total protein for 3 or 24 h (Table 2). for each test the plaques and bacterial colonies were monitored after 24 h of the co-treatment showed the formation of smaller bacterial colonies compared with 3 h. The intensified results were observed in 24 h treatment. The results accuracy was confirmed by the controls, while the results of protein effect control were confirmed.

The inhibitory effect was again observed in the simultaneous assay, despite the fact that the control intensity was slightly less than pre-treatment assays. Due to the fact that the anti-phage activity of recombinant MAP30 have not been previously studied, the results of current study could be compared with those of previous investigations that have studied antiviral properties, such as anti-HIV activity through RNase irreversible activity^40^. In fact, MAP30 inhibits both the infection and proliferation of HIV due to RNase activity^41,42^. Genomic DNA degradation can even occur in the single-stranded DNA^20^. Generally, it is found that recombinant MAP30 is similar to wild MAP30 in the topologically inactivation of viral DNA, inhibition of viral DNA fusion, and inactivation of cell-free ribosomes^22^. The supercoiled double-stranded DNA plasmid was nicked and the circular topology was converted to the linear form after incubation with recombinant MAP30 (0.1 µg/µL) for 2 h, in which demonstrated recombinant MAP30 exhibited DNase-like activity^15,19^. All of these studies help us to achieve a better understanding about MAP30 functions on the bacteriophages.

### Anti-TMV activity of rMAP30

**Figure 7:**
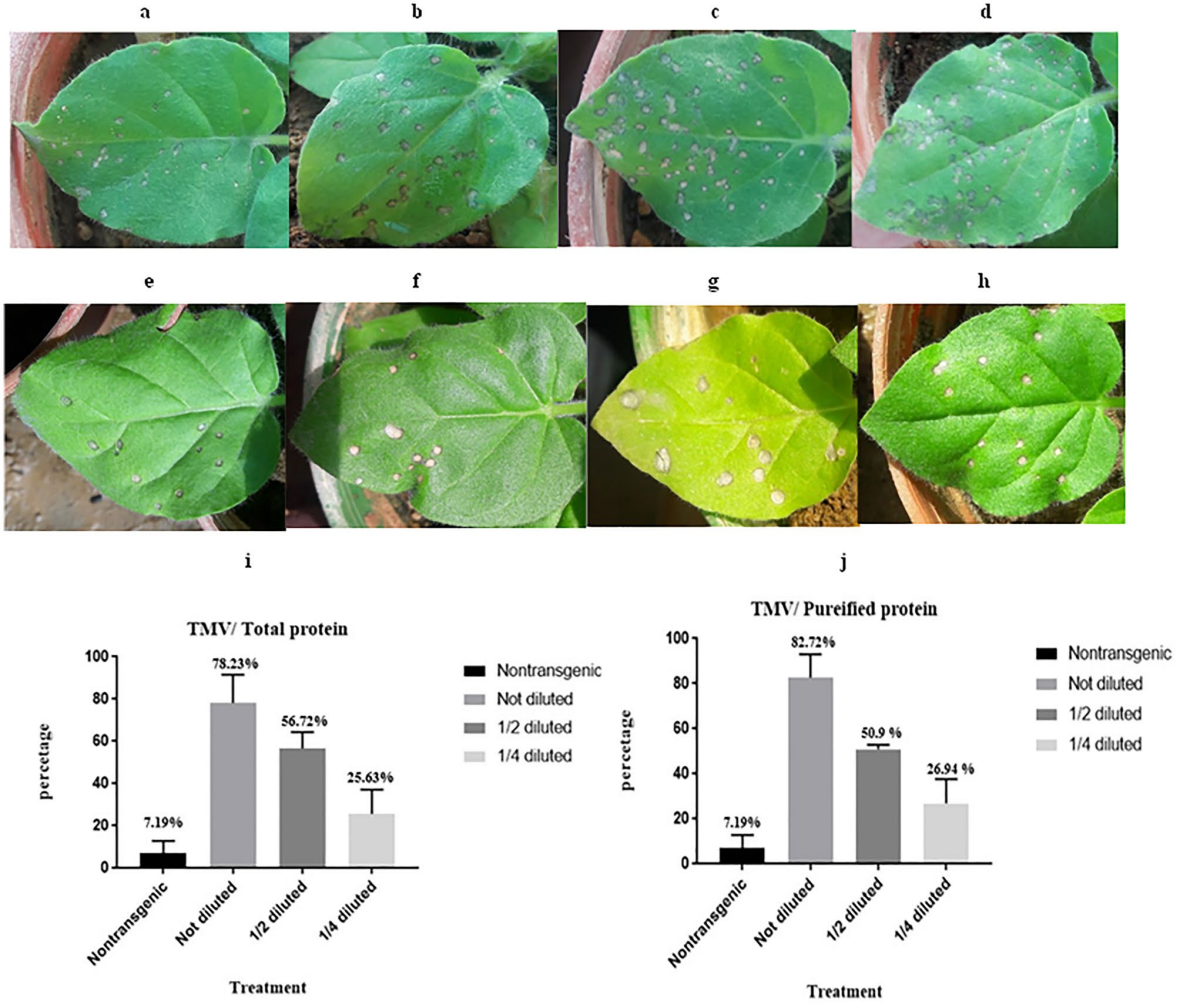
The simultaneous inoculation of *N.* *glutinosa* seedling with purified TMV and transgenic total protein in three dilutions, and purified TMV plus rMAP30 in three dilutions. Purified TMV plus non-transgenic total protein (negative control) (Table 3). There were 16 replicates for each test. **(a)** Test conducted with undiluted transgenic total protein. **(b)** Test conducted with one second diluted transgenic total protein. **(c)** Test conducted with one-quarter dilution of transgenic total protein. **(d)** Test conducted with non-transgenic total protein in undiluted state. **(e)** Test conducted with undiluted rMAP30. **(f)** Test conducted with one second diluted rMAP30. **(g)** Test conducted with one-quarter dilution r MAP30. **(h)** Test conducted with non-transgenic total protein in undiluted state. D4 indicates the day of symptoms observation. The virus and buffer existed in the right half of each leaf, as well as the protein and virus within the left half of each leaf were inoculated. **(i,j)** The viral infection controlling graph for rMAP30 in different dilutions versus non-transgenic protein. The highest control percentage was observed when the undiluted rMAP30.

The anti-TMV activity of recombinant MAP30 was confirmed through the symptoms intensity on the *N.* *glutinosa* inoculated leaves. The simultaneous assay was carried out using inoculation of leaves with purified TMV plus transgenic total protein or rMAP30 in different dilutions (undiluted, 1/2 diluted, and 1/4 diluted protein) (Table 3). The all dilutions of transgenic total protein and rMAP30 showed positive control effect on TMV. The less protein was diluted, the more significant reduction in the number of infection spots on the leaves was observed. As one of the controls during the tests, in order to show that the extraction buffer did not have an antiviral effect, purified TMV virus inoculation with the buffer was used on the leaves. The result was similar when the virus caused infection on the leaf alone (Fig. 7).

**Figure 8:**
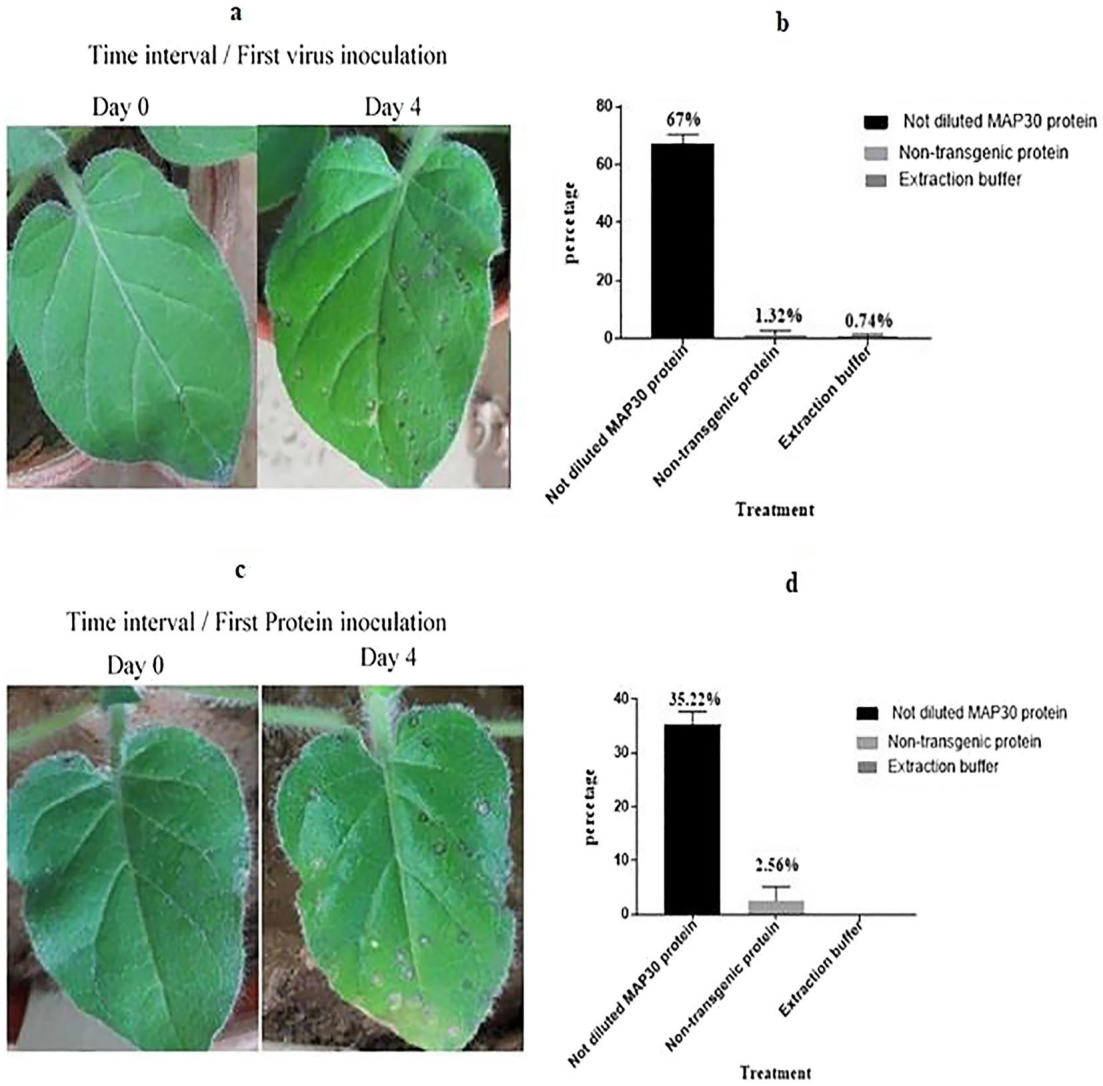
The inoculation of *N.* *glutinosa* seedling in the two-time interval assay with TMV and rMAP30 (Table 3). There were 8 replicates for each test**. (a,b)** The primary incubation with viral infection for a 6 h, then apply rMAP30 on the leaves surface. **(c,d)** The primary incubation of rMAP30 for 6 h, then apply viral infection on the leaves surface; D0 and D4, and D7 respectively indicate the day of applying the treatment either day, 4, and 7 days after the treatment It has been shown that the higher infection control observed when viral incubation have done 6 h prior than rMAP30. However, the control percentage in the initial treatment assay with rMAP30 is also significant and the number of viral spots is reduced.

The results achieved from the simultaneous assays were processed in the statistical charts using Graph Pad Prism software after counting spots and then, became placed in the (1 − T/C) × 100 formula. Generally, it was confirmed that the controlling effects of rMAP30 and transgenic total protein at the undiluted, 1/2 diluted, and 1/4 diluted states were determined to be 82.87% and 78.23%, 50.9% and 56.72%, and 26.94% and 25.63% respectively (Fig. 7i,j). The results showed that the most viral spot reduction in the time interval assay was found to be almost 67%, which is related to the rMAP30 inoculation 6 h after the virus infection, while the least amount was approximately 35.22%, in pre-virus incubation, which remarkably is almost higher than the viral infection control percentage in the simultaneous assay conduct by 1/4 diluted protein (Fig. 8). All of the results were self-certified for the TMV inhibitory activity of rMAP30 and transgenic total protein.

### Anti-CMV activity of transgenic total protein and MAP30

**Figure 9:**
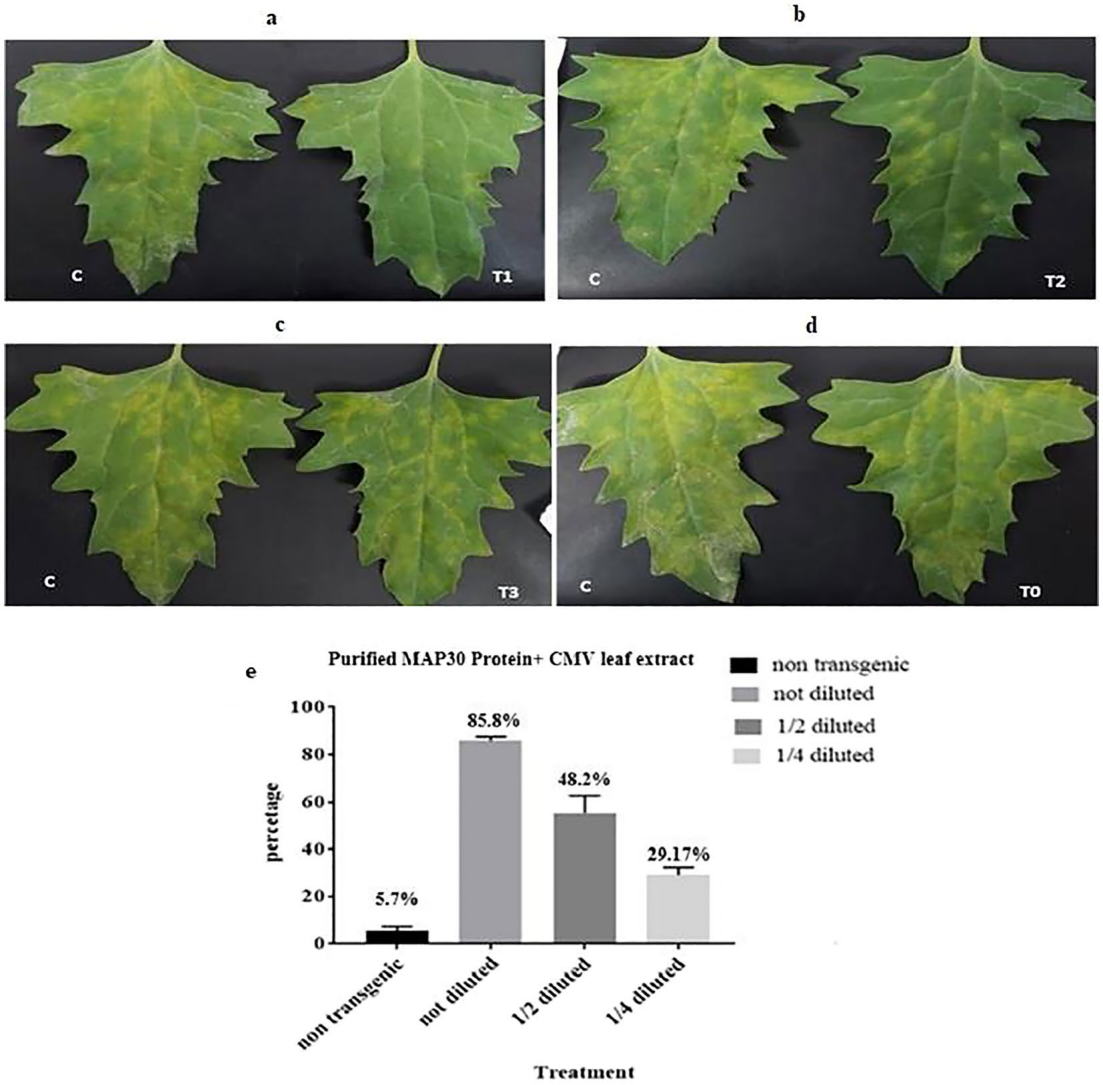
The simultaneous inoculation of *C.* *quinoa* seedlings Incubation the extraction-infected CMV and rMAP30 in three dilutions. Incubation with non-transgenic lines used as control (Table 4). There were 16 replicates for each assay.** (a)** The inoculation with undiluted rMAP30. **(b)** The inoculation with one second diluted rMAP30. **(c)** The inoculation with one quarter diluted rMAP30. **(d)** The inoculation with non-transgenic protein (T1, T2, T3, T0, and C respectively) indicate the treatment by undiluted, one second diluted, one quarter diluted rMAP30, non-transgenic protein, and CMV). **(e)** Viral infection intensity graph for rMAP30 in different dilutions versus non-transgenic protein. The results shows that the highest control percentage was observed when the rMAP30 was not diluted.

**Figure 10:**
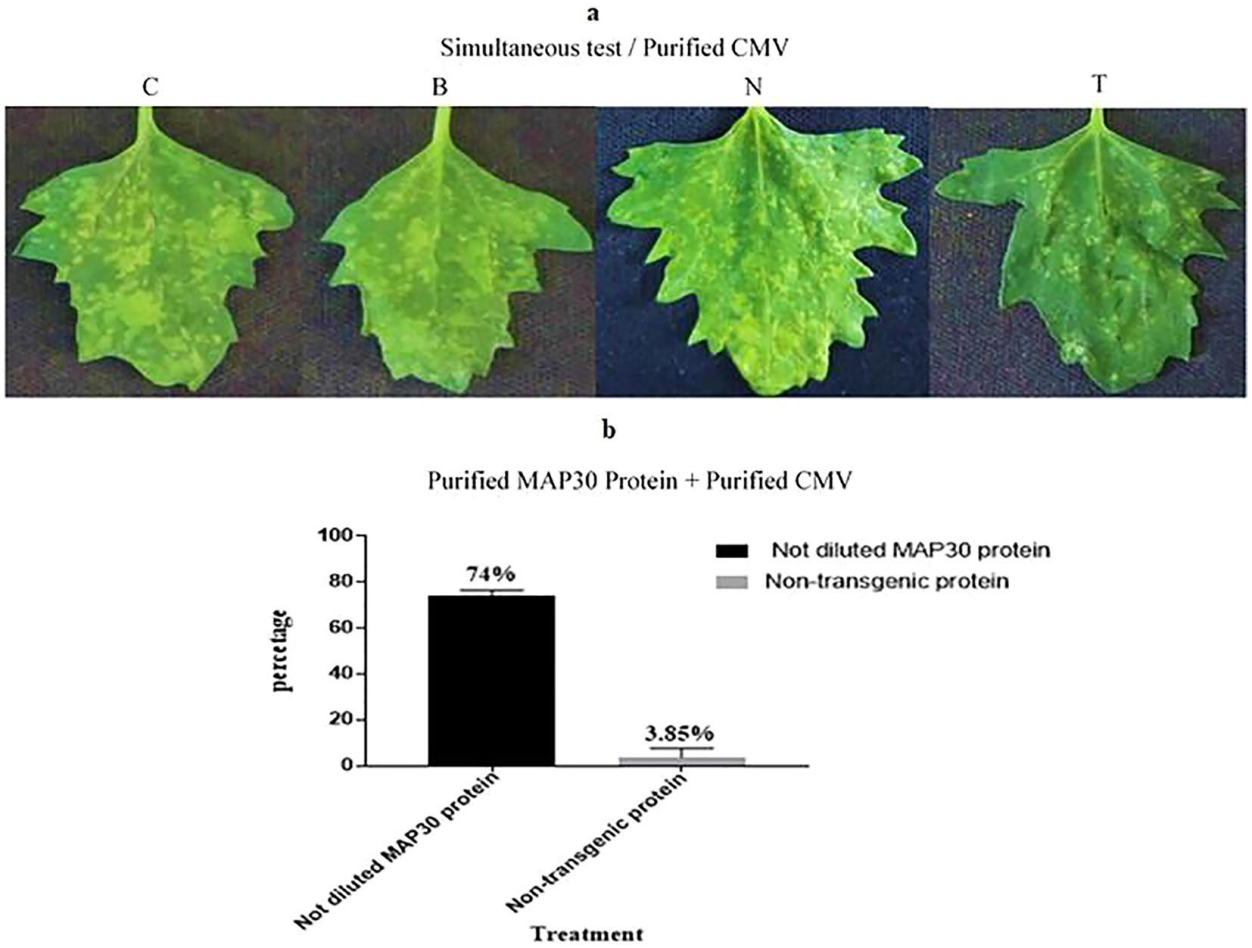
The inoculation of *C.* *quinoa* seedlings simultaneously with purified CMV and rMAP30 or non-transgenic protein. The extraction buffer (phosphate buffer) was considered as one of the negative controls (Table 4). There were 16 replicates for each test. **(a)** The assays performed by undiluted rMAP30 (T), extraction buffer (B), non-transgenic protein (N), and CMV (C). **(b)** Viral infection intensity graph for non-diluted transgenic rMAP30 versus non-transgenic protein and extraction buffer. As it could be observed in the figures and the chart, rMAP30 can control the infection up to 74% in the undiluted stats. Although the extraction buffer, non-transgenic protein, as the control samples, were implemented in order to achieve a better understanding, that they did not influence the viral infection reduction. In addition, it was found that the intensity of viral spots on the controls inoculated leaves was highly similar to the virus-inoculated leaves.

**Figure 11:**
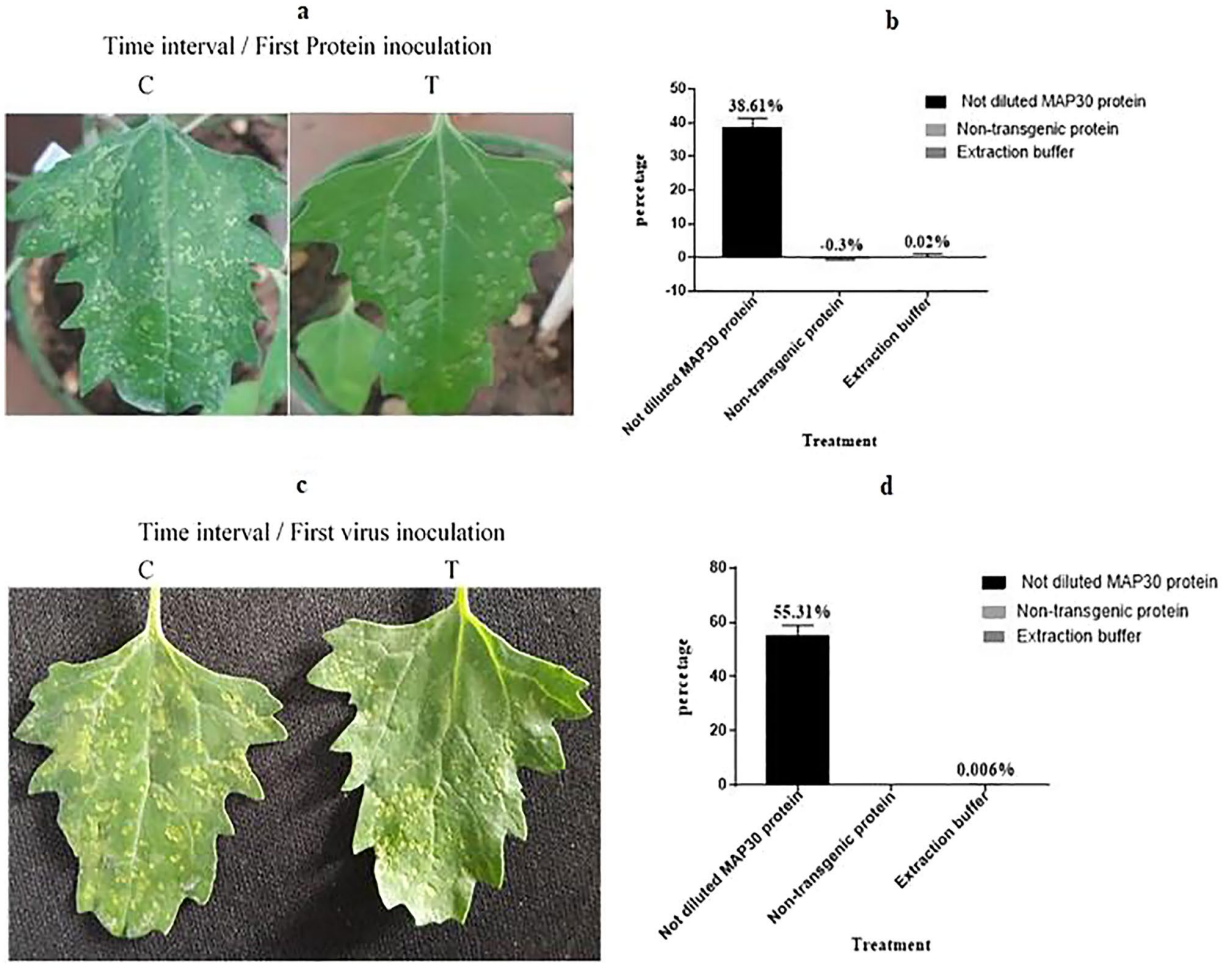
Inoculation of *C.* *quinoa* seedlings in the two-time interval assays with purified CMV and rMAP30. There were 8 replicates for each test. **(a,b)** The primary incubation with rMAP30 for 6 h, then apply viral infection on the leaves surface **(c,d)**. The primary incubation with viral infection for a 6 h, then apply rMAP30 on the leaves surface. It has been shown that the higher infection control observed when viral incubation has done 6 h prior than rMAP30. However, the control percentage in the initial treatment assay with rMAP30 is also significant and the number of viral spots is reduced.

Due to the fact that the rMAP30 showed the highest inhibitory effect on TMV, the experimental experiments against CMV were designed as following: (1) the effects of serial dilutions of rMAP30 on extraction-infected CMV in the simultaneous assays (Fig. 9), (2) the effects of undiluted rMAP30 against purified CMV in the simultaneous (Fig. 10), (3) the effects of rMAP30 against purified CMV in the time interval assays (Fig. 11) (Table 4). The highest inhibitory effect was up to 74% whenever the undiluted rMAP30 was used against the purified CMV simultaneously. Incredibly the control percentage reduced to almost 55% when rMAP30 was applied after 6 h of CMV infection; and on the other hand, 38.61% control percentage was observed whenever rMAP30 was applied 6 h before CMV infection. In general, one of the reasons for reduction in control percentage in rMAP30 implementation 6 h earlier than the virus, can be due to the large size of the rMAP30, so it cannot fully absorb through the leaf pores and as a result, it cannot play a full repressive. In the assays with extraction-infected CMV, the lowest control percentage (29.17%) was belonged to 1/ 4 dilution of rMAP30. In total, the application of rMAP30 in the all experimental cases against CMV with various dilutions (1/2 and 1/4) showed a significant positive effect on the infection.

Although there were not any investigations carried out on the possible effects of rMAP30 on plant viruses, the mechanism of action might be determined based on the investigations that studied various characteristics of MAP30 including antiviral properties against viruses such as HIV, HSV-1^16,26^ polio virus, coxsackie virus B3, and Epstein-Barr virus^15^. MAP30 also has an N-glycosidase activity that acts specifically on the glycosidic linkage between the ribose and A4324 or G4323 of the 28 s rRNA in a cell-free system^22^, and inhibits ribosomal protein synthesis in the infected cells^43^. Generally, the mechanisms of action of the last item could be resulted from cutting in the viral genome, *N*-glycosidase activity, creating a fracture in the protein synthesis, and changes in the viral proteins; furthermore, may involve a similar positive effects on the animal, human, and plant viruses through the same mechanisms.

## Conclusions

It was observed in current study that the recombinant MAP30 shows appropriate anti-bacterial and anti-fungal properties, in the unpurified purified or even concentrated forms. The In vivo anti-viral properties against two important plant viruses, CMV and TMV, showed a significant reduction in the number of infection spots. After the protein dilution, the viral infection was intensified. The highest viral infection reduction was observed using undiluted rMAP30. In the time interval assays (MAP30 inoculation, 6 h before and after the viral infection), a positive effect on the control of both viruses was observed. The more infection reduction after the viral infection emphasized on the assumption of the challenge for direct and complete absorption of protein into the leaf surface due to its size; however, it did not have any conflicts with the positive and influential results. The possible inhibitory effect of recombinant MAP30 on the *E.* *coli*-specified bacteriophage, which was extracted and purified from the urban wastewater, could be due to the topological change, especially DNase-like activity of this protein on genomic DNA, and may leads to its inability in the next host infection stages; therefore, the phage agent will not have the 100% infection potential. Significantly, the phage controlling power was more obvious in the time interval assay. The number and diameter of plaques decreased as a result of the treatment time enhancement. These findings are very significant, especially in the dairy industry, which lacking of a fundamental and practical method against bacteriophage, for instance, destructive bacteriophages for lactic acid bacteria, can causes so much damages. And in this article, we can claim that the recombinant MAP30 protein was the first plant protein with anti-human virus properties that was able to show significant properties in the field of controlling plant viruses and phage can claim that its application in practical formulations as a biological safe agent against viruses, fungi, and bacteria might act as a prophylactic even before the infection, in order to reduce the crop diseases-caused damages.

## Supplementary Information

Supplementary Information.

## Data Availability

All data generated or analyzed during this study are included in this published article.
