# Supplementary material for: Recombinant anti-HIV MAP30, a ribosome inactivating protein: against plant virus and bacteriophage
Source: Sci Rep. 2023 Feb 6;13:2091. doi: 10.1038/s41598-023-29365-7 (PMC9902390; doi:10.1038/s41598-023-29365-7)
Supplement: Supplementary file 1 — Supplementary Information. [file 41598_2023_29365_MOESM1_ESM.pdf]

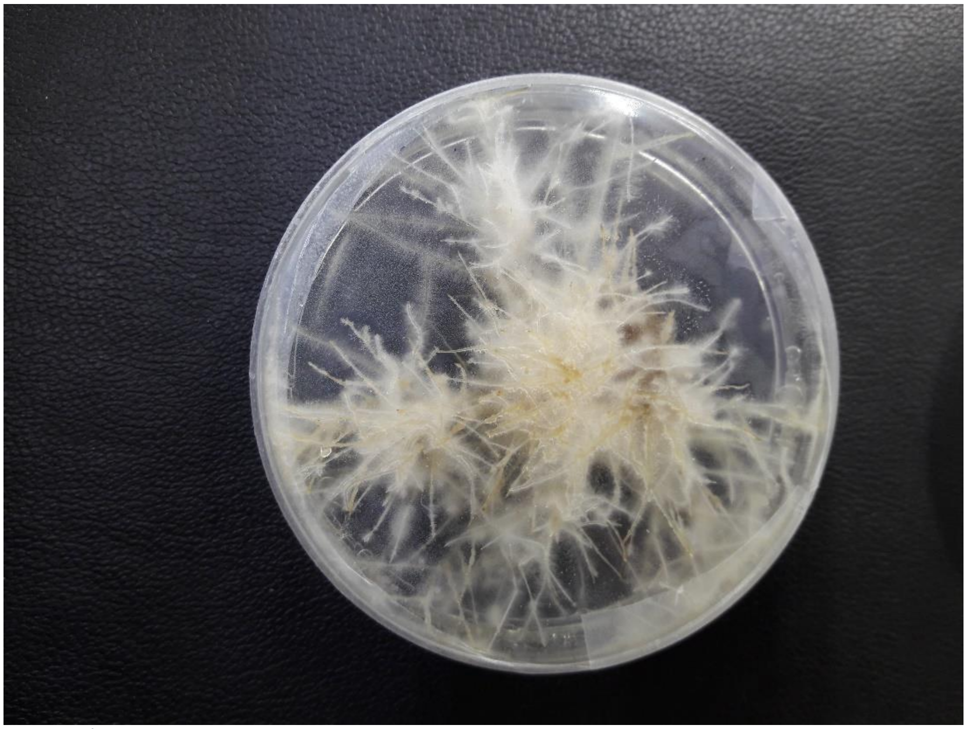

Figure 2/ image A left

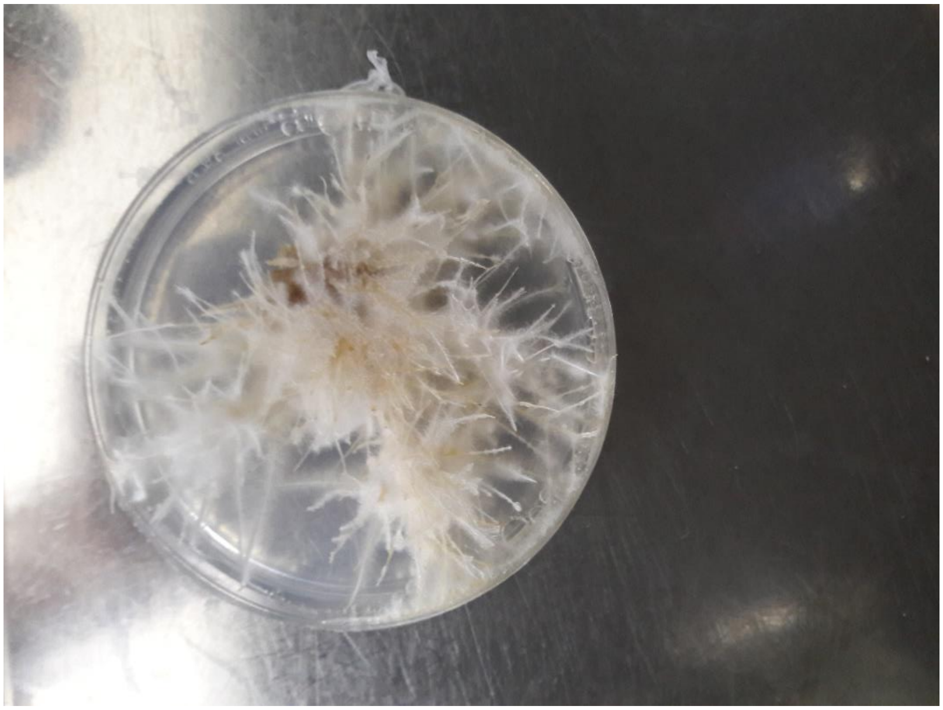

Figure 2/ image1A right

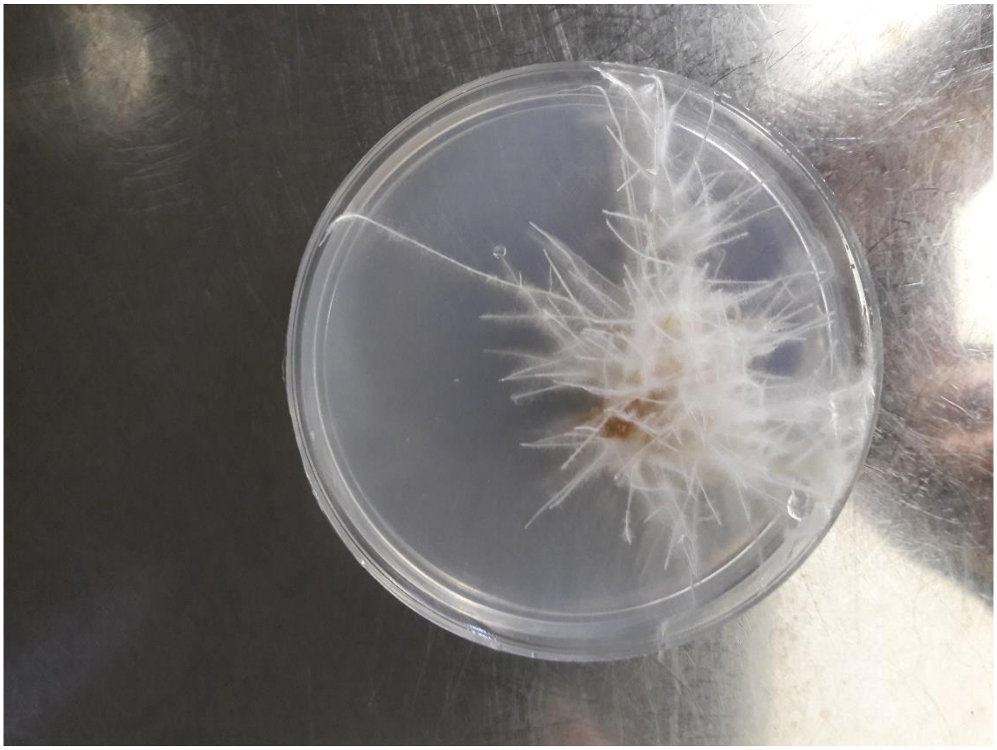

Figure 2/ image b transgenic

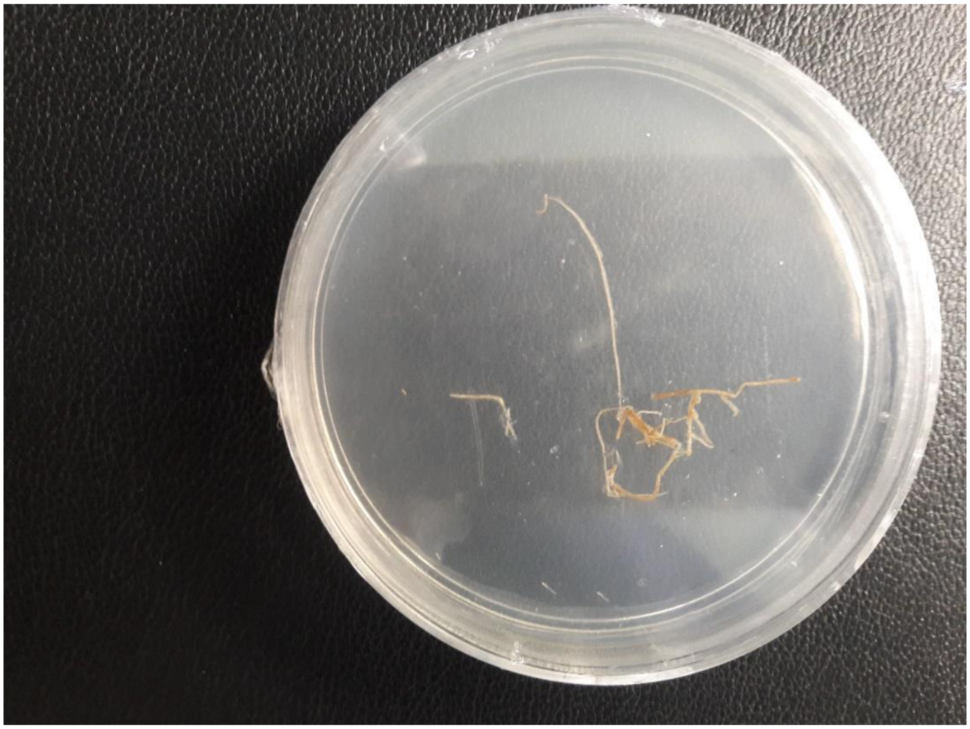

Figure 2/ image b non transgenic

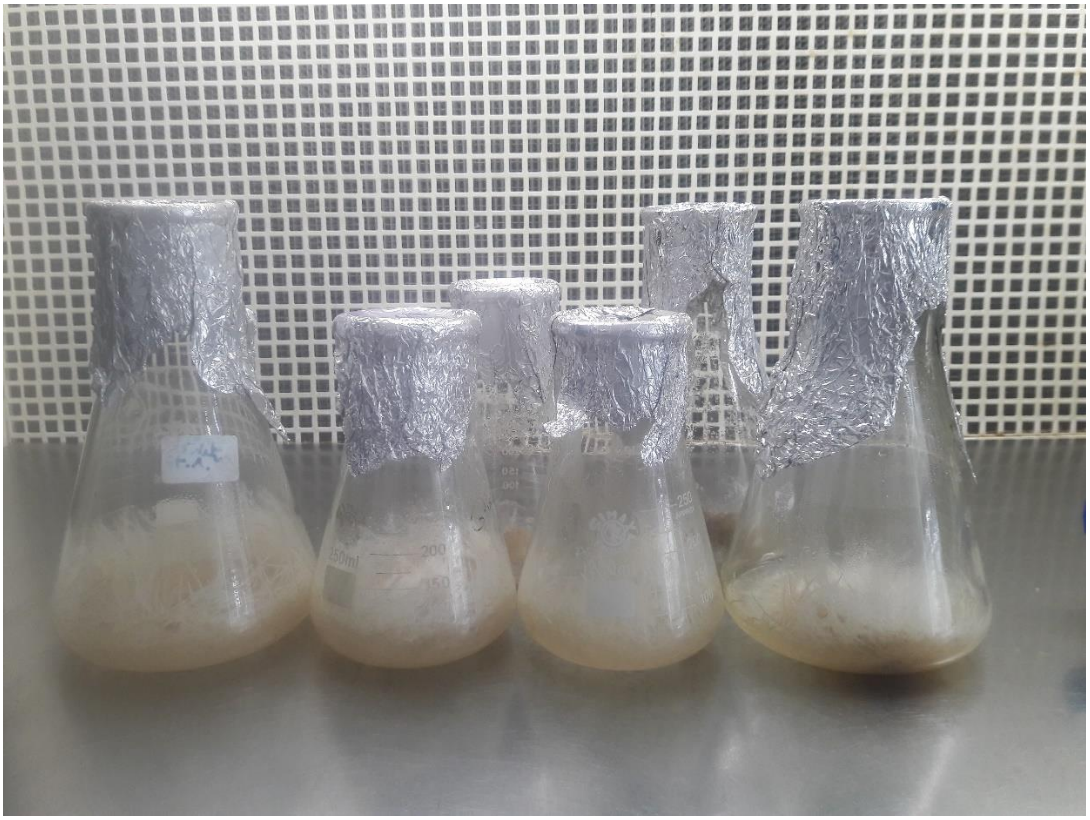

Figure 2/ c-down-left

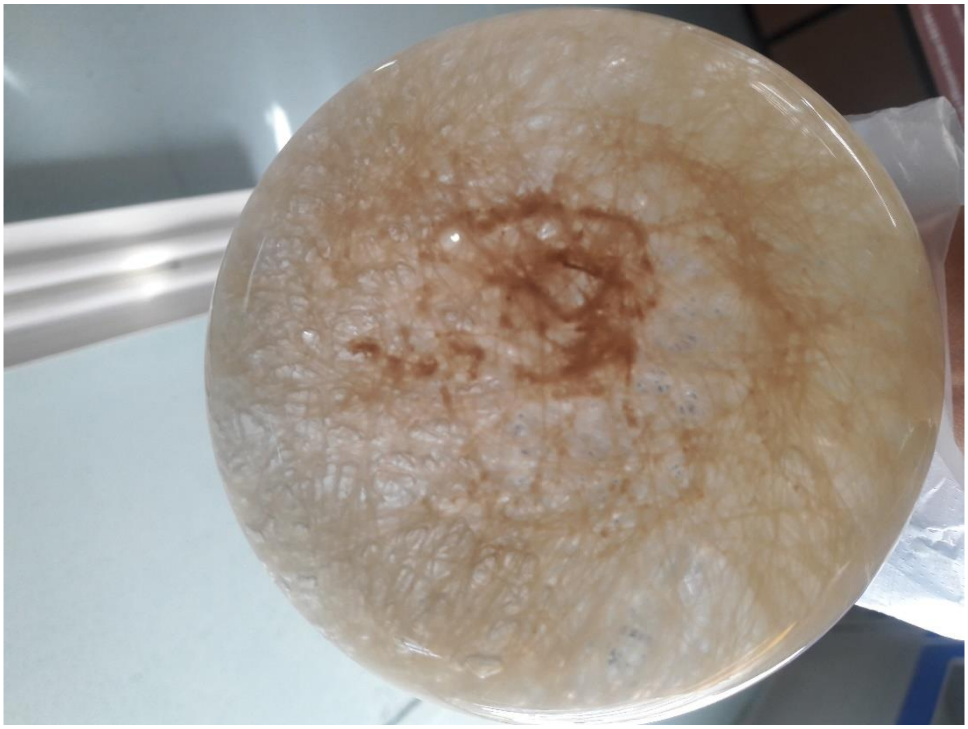

Figure 2/ c-middle

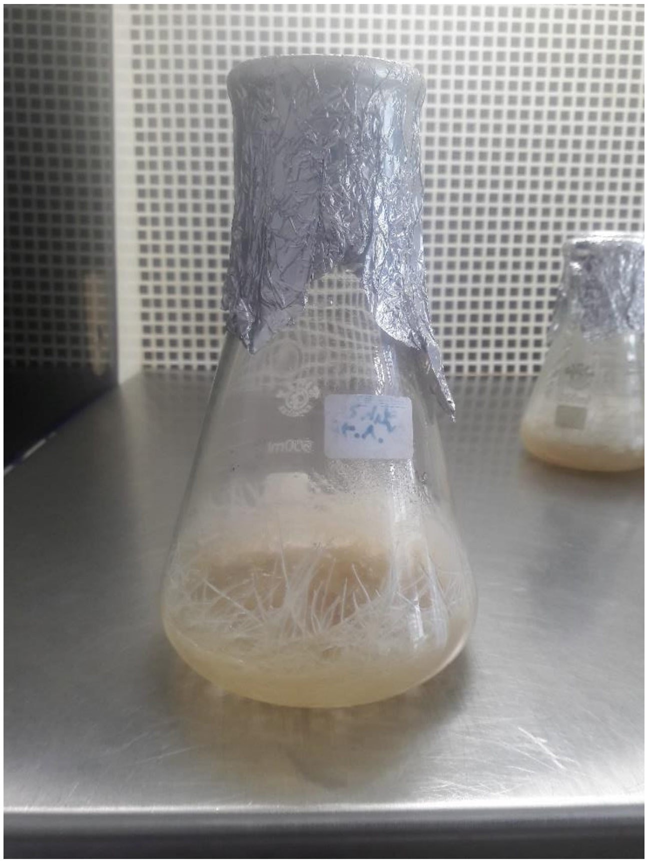

Figure 2/ c-right

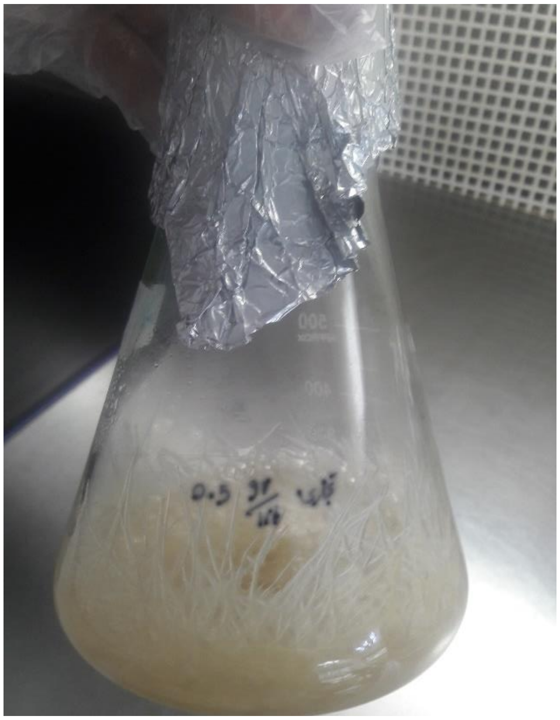

Figure 2/c-up-left

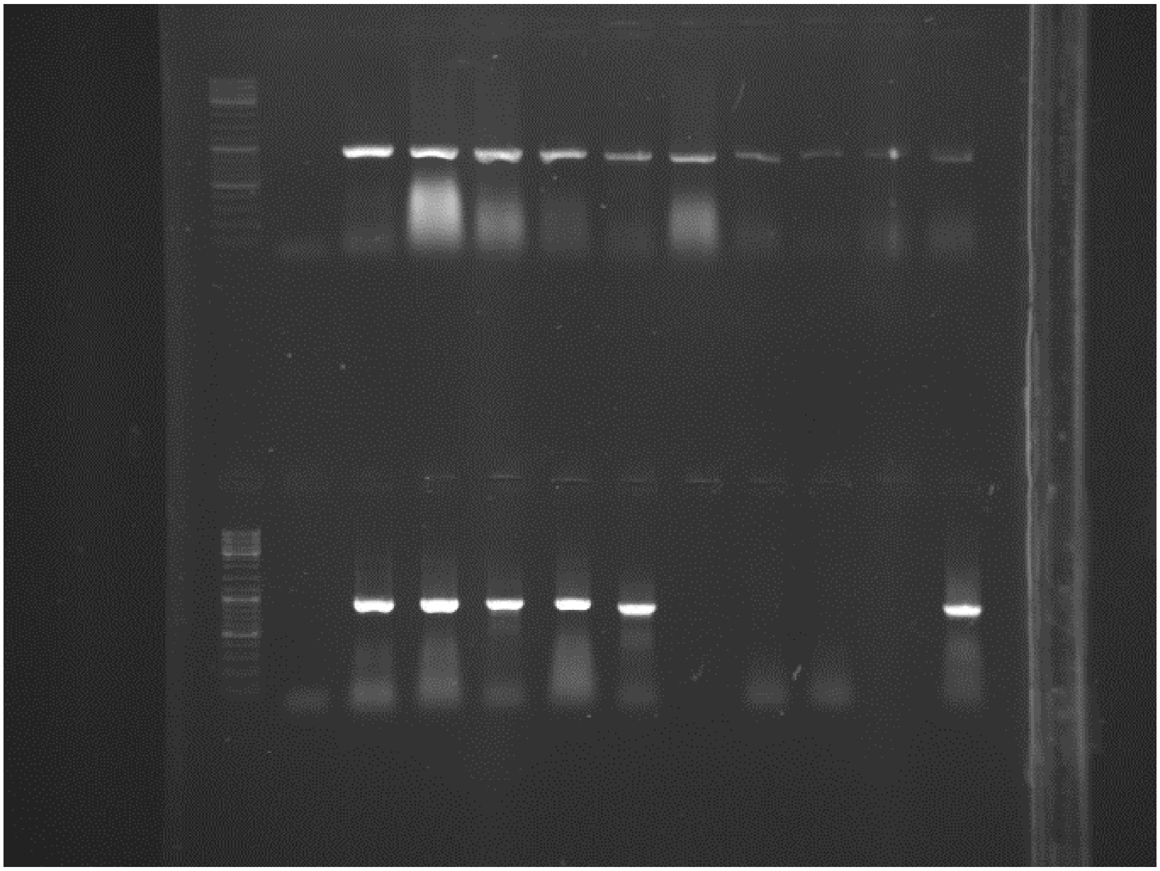

Figure 3/a: The bottom row is the gel we are looking for amplified fragment using MAP30 primer and DNA samples

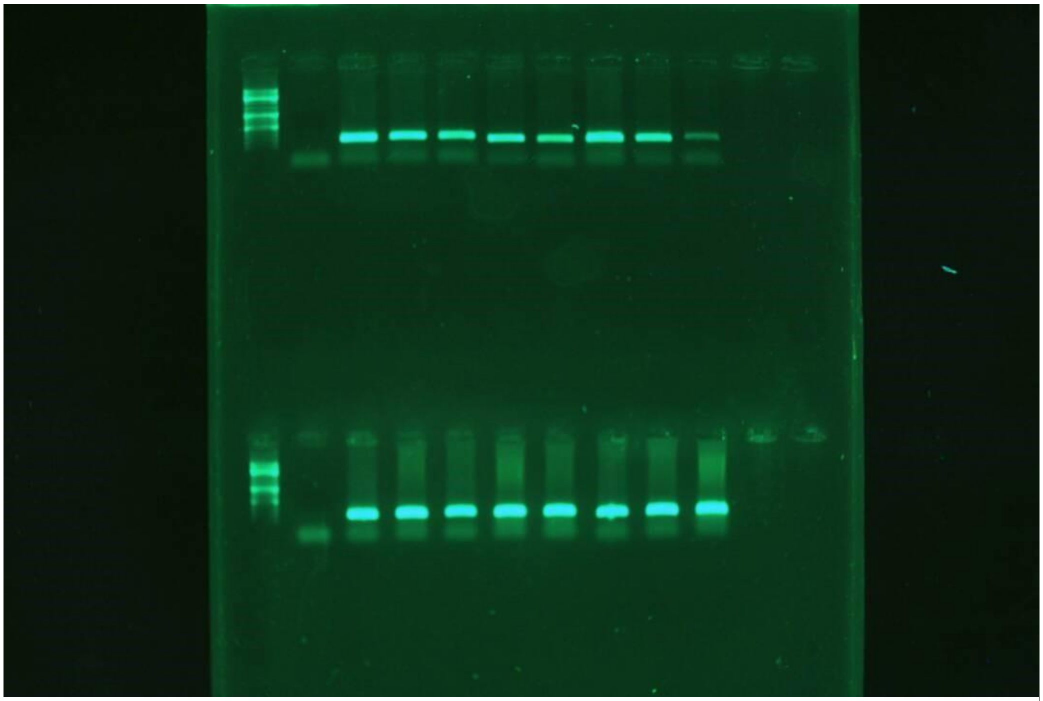

Figure 3/ b: The bottom row is the gel we are looking for amplifies fragment using rolB primer and DNA
Figu samples

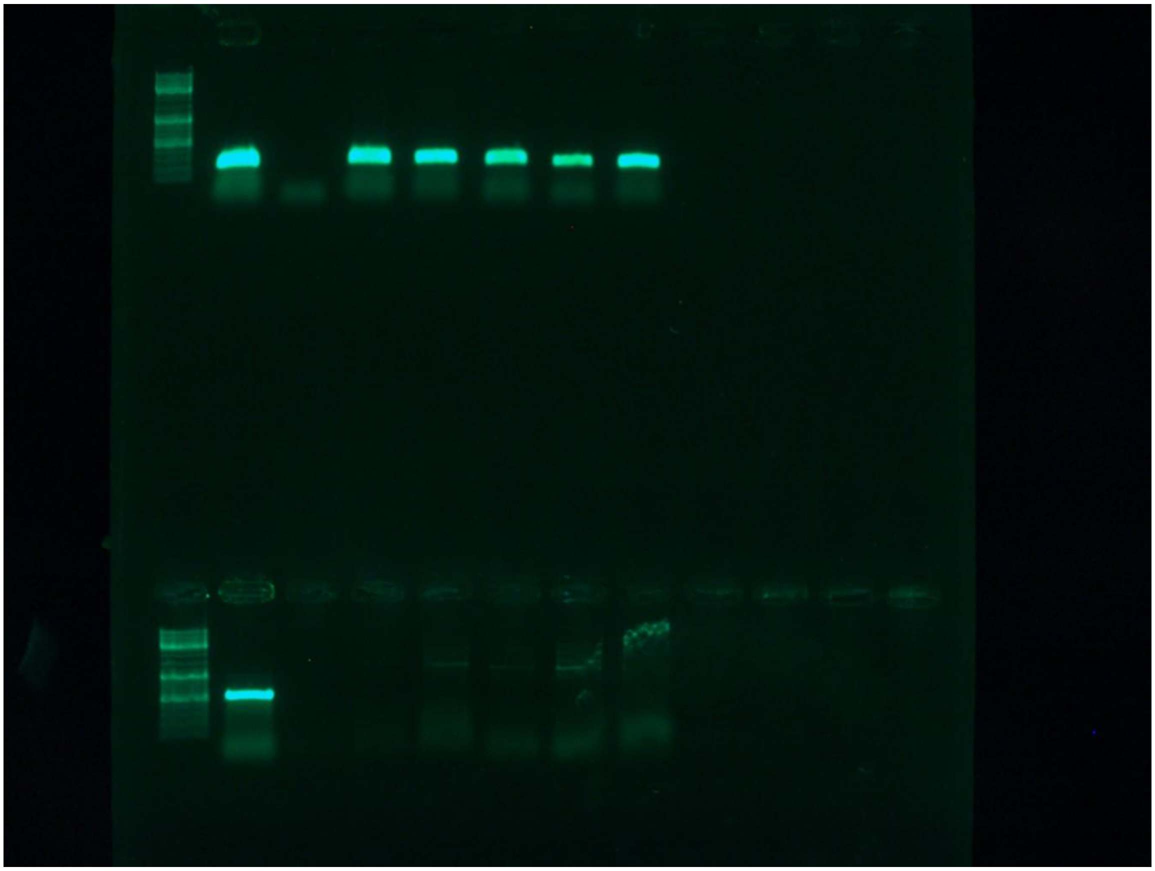

Figure 3/ c: The bottom row is the gel we are looking for amplifies fragment using virG primer and DNA samples

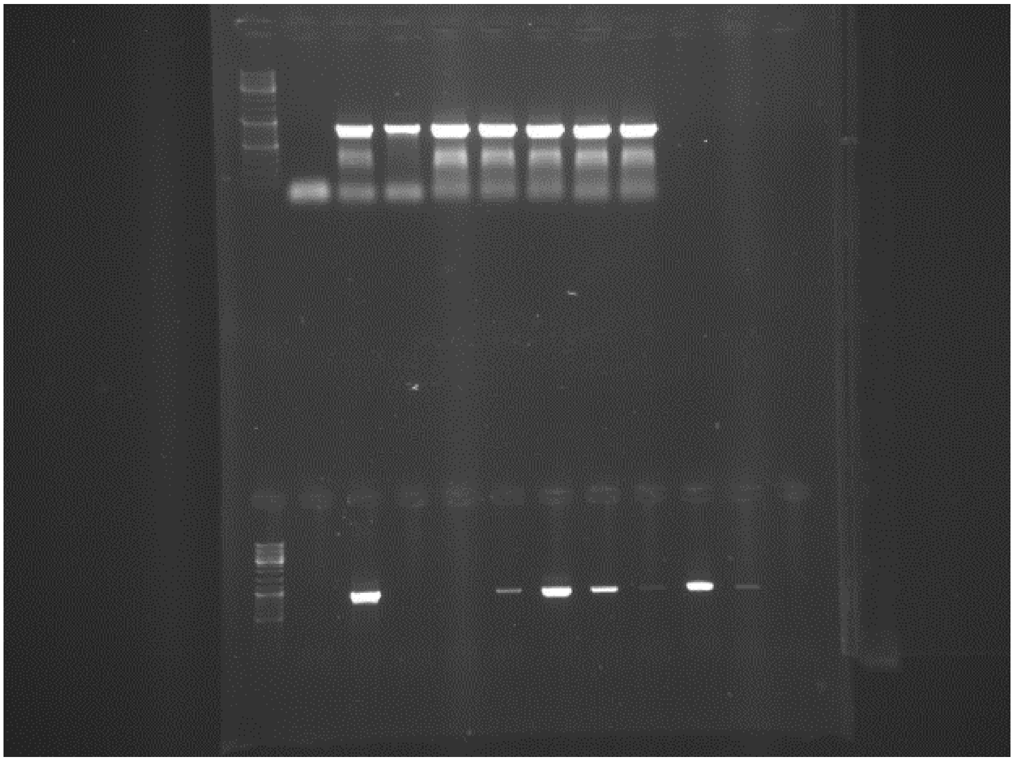

Figure 3/ d: The bottom row is the gel we are looking for amplified fragment using MAP30 primer and cDNA samples

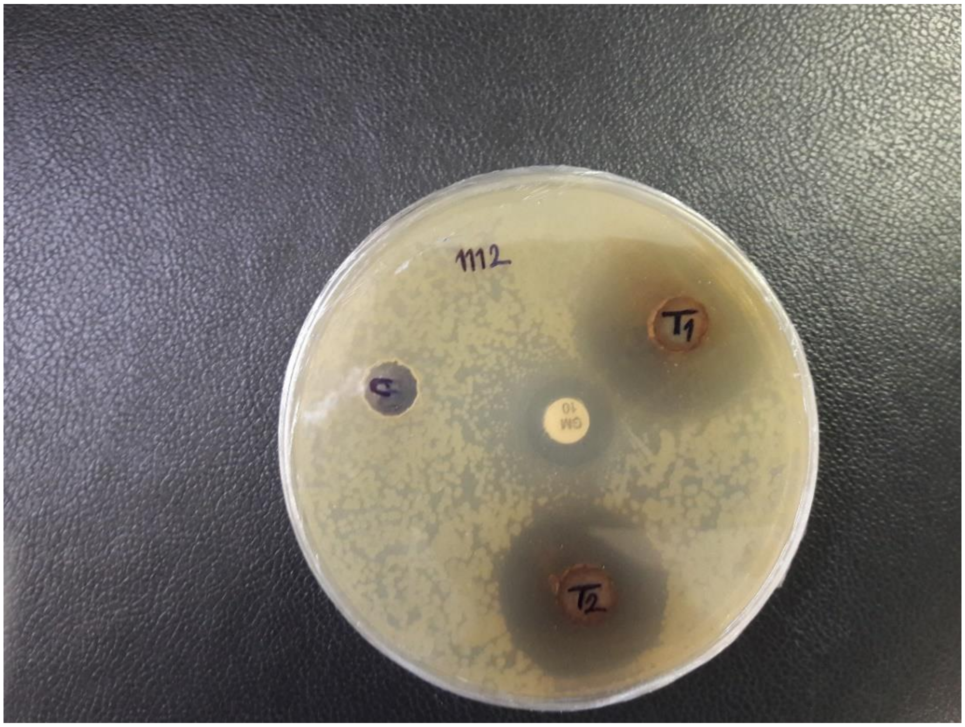

Figure 4/ A

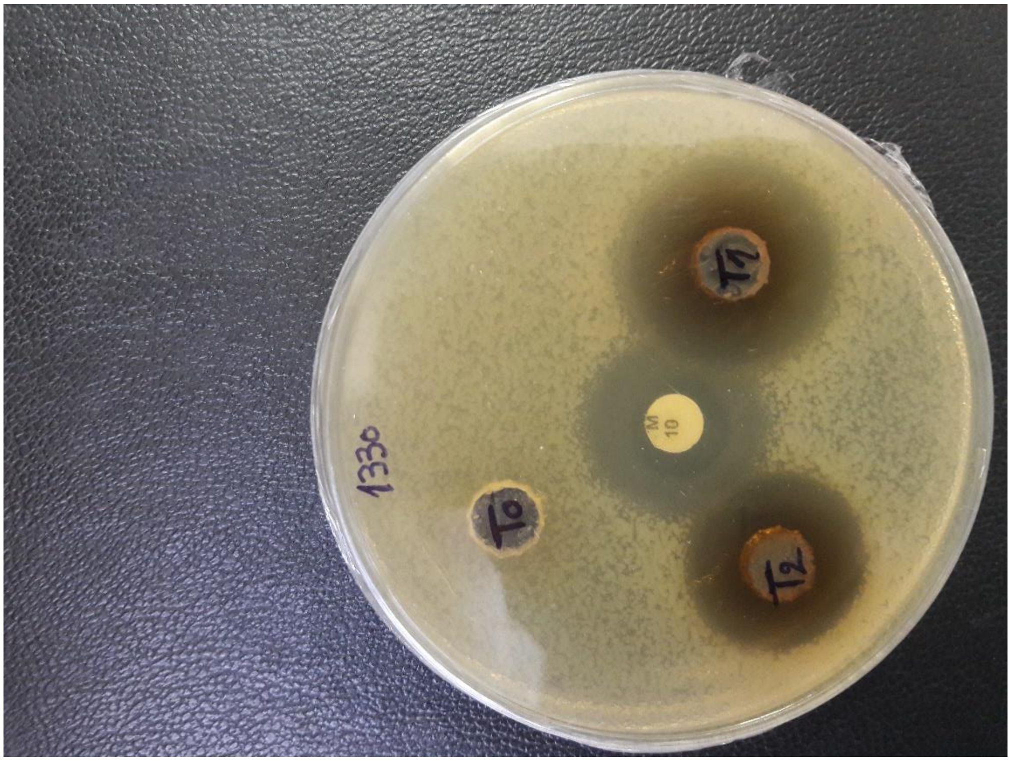

Figure 4/B

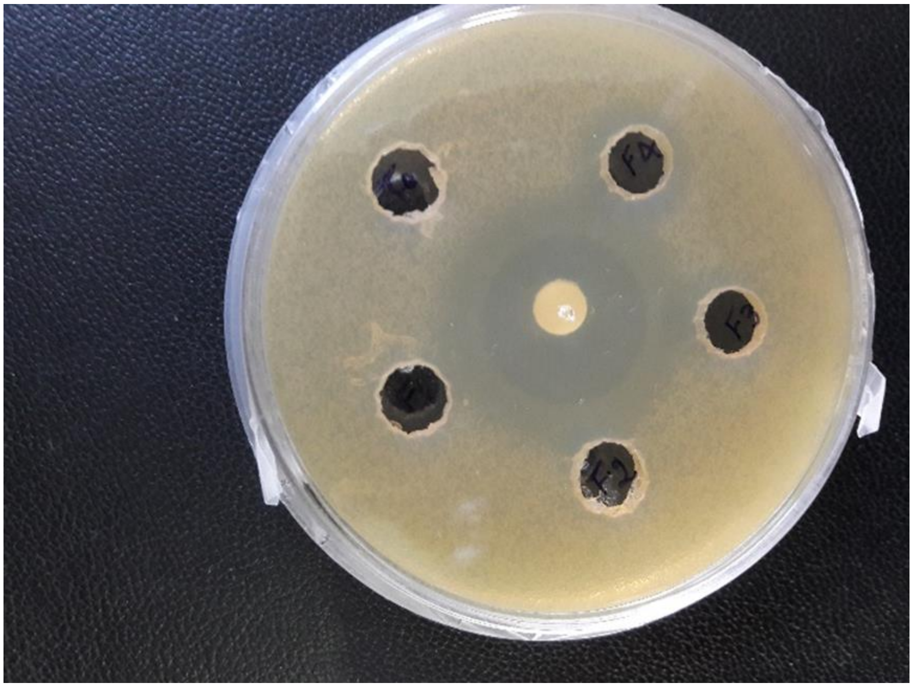

Figure 4/C

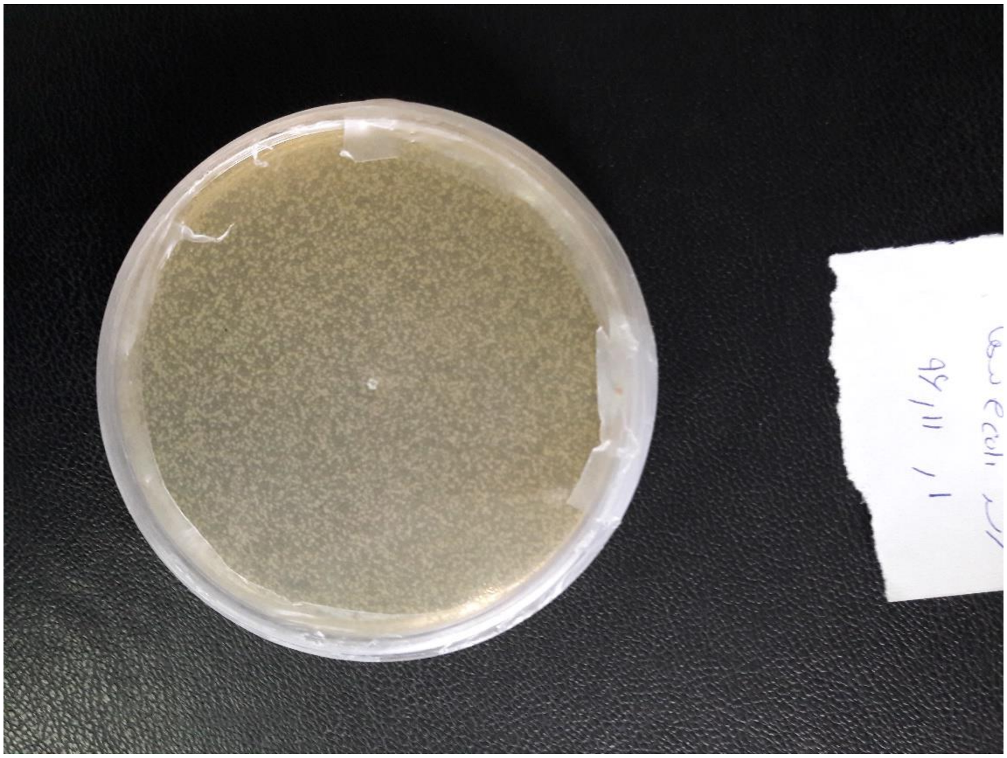

Figure 5/ a

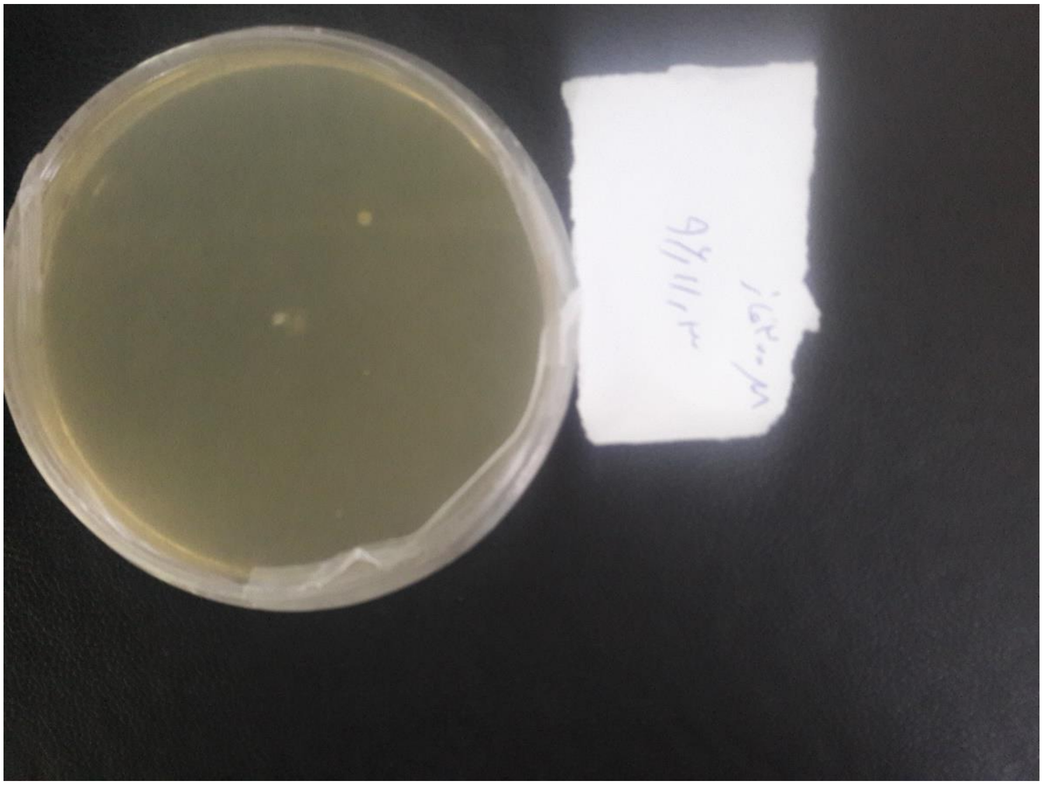

Figure 5/ b

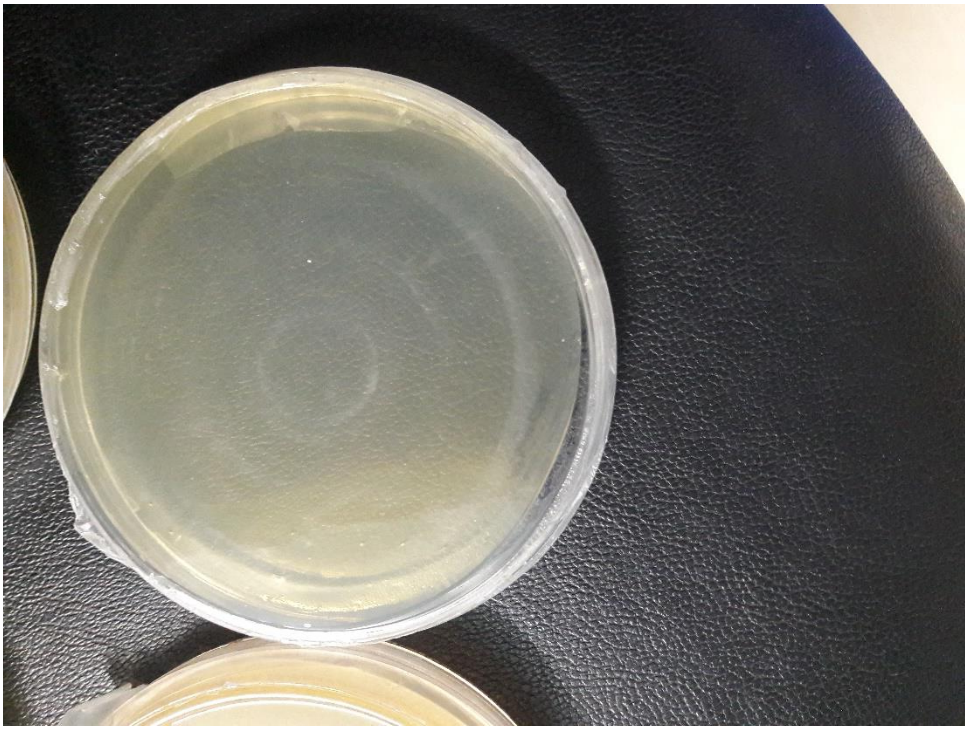

Figure 5 / c

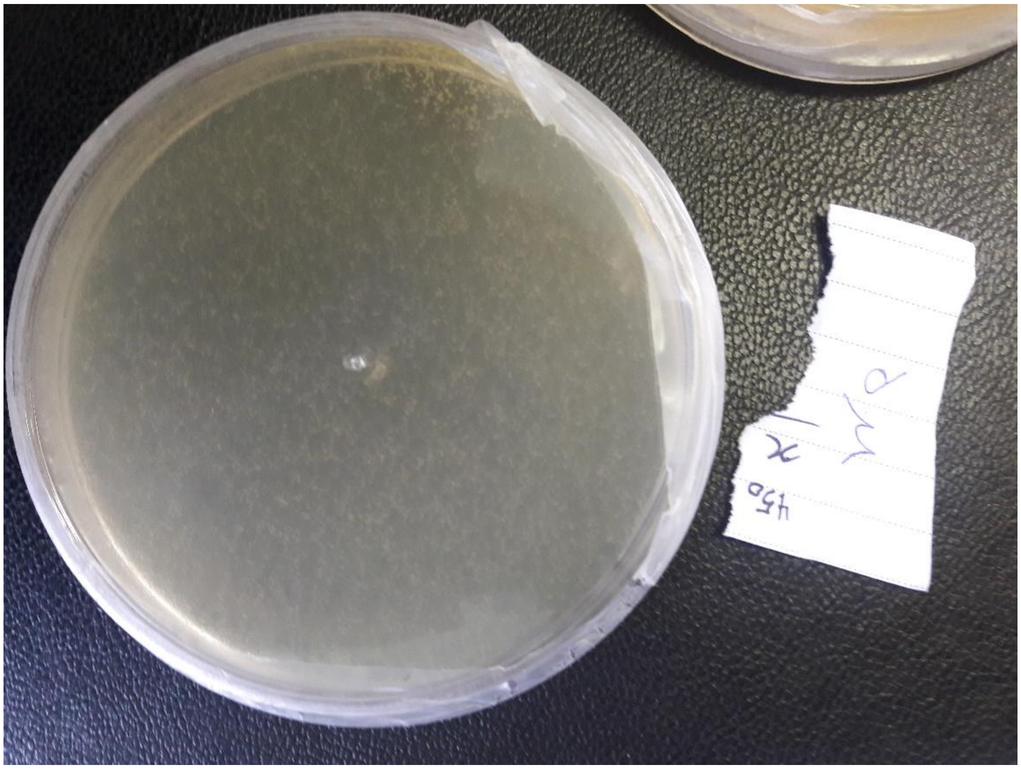

Figure 5 / d

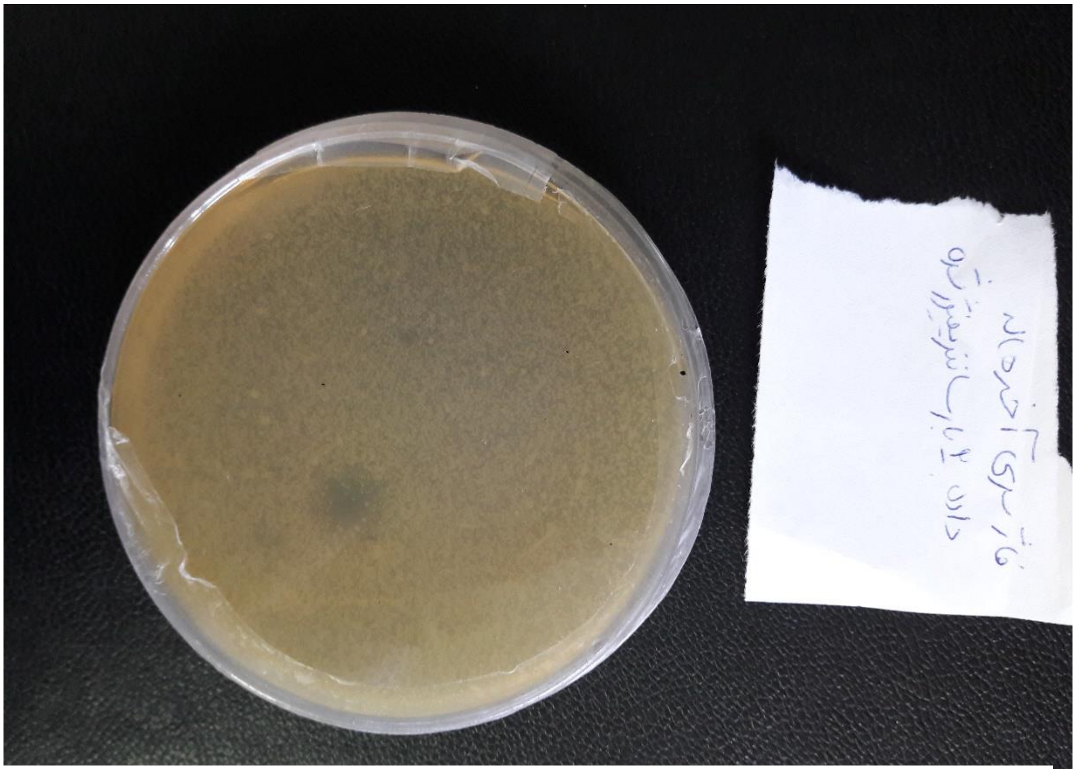

Figure 5 / e

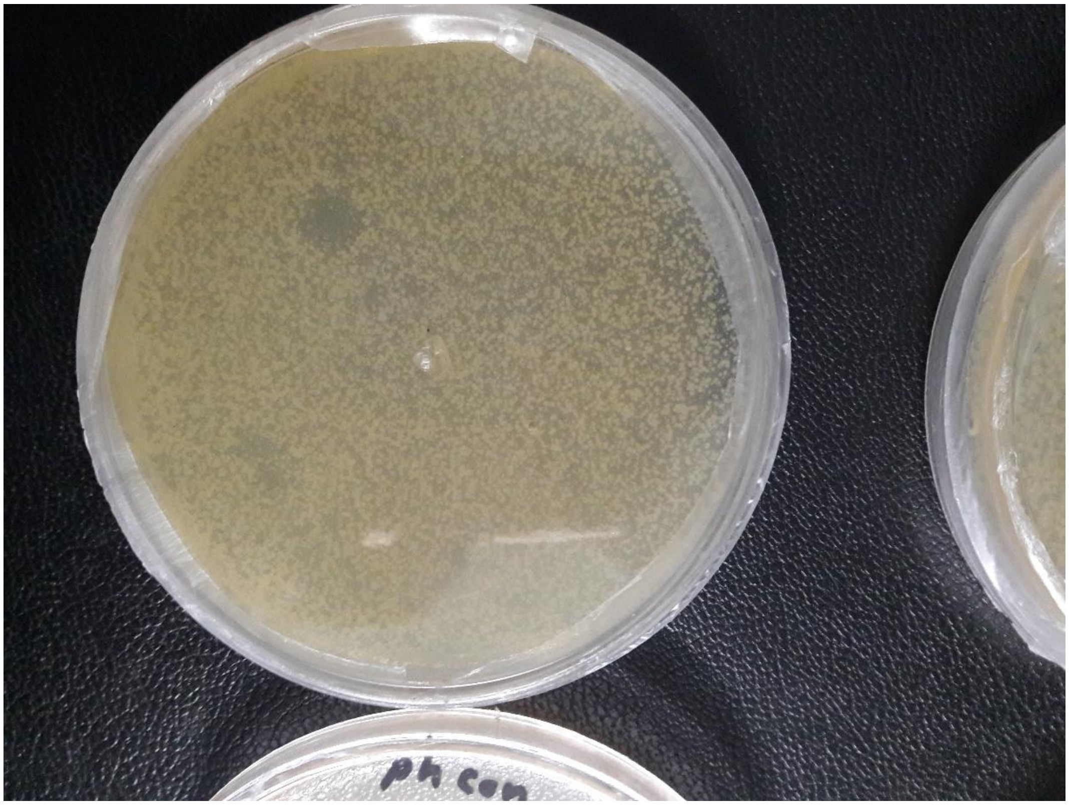

Figure 5 / f

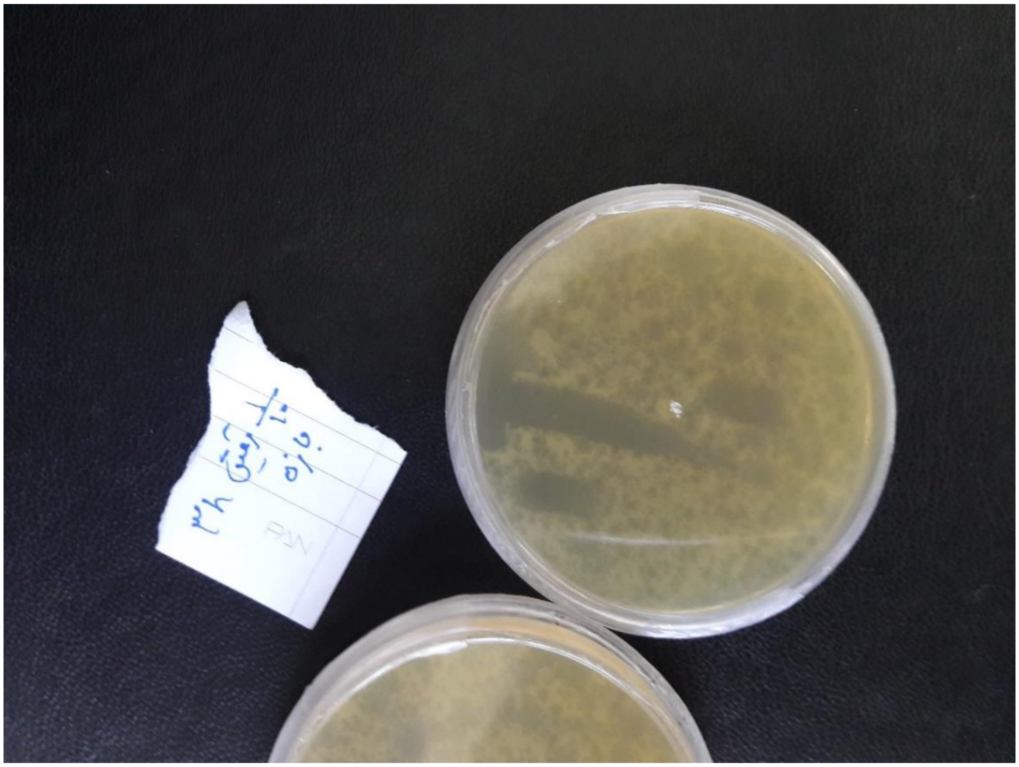

Figure 6 / a- right

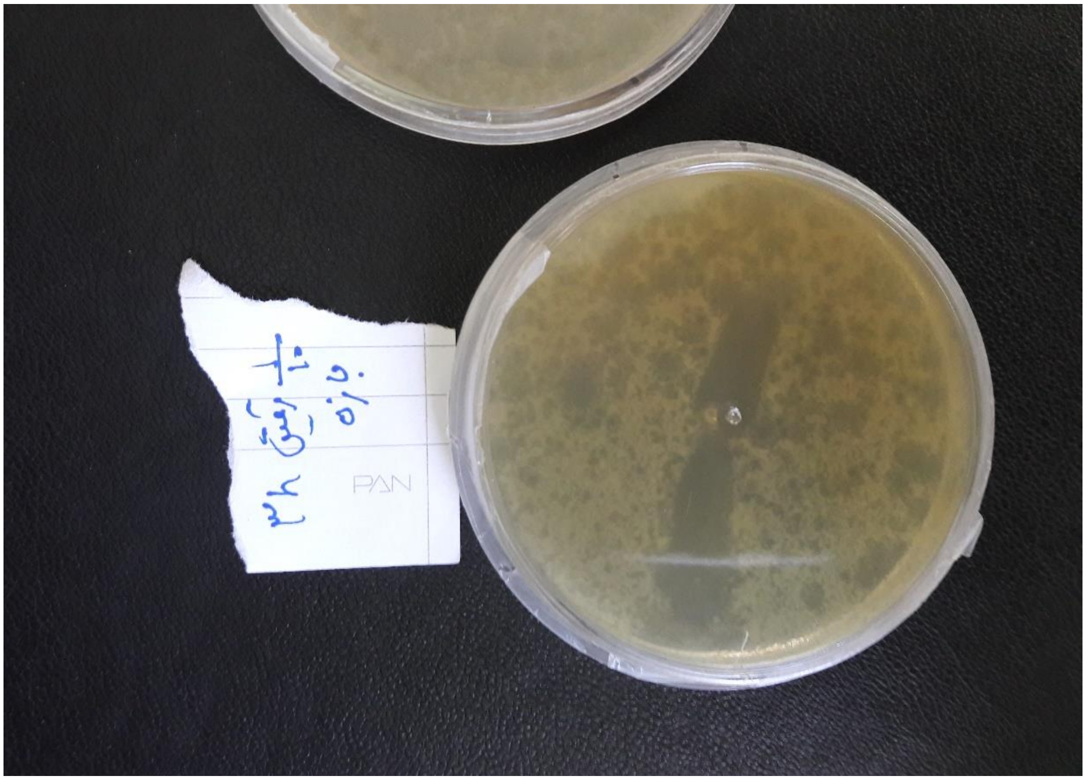

Figure 6 / a- left

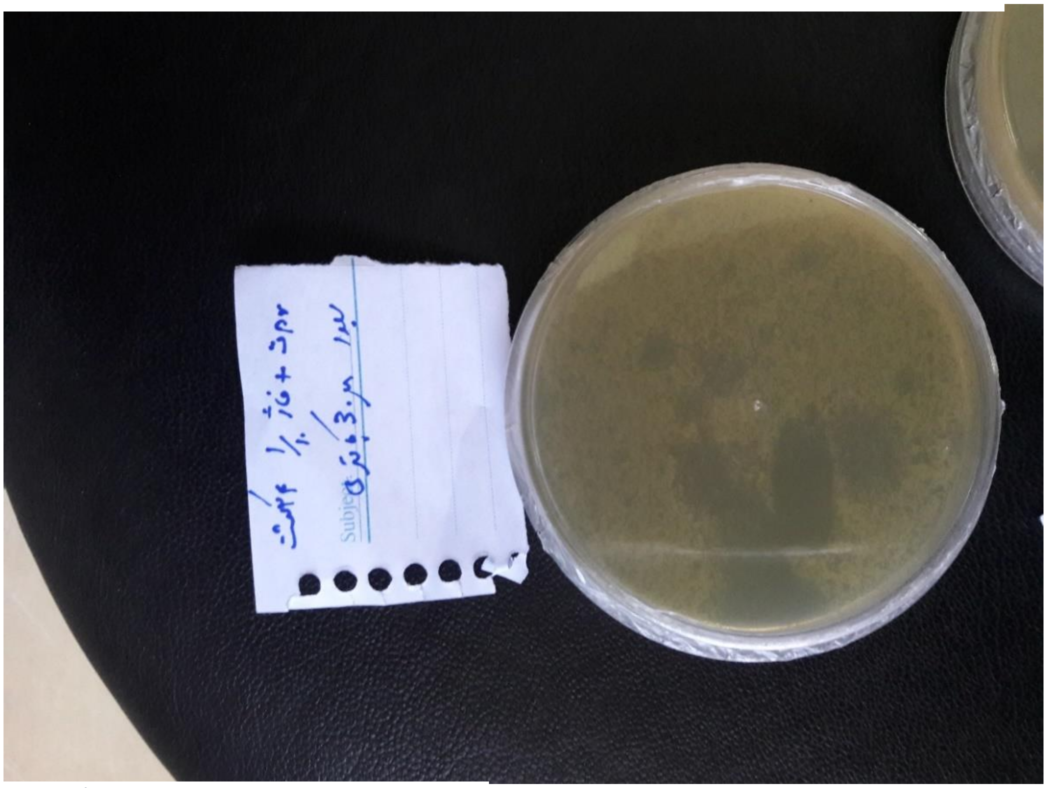

Figure 6/ b- left

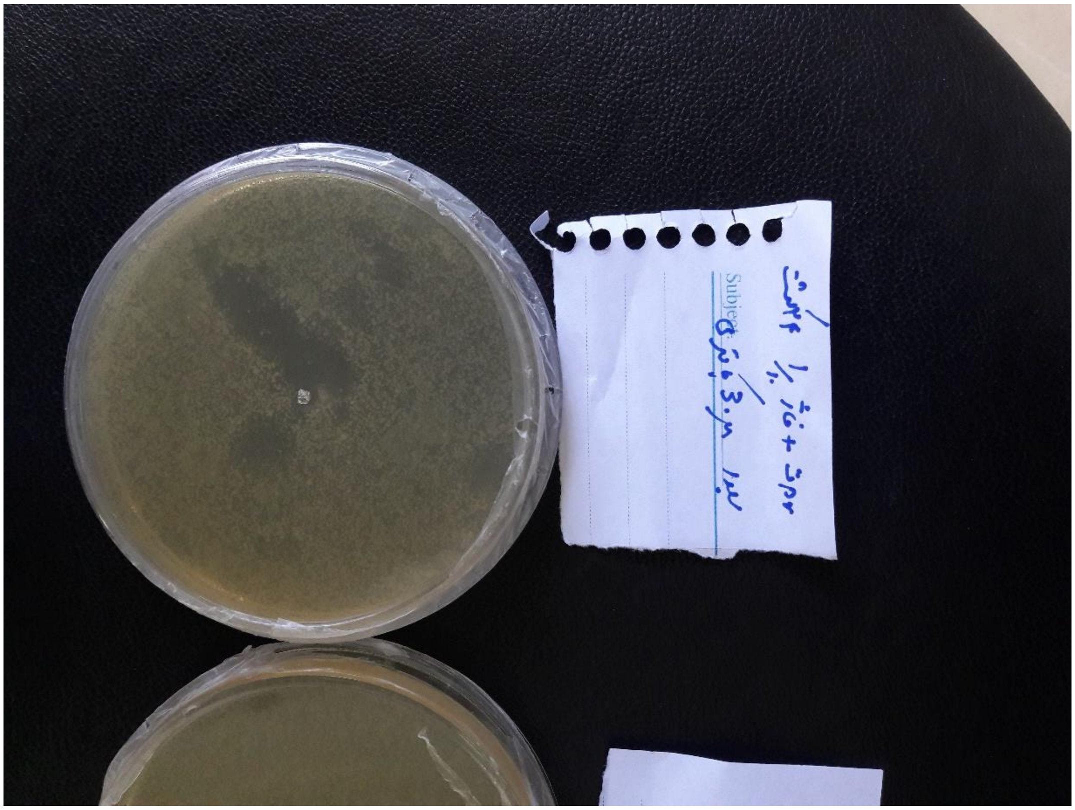

Figure 6/ b- right

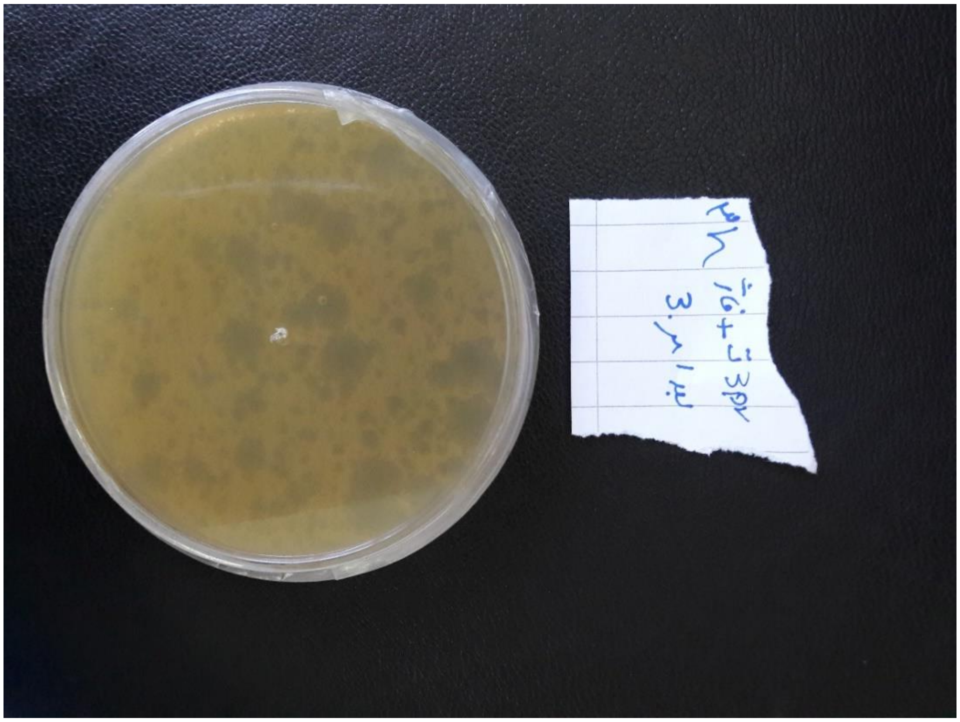

Figure 6 / C right

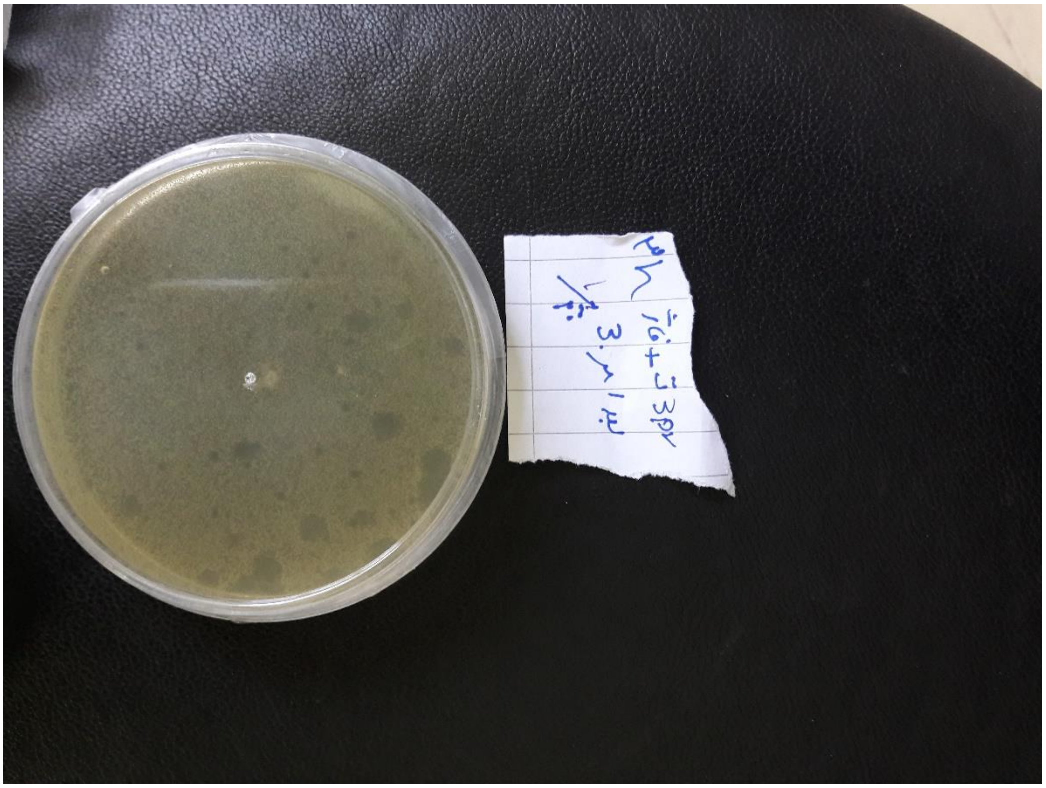

Figure 6 / c left

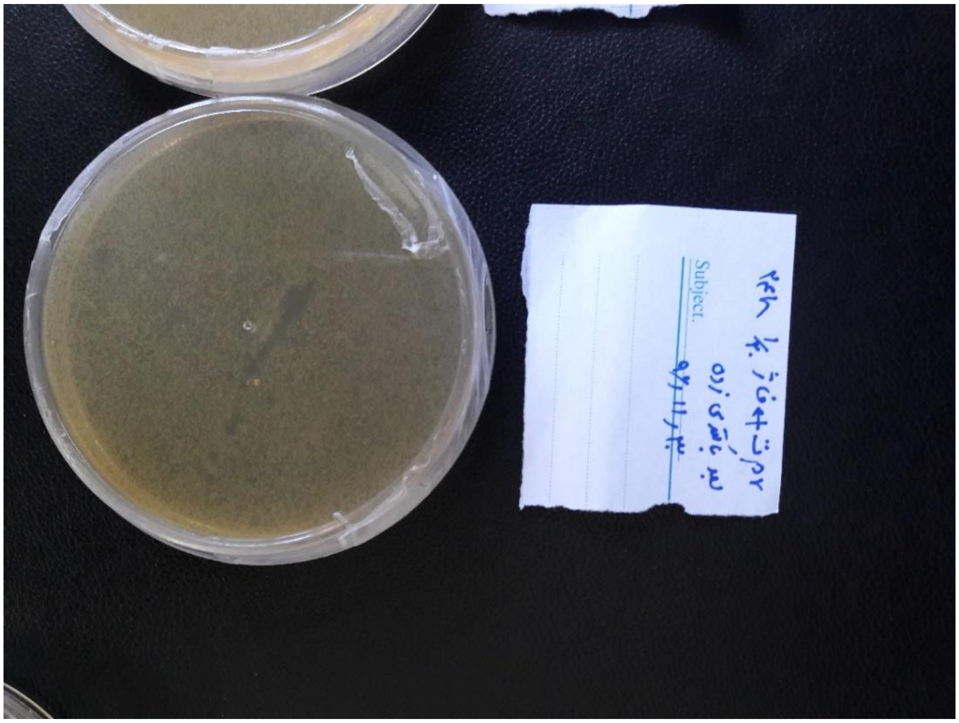

Figure 6 / d left

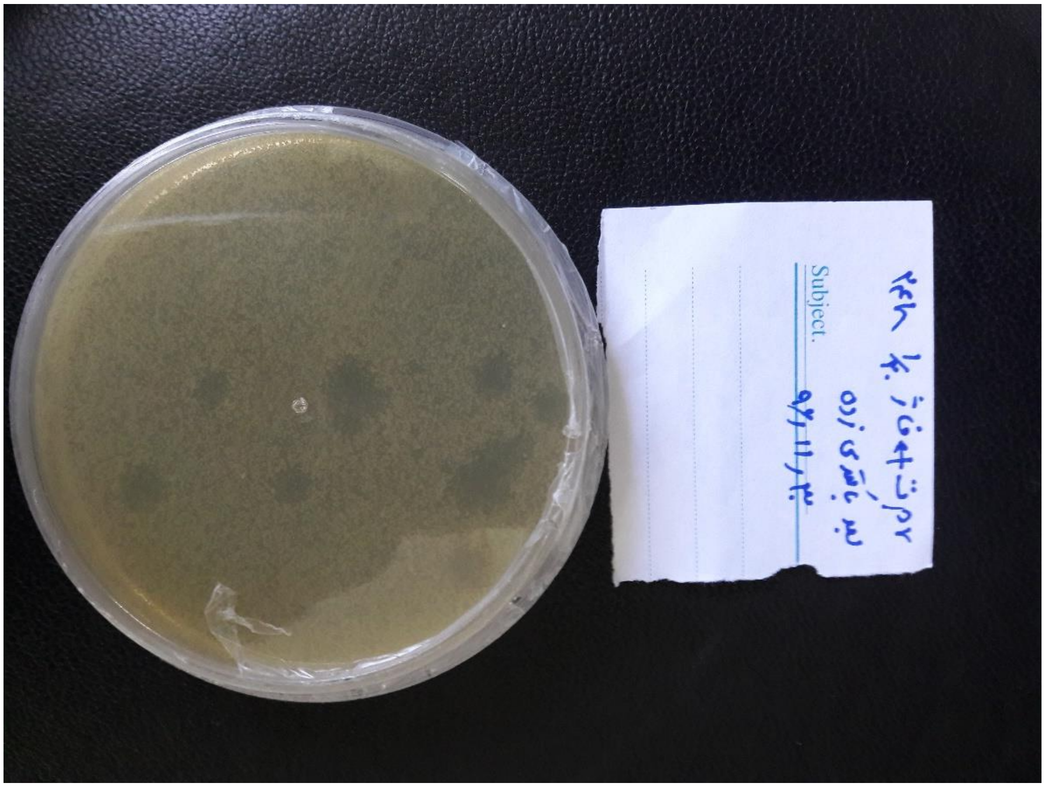

Figure 6 / d-right

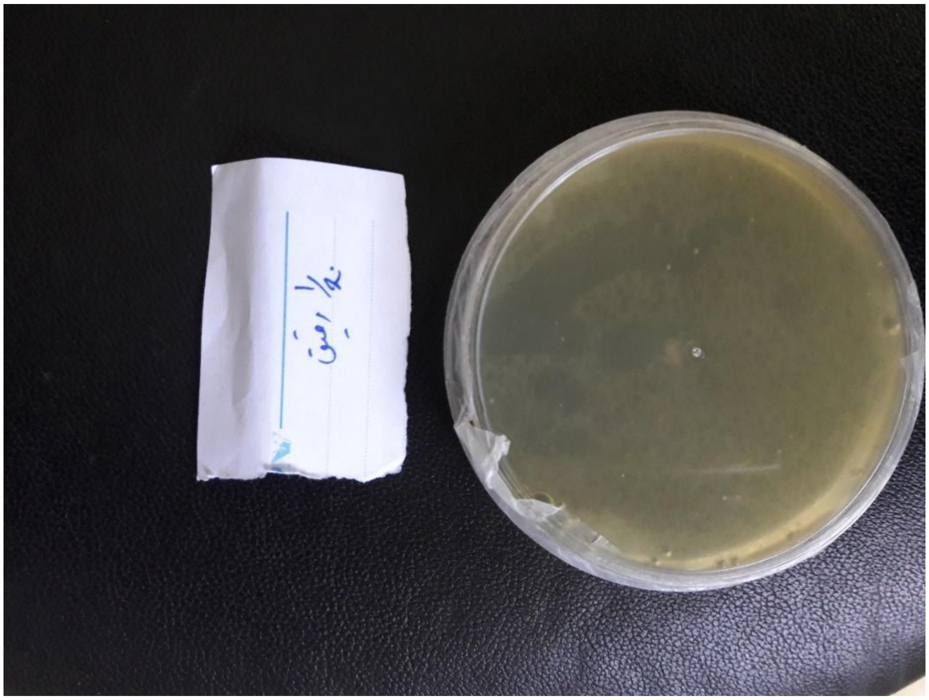

Figure 6 / e

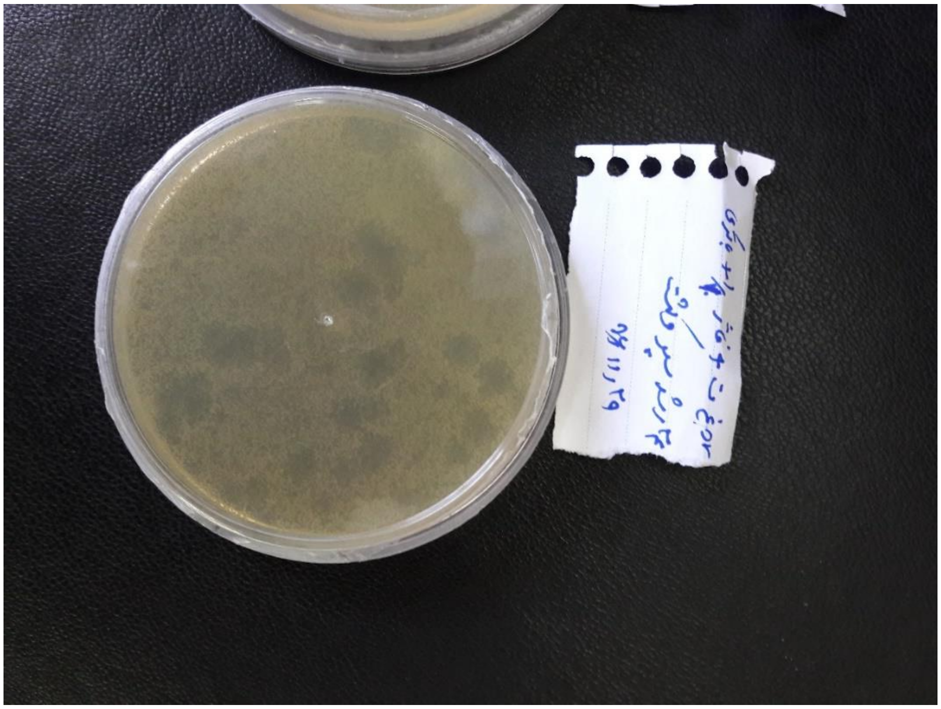

Figure 6/ F

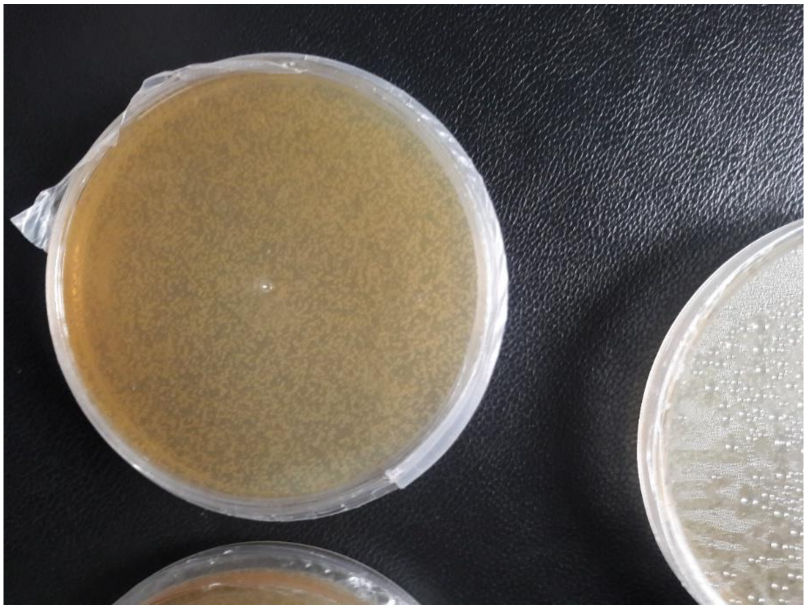

Figure 6/ g

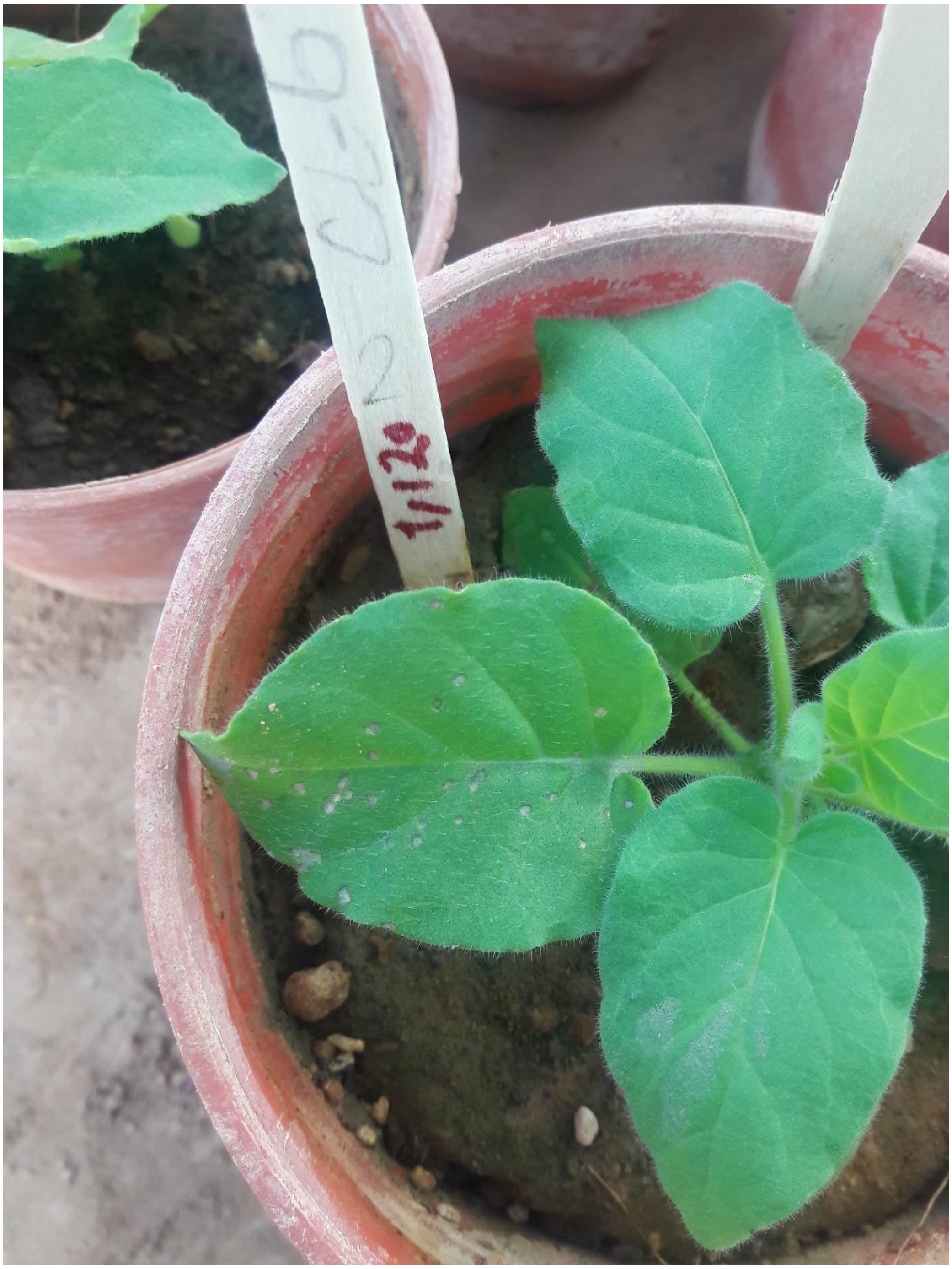

Figure 7 / a

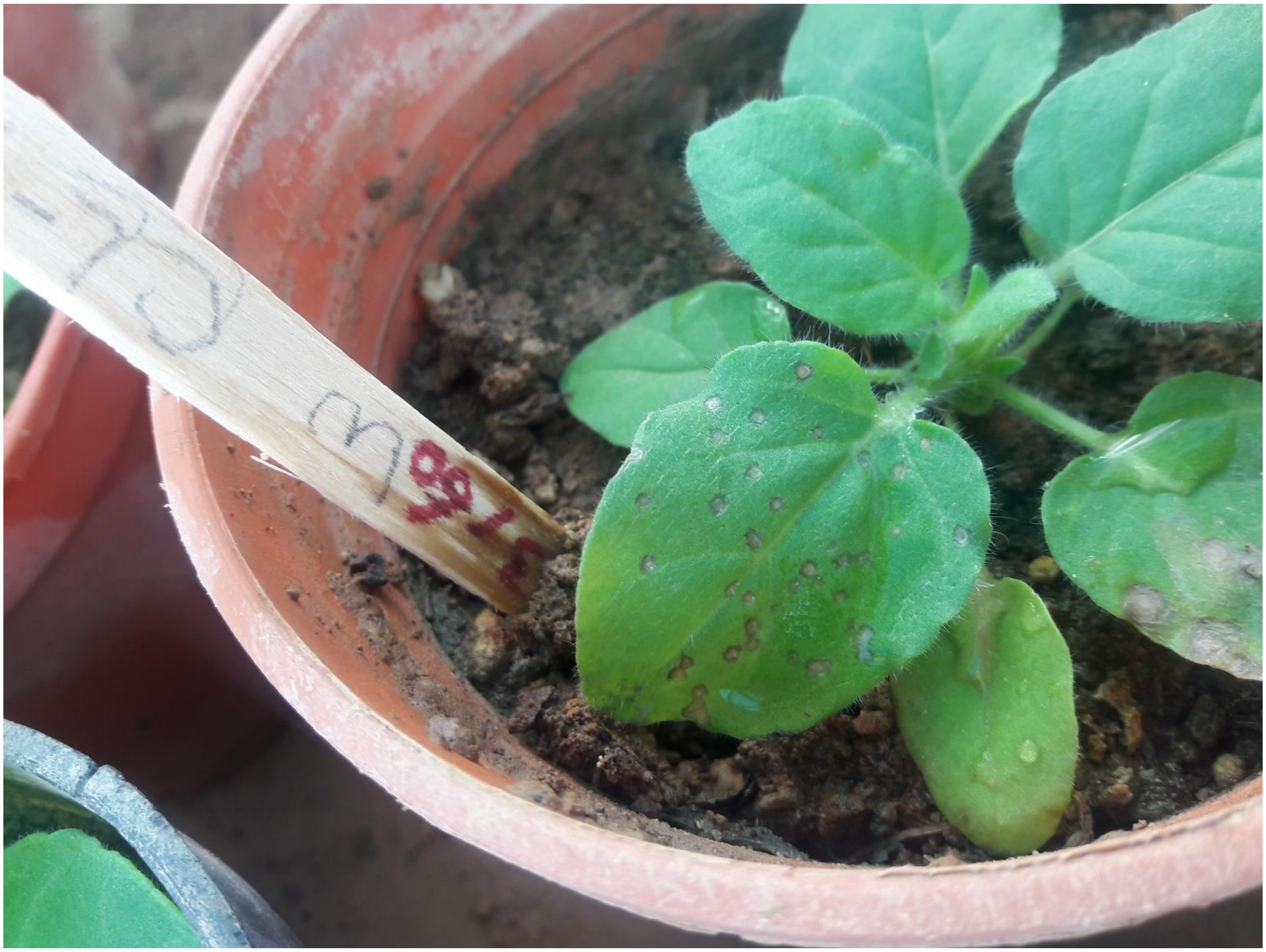

Figure 7 / b

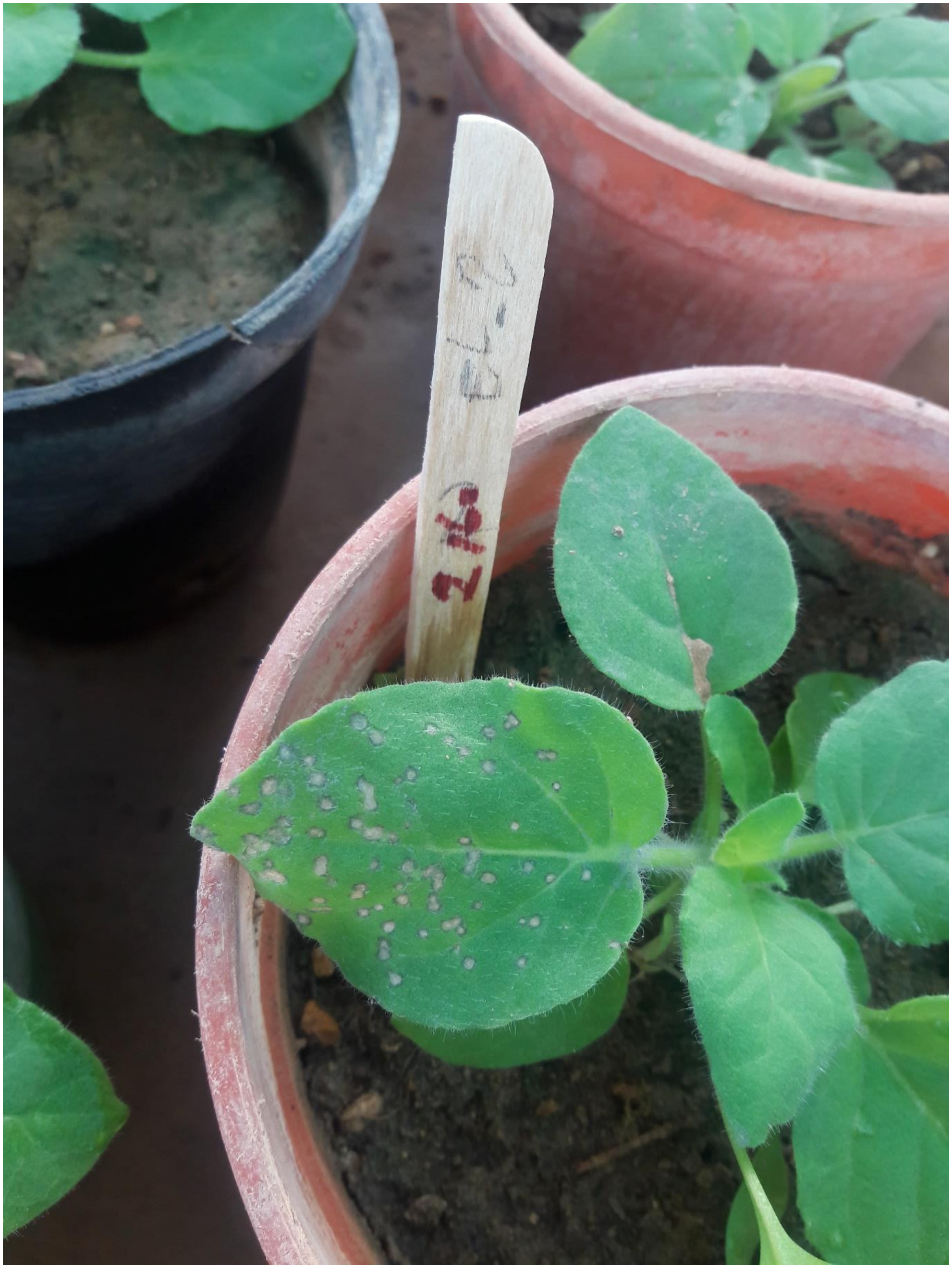

Figure 7 / c

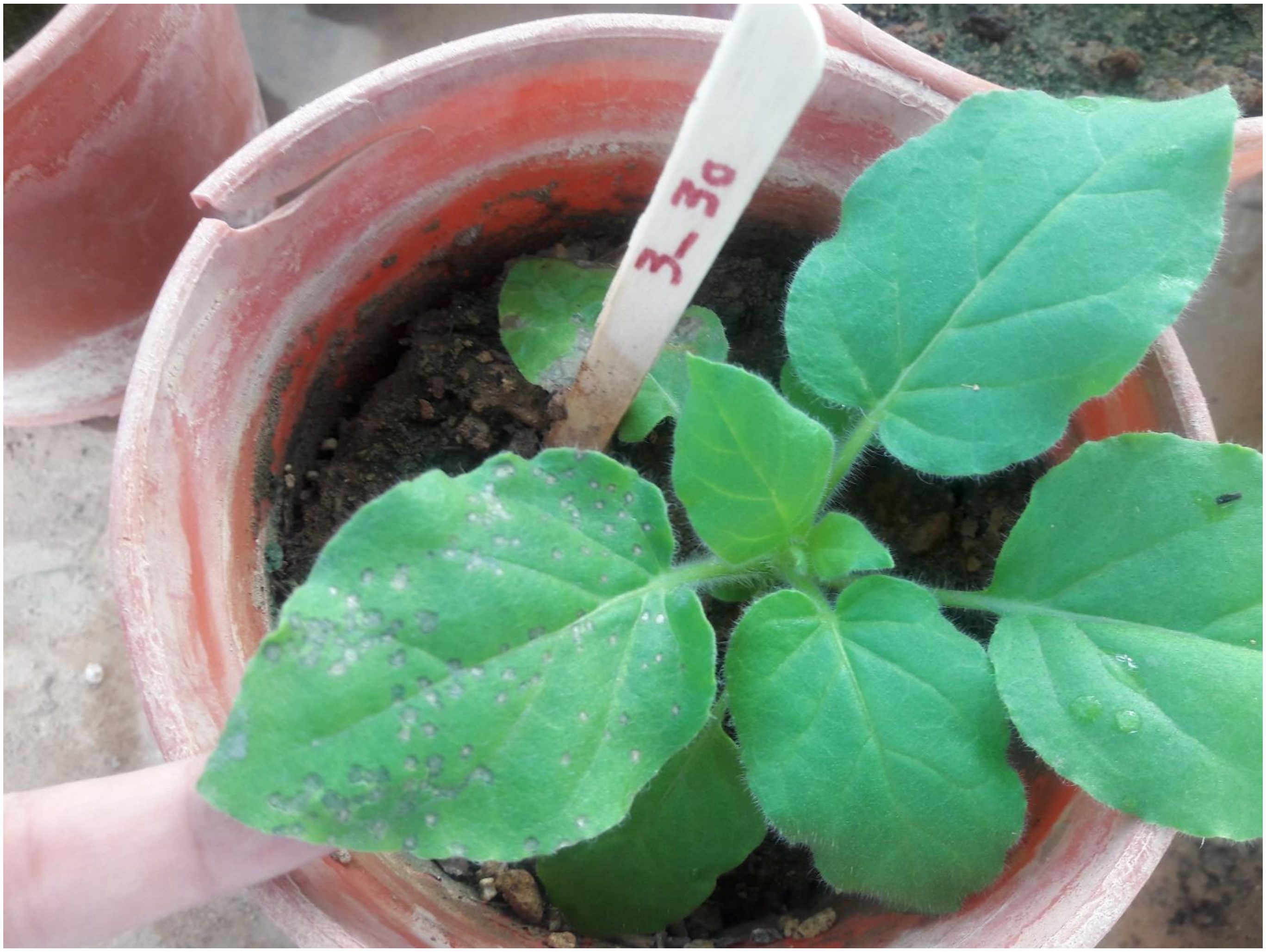

Figure 7 / d

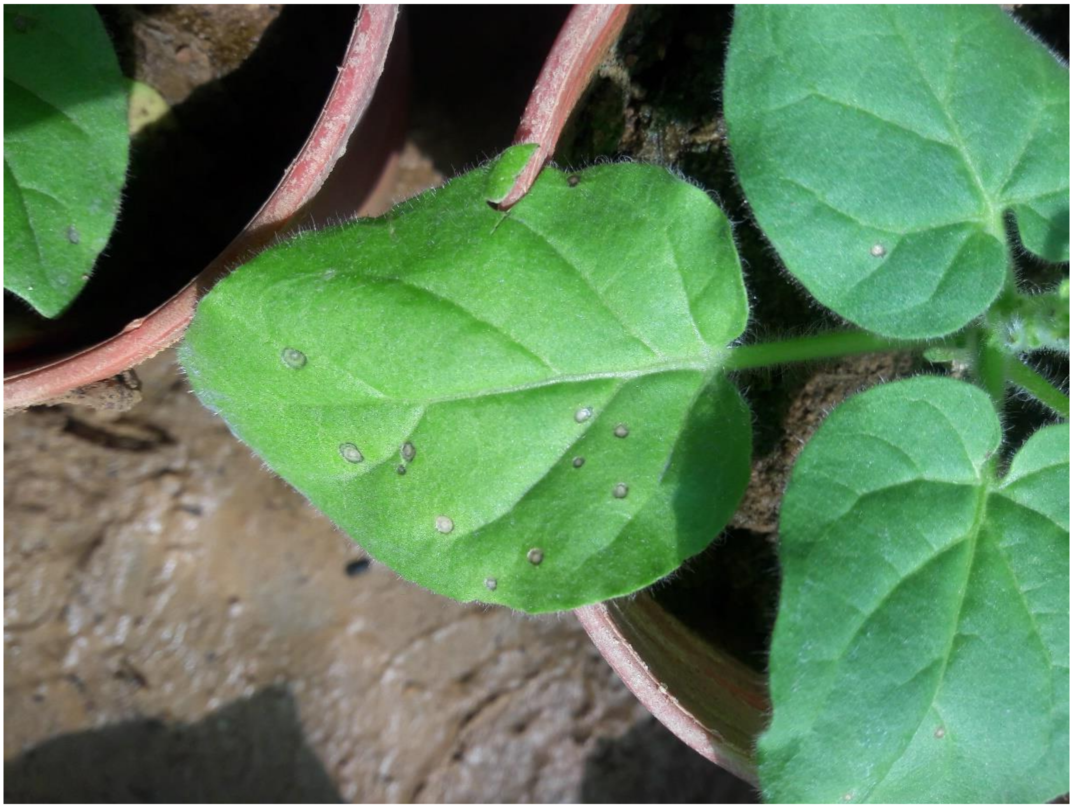

Figure 7 / e

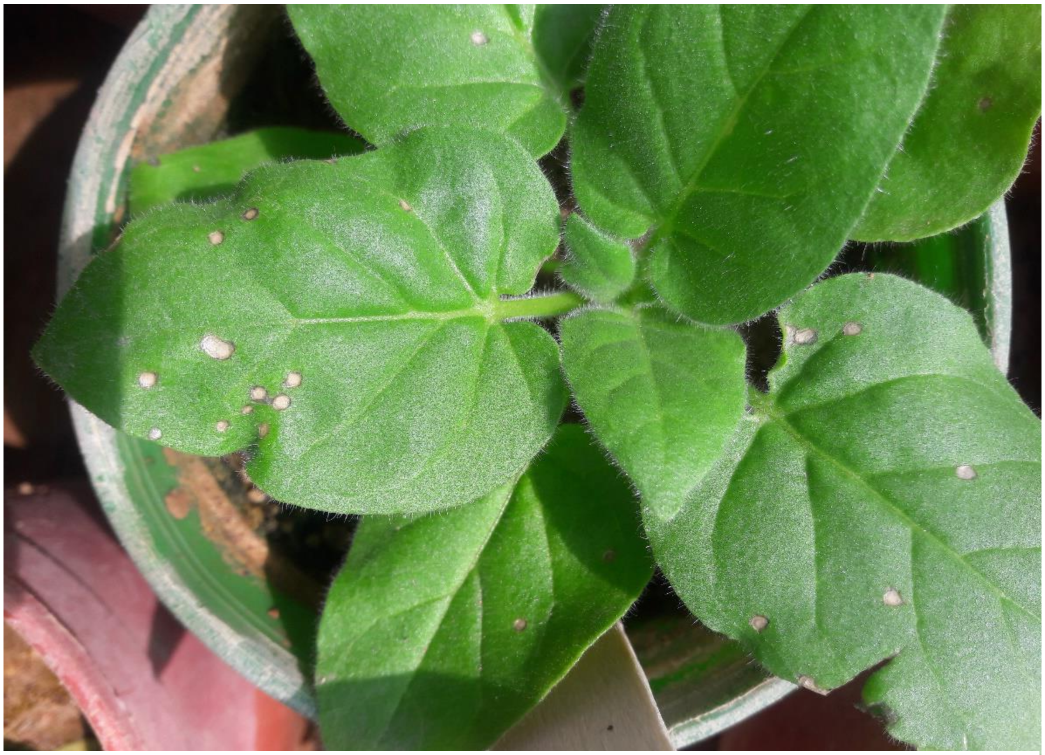

Figure 7 /f

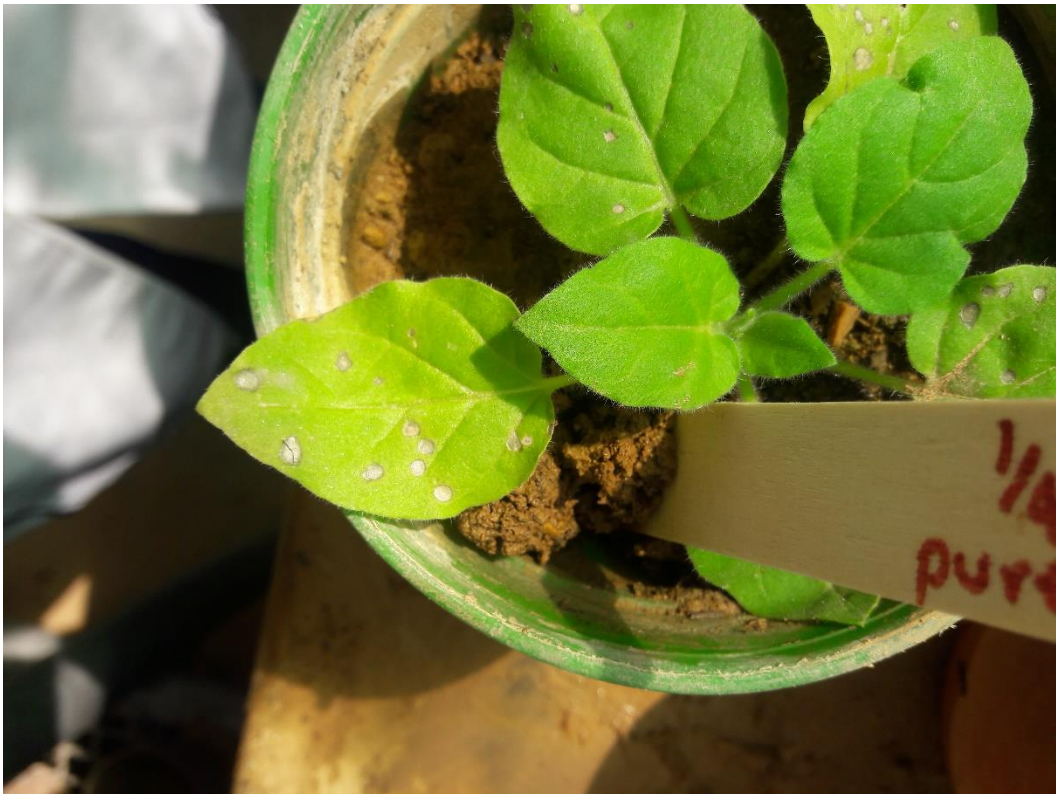

Figure 7 / g

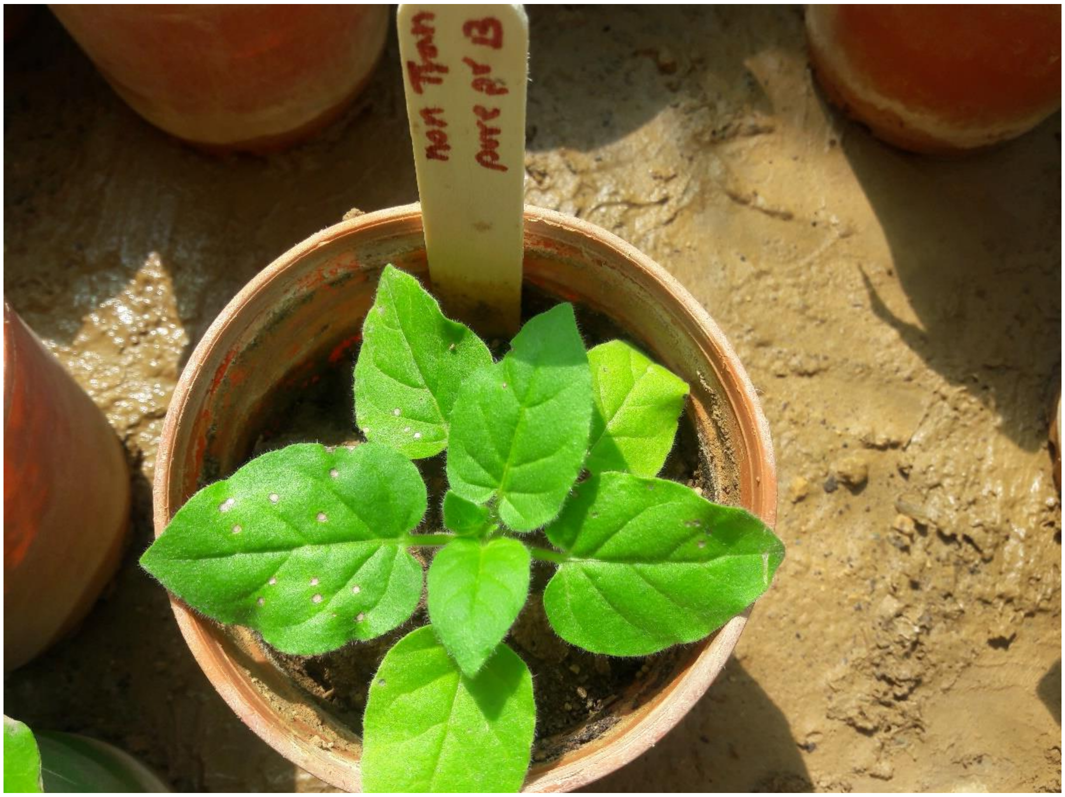

Figure 7 / h

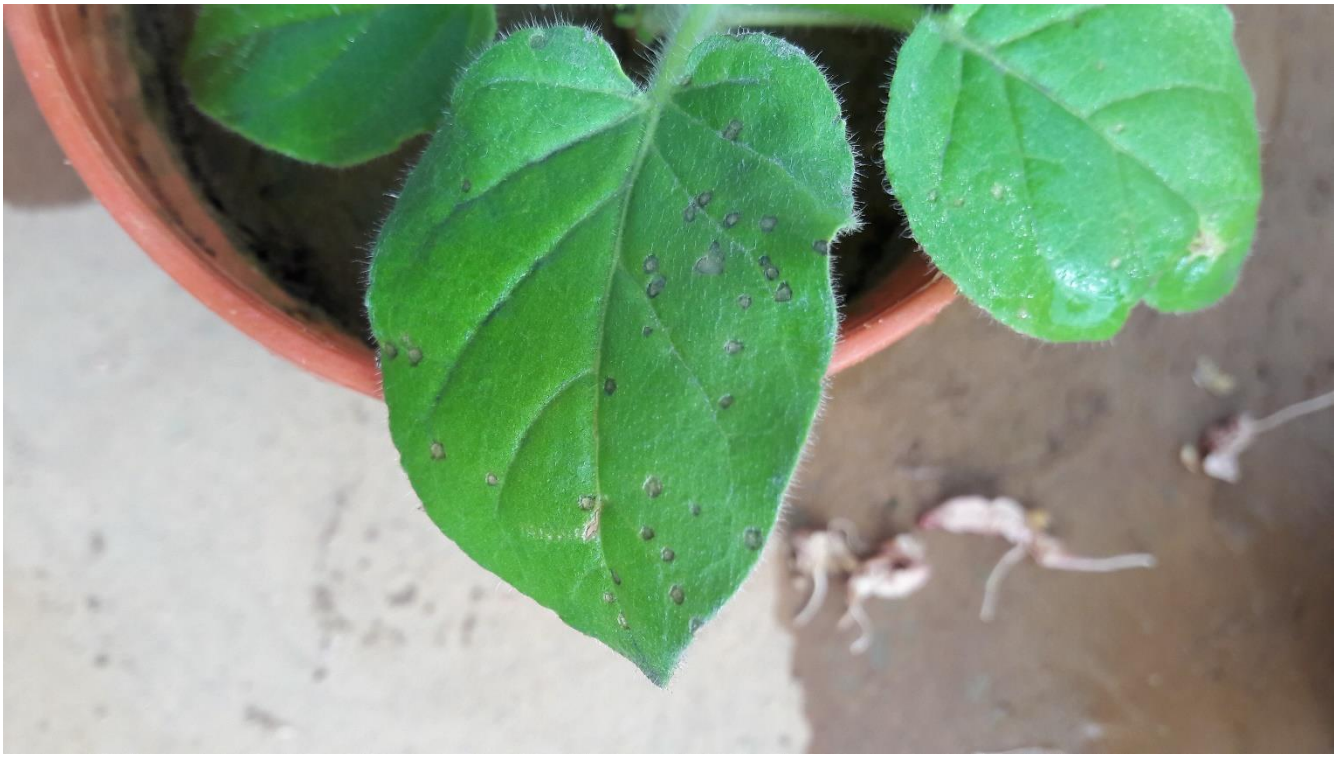

Figure8/ a-right

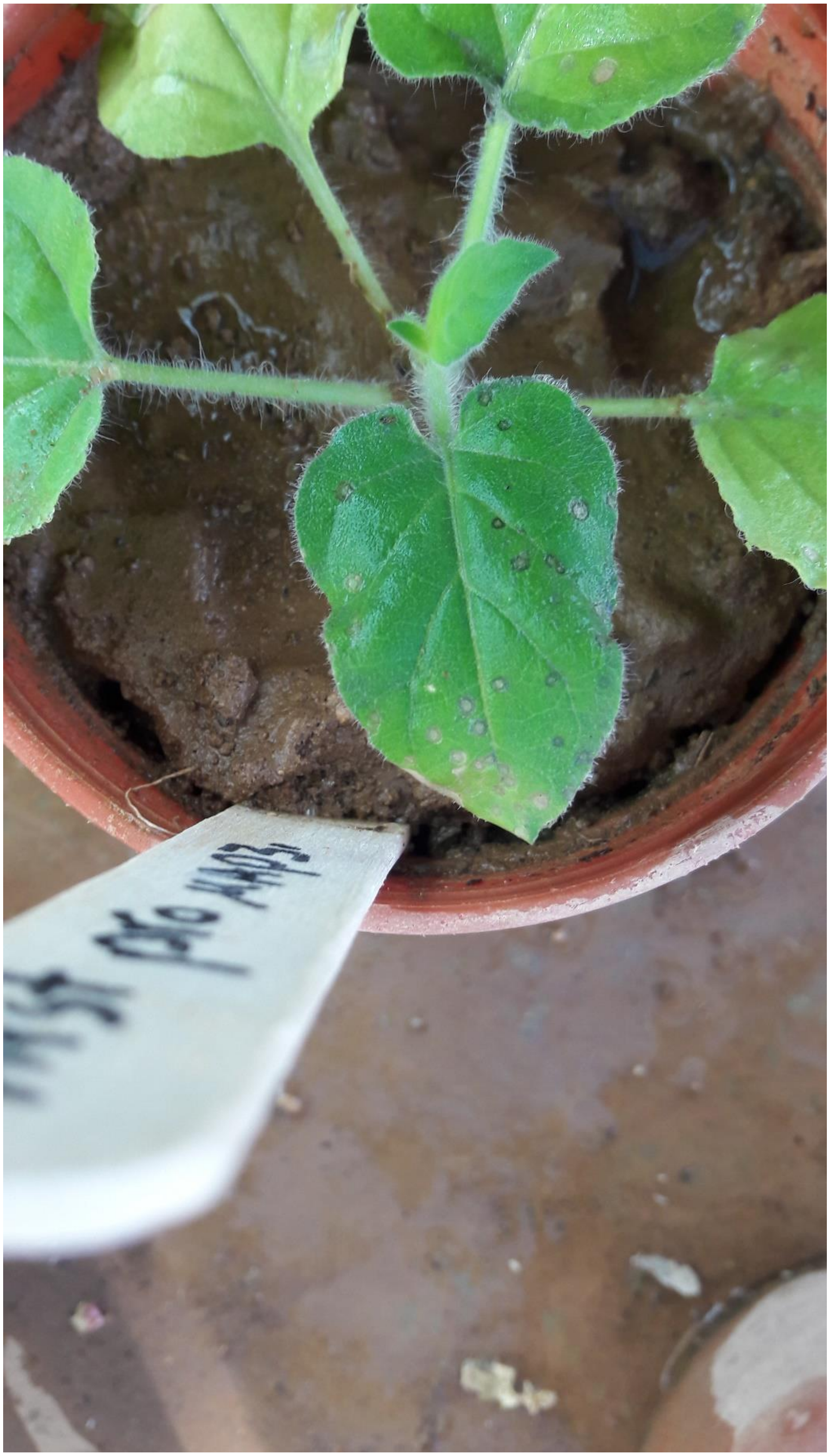

Figure 8/ c right

-

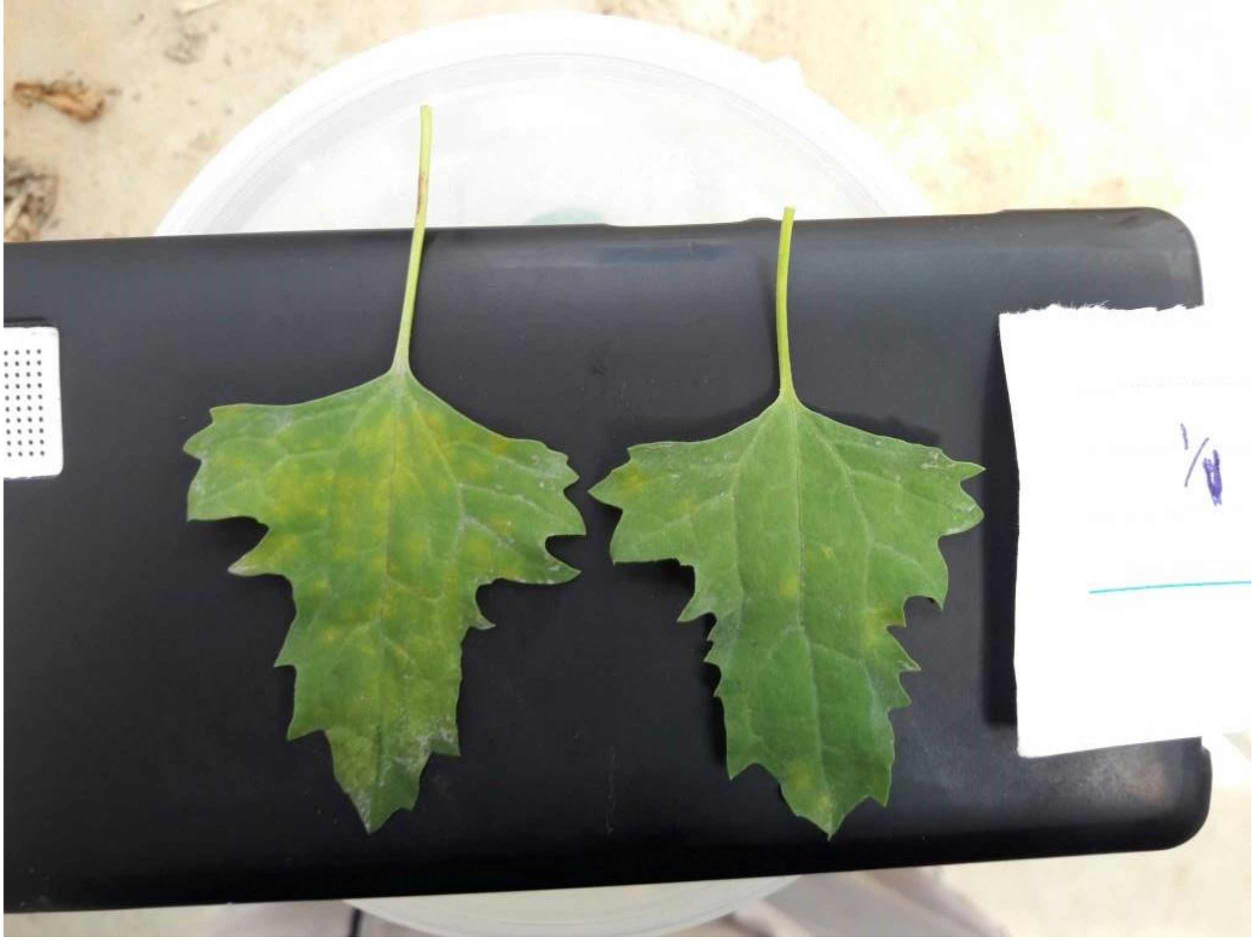

Figure 9/ a_ the left leaf was treated with virus_ the right leaf was treated with undiluted protein

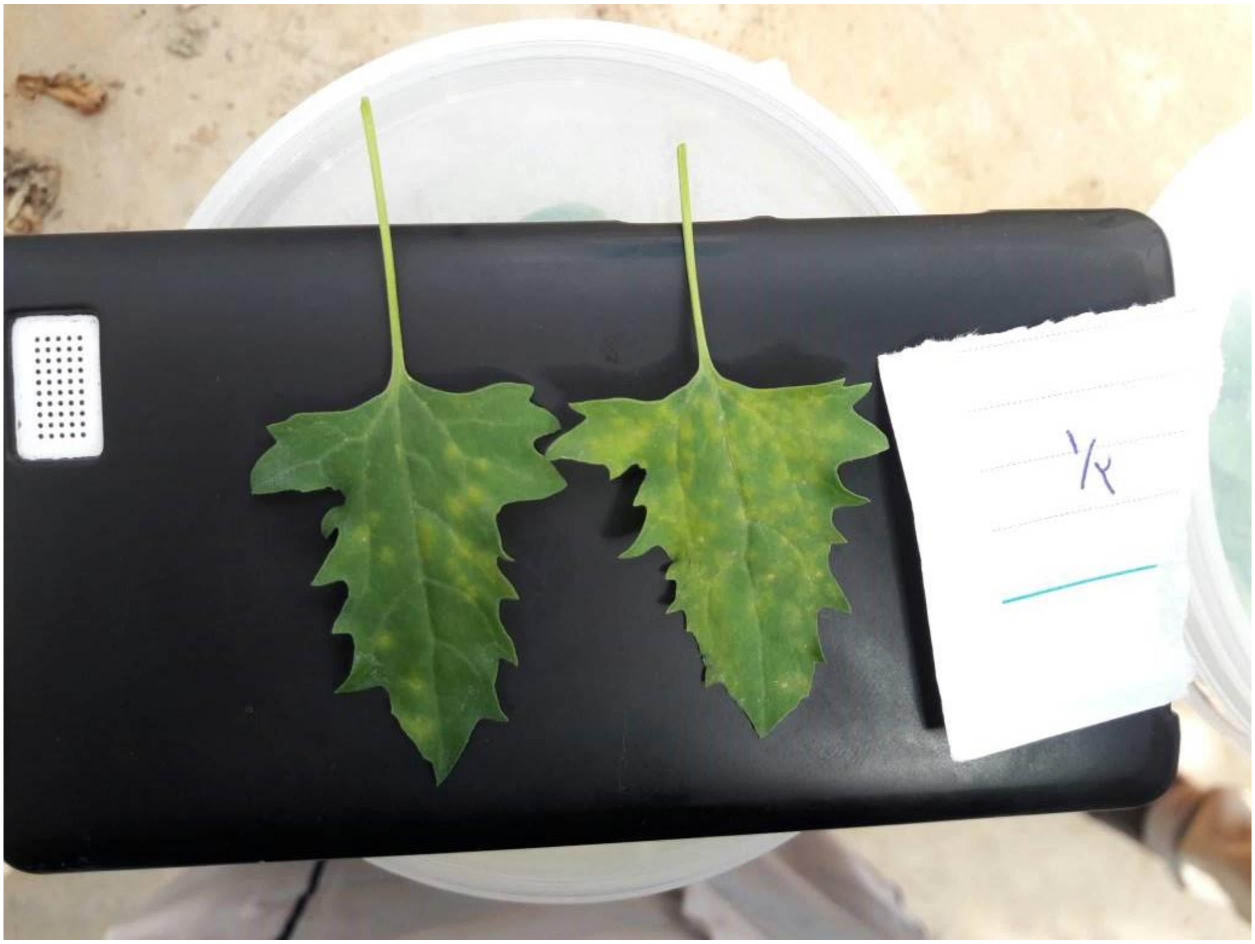

Figure 9/ b- the left leaf was treated with ½ diluted protein_ the right leaf was treated with virus

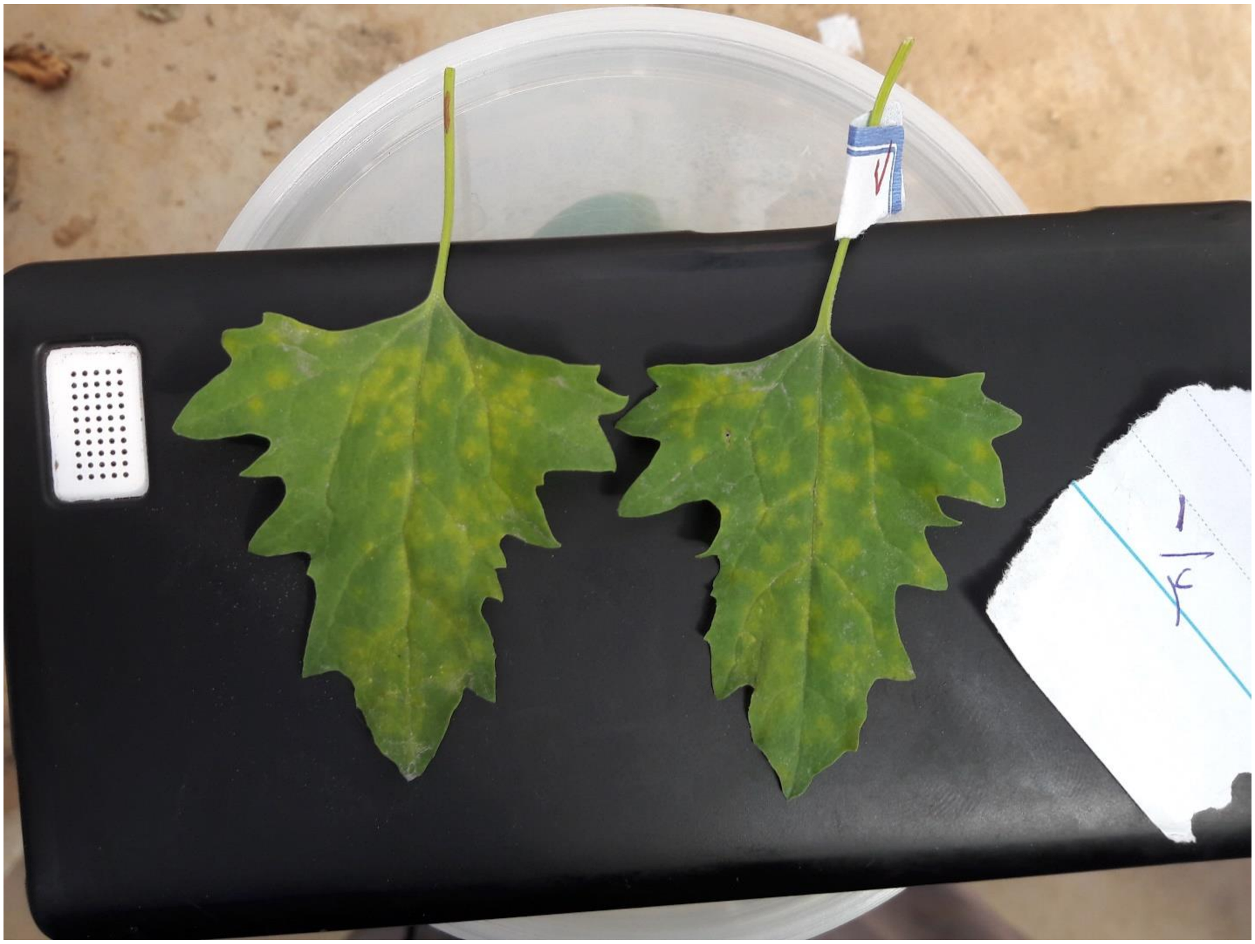

Figure 9/ C- the left leaf was treated with virus_ the right leaf was treated with ¼ diluted protein

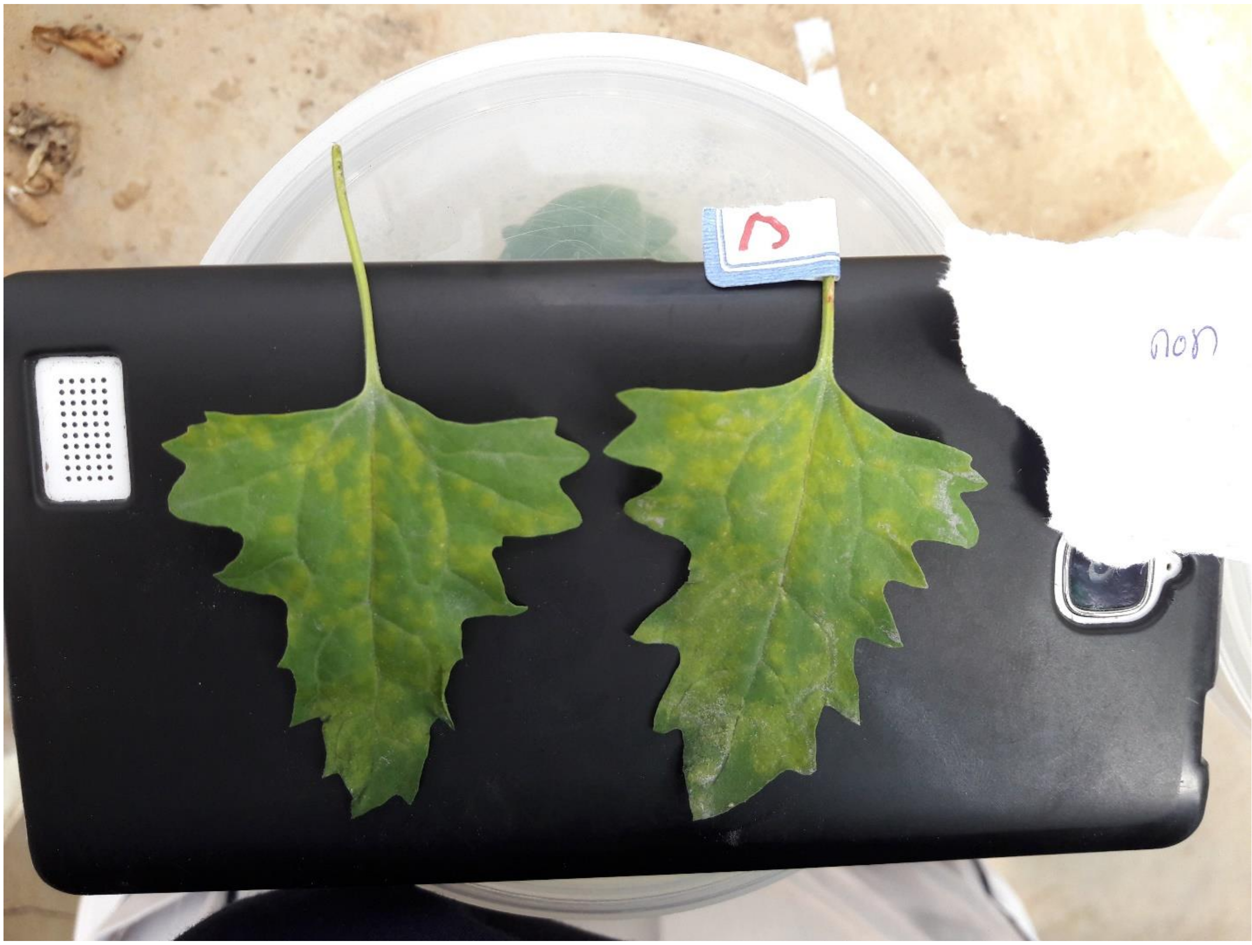

Figure 9/ D- the left leaf was treated with non-transgenic protein_ the right leaf was treated with virus

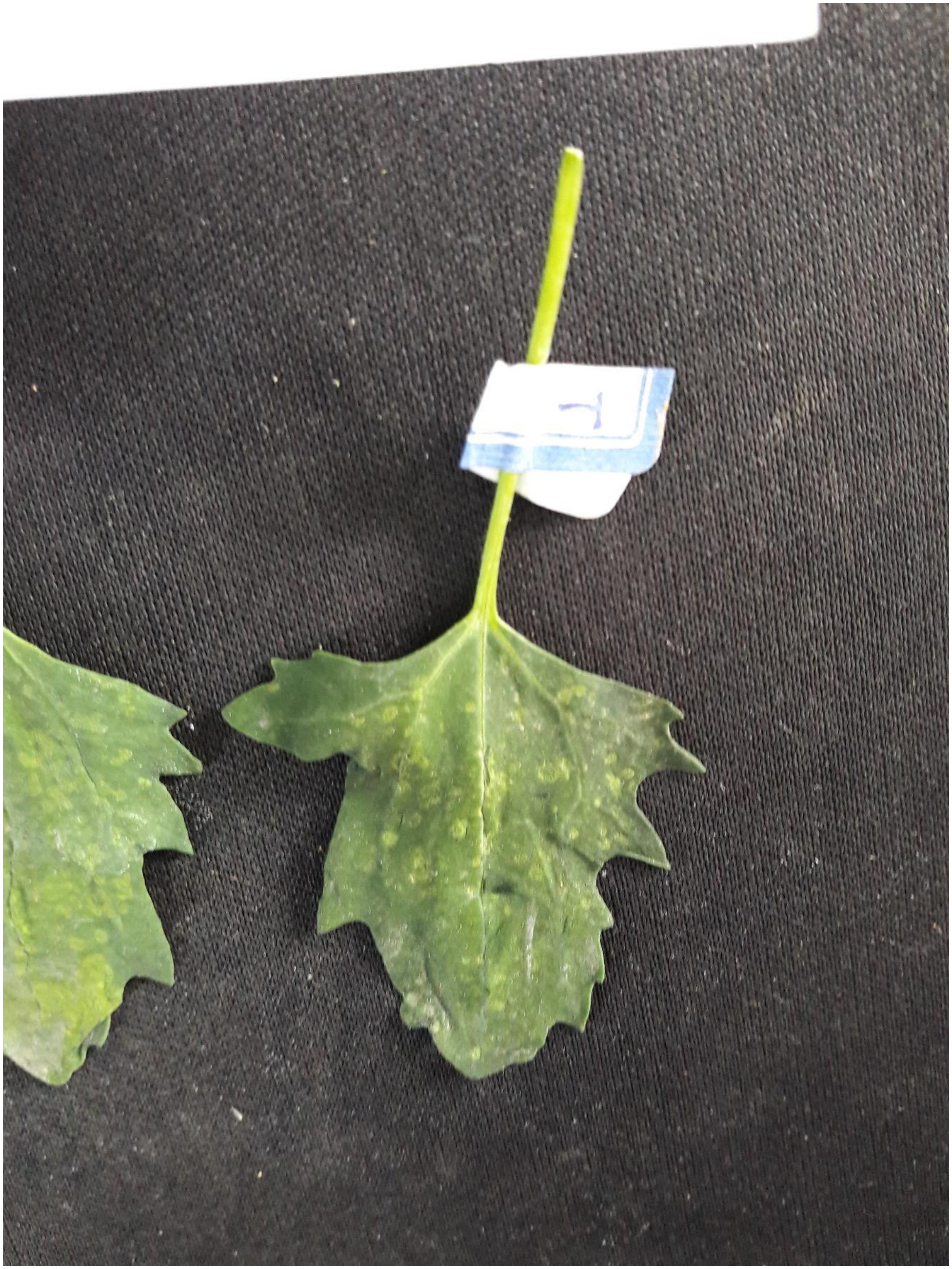

Figure 10/ T

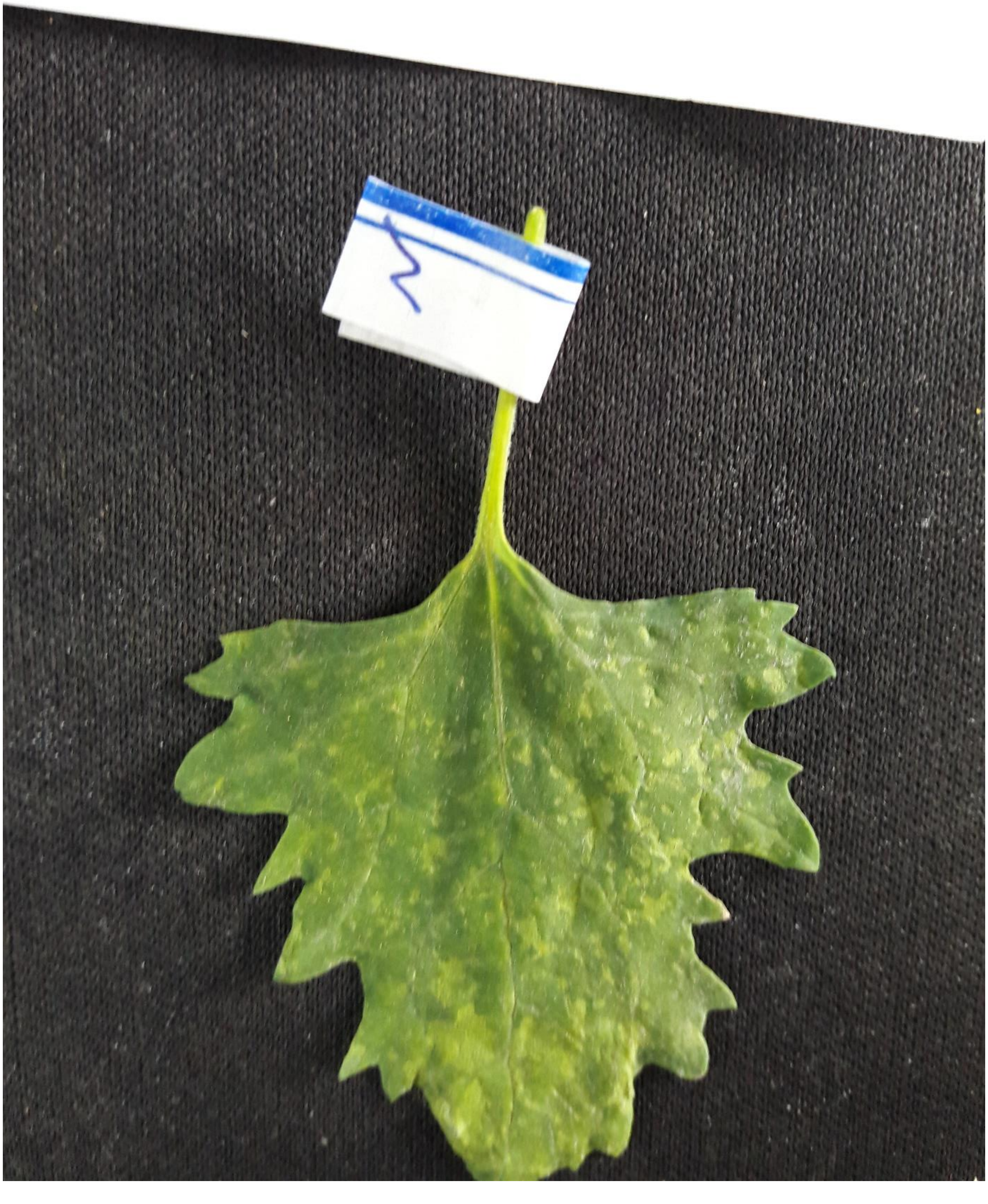

Figure 10/ N

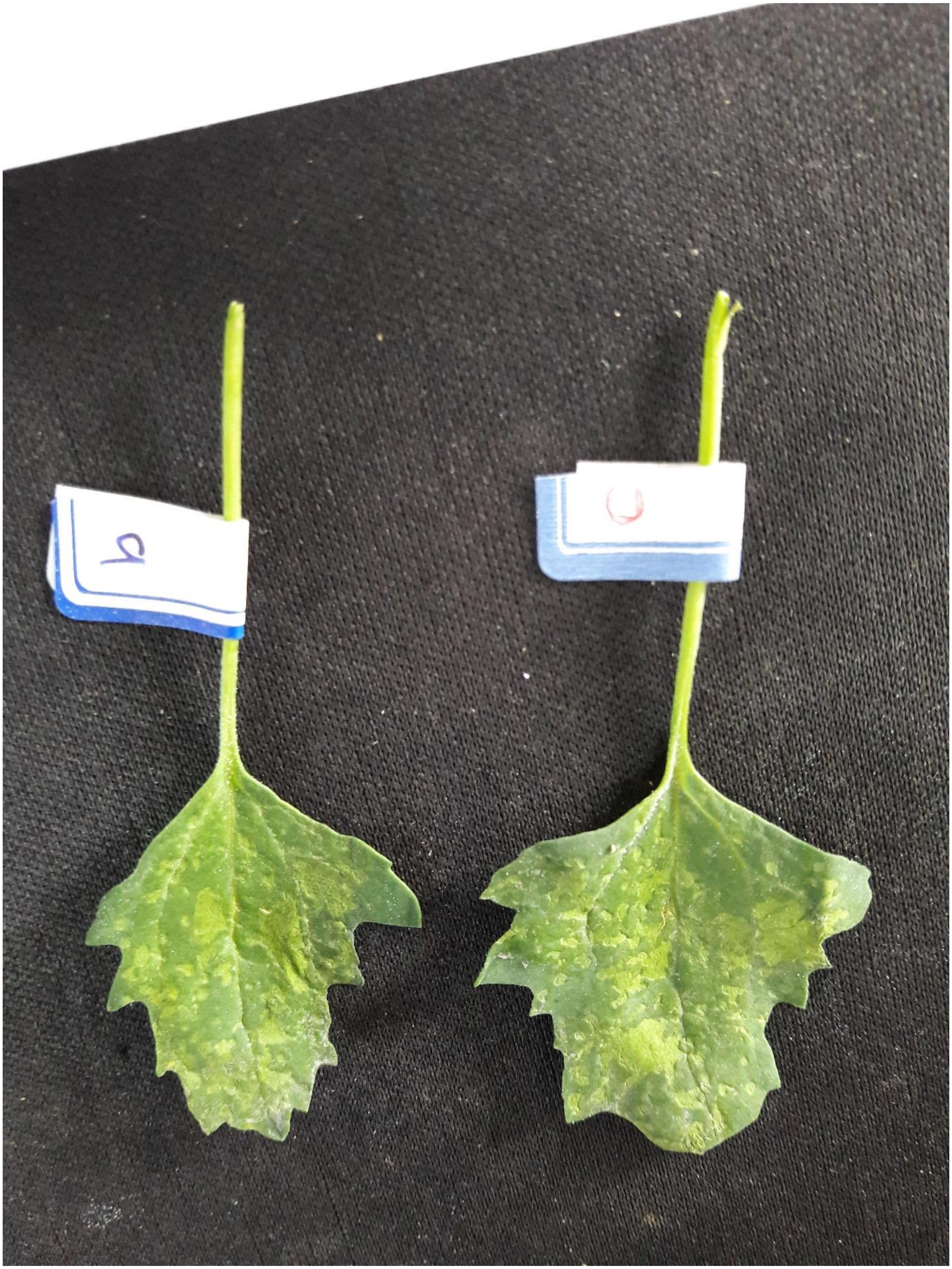

Figure 10/ B and C

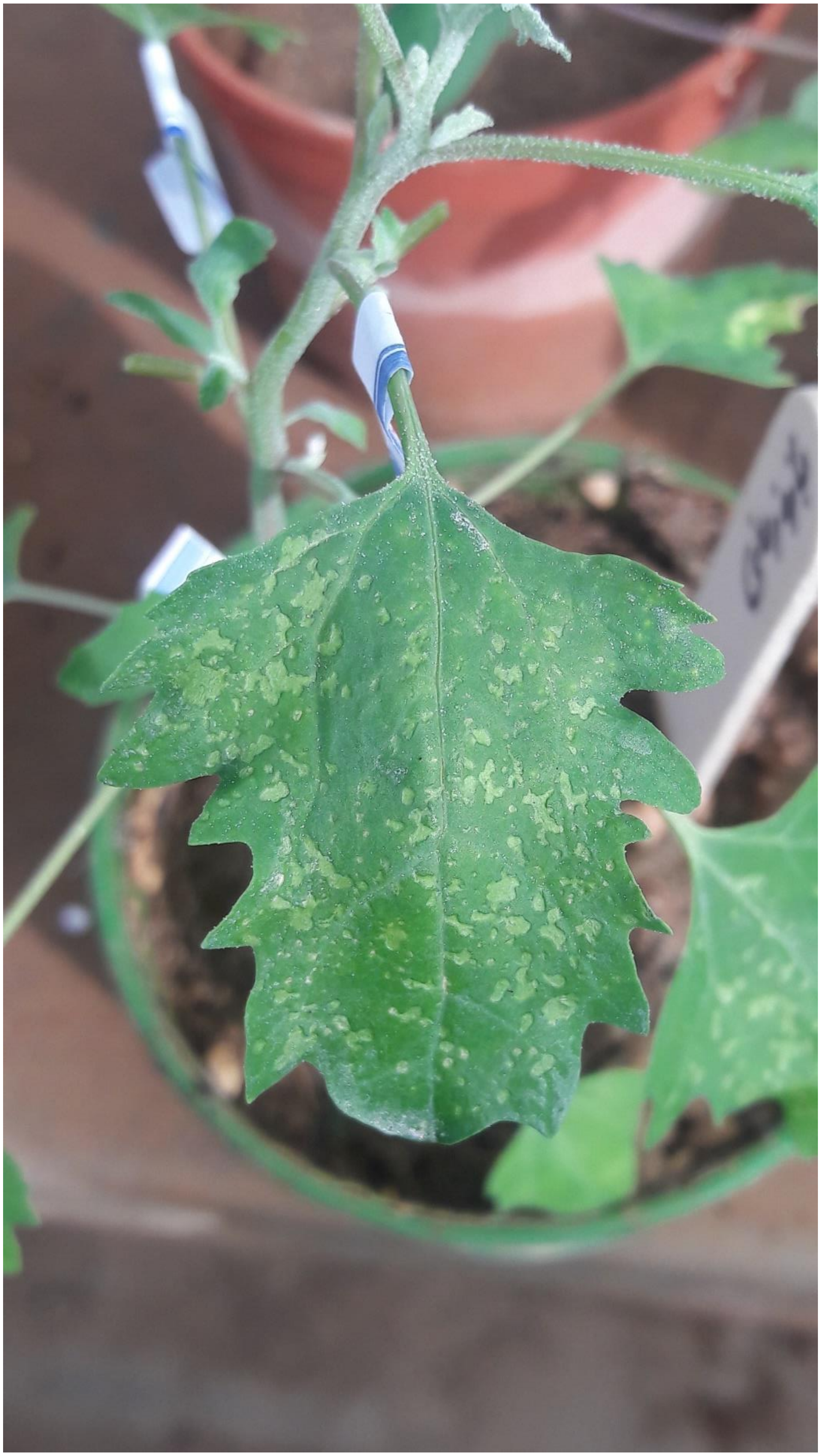

Figure 11/ a: sample C

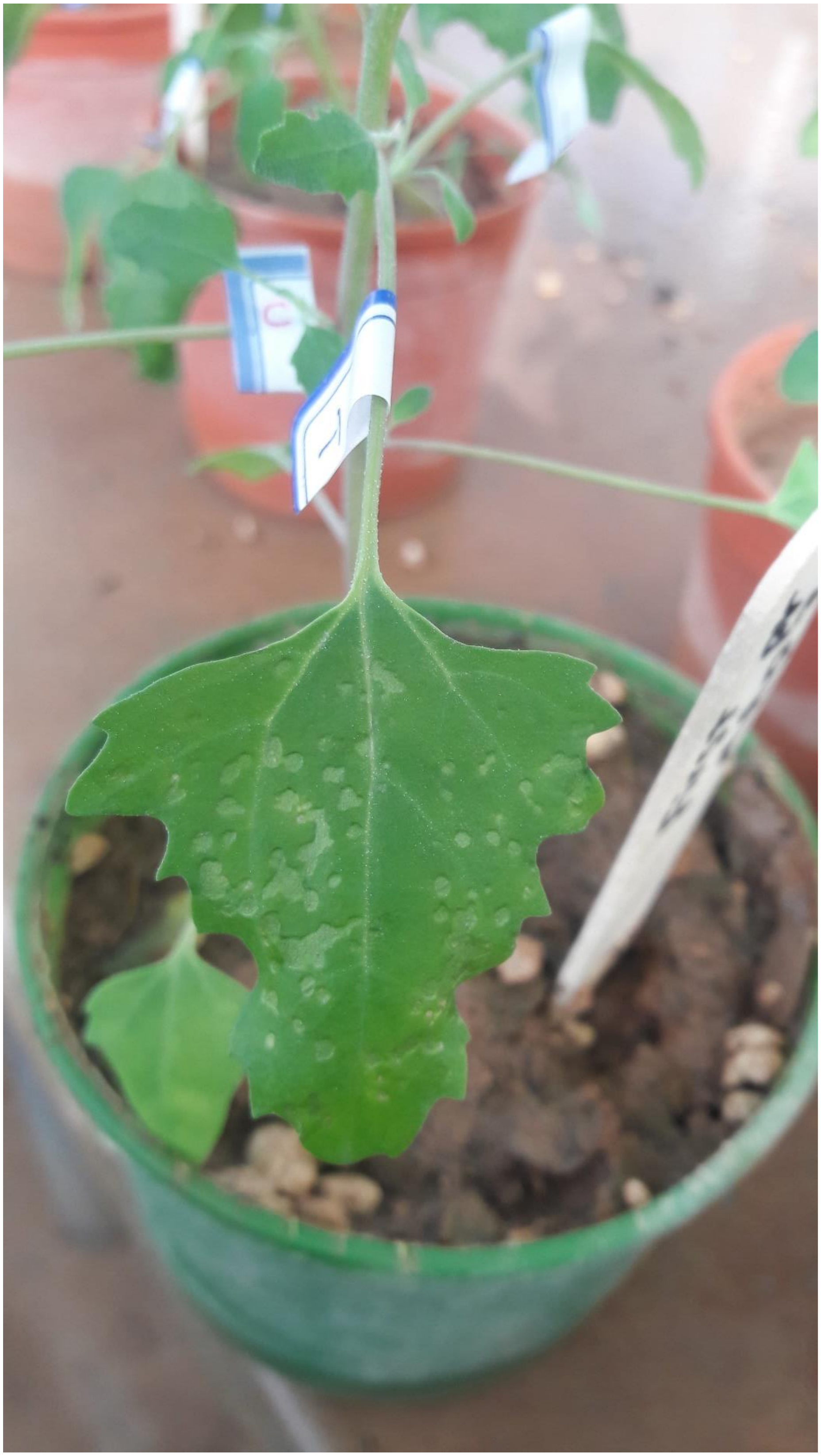

Figure 11/ a: sample T

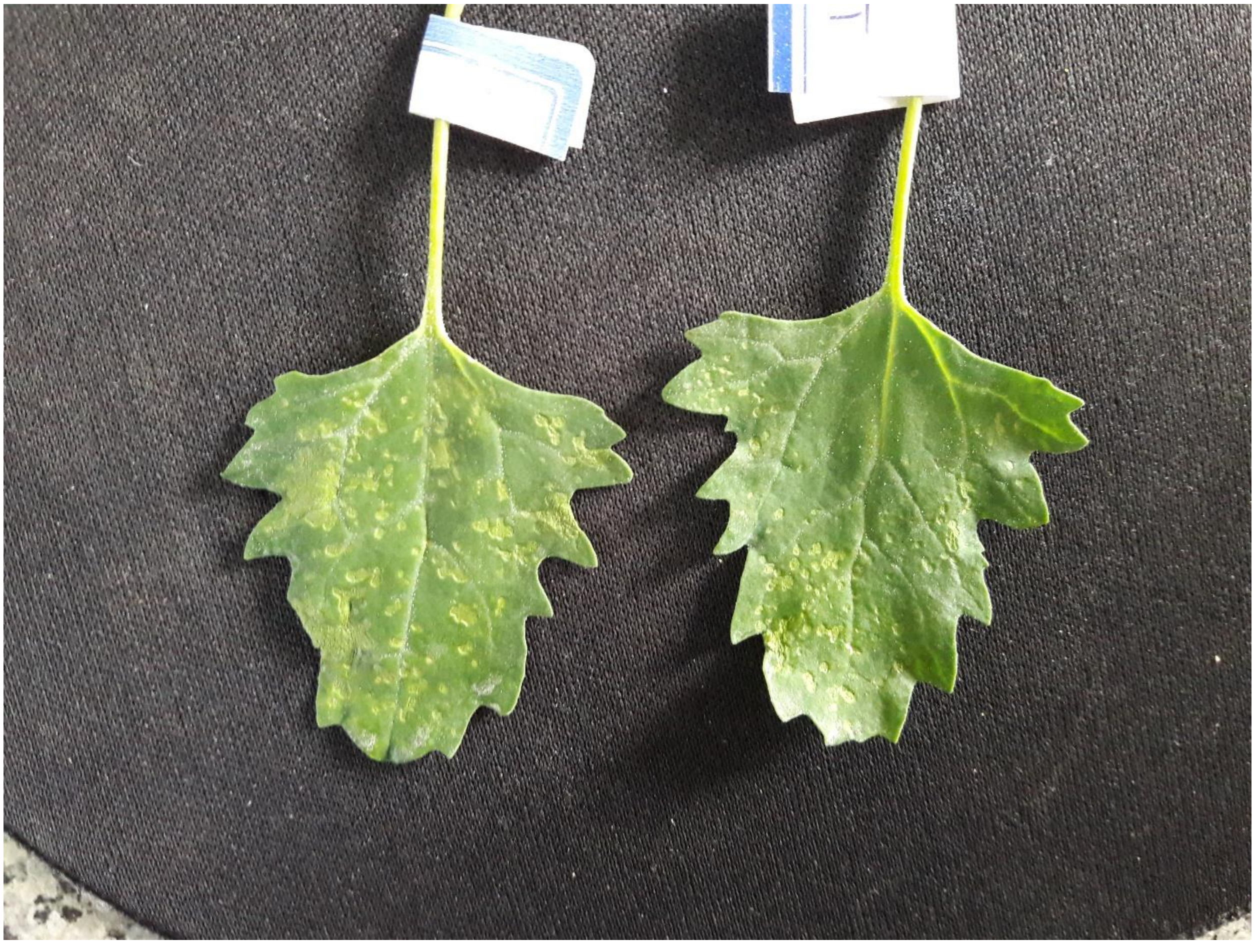

Figure 11/ b: sample C and T
